# Myofilament-associated proteins with intrinsic disorder (MAPIDs) and their resolution by computational modeling

**DOI:** 10.1017/S003358352300001X

**Published:** 2023-01-11

**Authors:** Bin Sun, Peter M. Kekenes-Huskey

**Affiliations:** 1 Research Center for Pharmacoinformatics (The State-Province Key Laboratories of Biomedicine-Pharmaceutics of China), Department of Medicinal Chemistry and Natural Medicine Chemistry, College of Pharmacy, Harbin Medical University, Harbin 150081, China; 2 Department of Cell and Molecular Physiology, Loyola University Chicago, IL 60153, USA

**Keywords:** myofilament, intrinsically disordered protein, structure and dynamics, computational modeling, cardiomyopathy

## Abstract

The cardiac sarcomere is a cellular structure in the heart that enables muscle cells to contract. Dozens of proteins belong to the cardiac sarcomere, which work in tandem to generate force and adapt to demands on cardiac output. Intriguingly, the majority of these proteins have significant intrinsic disorder that contributes to their functions, yet the biophysics of these intrinsically disordered regions (IDRs) have been characterized in limited detail. In this review, we first enumerate these myofilament-associated proteins with intrinsic disorder (MAPIDs) and recent biophysical studies to characterize their IDRs. We secondly summarize the biophysics governing IDR properties and the state-of-the-art in computational tools toward MAPID identification and characterization of their conformation ensembles. We conclude with an overview of future computational approaches toward broadening the understanding of intrinsic disorder in the cardiac sarcomere.

## Purpose and scope

The contractile cells of the heart rely on myofilament proteins that transduce a chemical trigger, calcium (Ca^2+^), into mechanical force. The myofilament proteins form macromolecular assemblies that perform diverse structural, functional, and regulatory roles. While the composition of these assemblies and their three-dimensional structures continue to be resolved, a high percentage of myofilament proteins contain intrinsically disordered region (IDR)s that do not easily lend themselves to conventional structure determination techniques, such as X-ray crystallography or nuclear magnetic resonance (NMR) spectroscopy. We introduce the term myofilament-associated protein with intrinsic disorder (MAPID) to refer to those myofilament proteins that contain IDRs. We also use the term intrinsically disordered protein (IDP) to refer to proteins that are predominantly unfolded to distinguish them from folded proteins with regions of disorder. Studies and reviews to date have largely acknowledged the existence of IDRs in these proteins, though some reports have gone further to examine how IDRs tune filament protein-binding affinities (Uversky et al., 2011) and contribute to cardiomyopathy (Na et al., 2016). However, the IDRs in the majority of MAPIDs are not characterized in detail and thus their roles in myofilament function are largely unexplored. As such, determining the structure/function relationships of these MAPIDs is a final frontier in understanding myofilament physiology.

Experimental and computational methods to structurally and functionally characterize IDPs and IDRs of arbitrary origin have exploded in growth in the last decade. Reviews of IDP structure and structure determination have thus grown in popularity in recent years (Gibbs and Showalter, 2015; Schramm et al., 2019), but they have not been interpreted in the context of proteins essential to heart cell contraction. The purpose of this review therefore is to highlight recent advances in computational approaches developed for, or could be applied, to determining the structures and functions of MAPIDs. We divide this review into two parts: (1) A summary of IDRs in a broad ensemble of myofilament proteins (section ‘Myofilament-associated protein with intrinsic disorder (MAPID)s’) and (2) computational modeling techniques that have been, or could be, applied to MAPIDs (section ‘Computational methods for predicting conformation ensembles of isolated MAPIDs’ to section ‘Computational methods for predicting the MAPID co-assembly’). We emphasize the current state-of-the-art in the computational modeling of myofilament IDPs that have been published in the last five years, where possible, although some older studies are included for context. These innovations are introduced in parallel with high-level discussions of complementary experimental techniques.

## Part 1: myofilament-associated protein with intrinsic disorder (MAPID)s

In this part, we introduce myofilament proteins and their roles in cardiac function. We next overview the proteins most commonly associated with the myofilament, and the propensity of IDRs in those proteins. Thereafter, we discuss how these IDRs influence myofilament function and dysfunction, as well as prominent challenges in characterizing their properties.

### The physiology of myofilament proteins

#### Molecular function of myofilament proteins

**Fig. 1.**
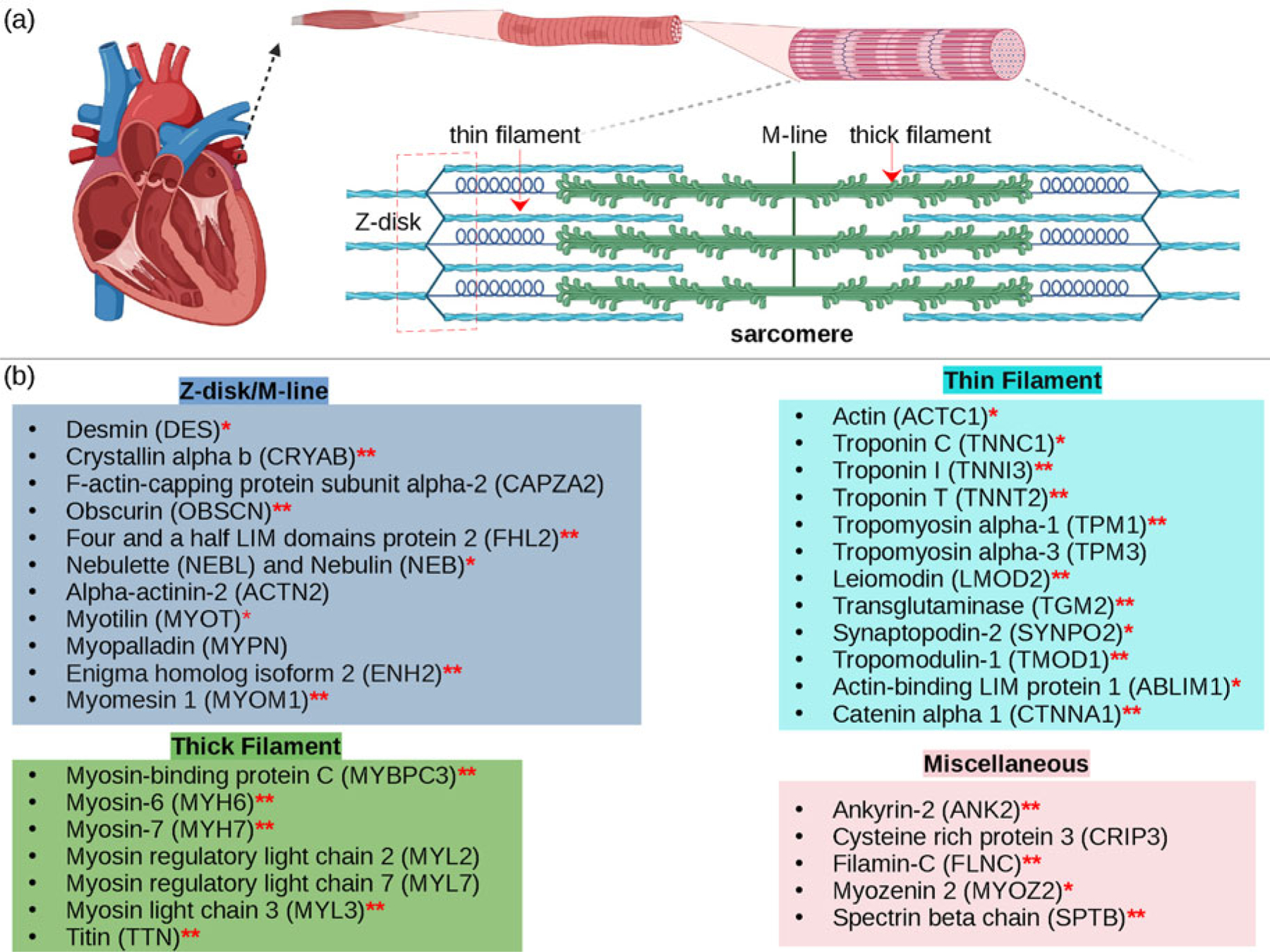
(*a*) Schematic illustration of the sarcomere (drawn with BioRender). (*b*) In this review, we focus on the cardiac proteins proposed in Kooij *et al*. (2014) with some additional noteworthy examples. For proteins with multiple isoforms, only isoforms with spectra counts (SC) >10 were selected. Proteins with IDR(s) are indicated by red *. Double ** indicates that the IDR(s) has been experimentally confirmed for the gene, while a single * indicates that the confirmation was based on a related isoform or via bioinformatic predictions (see Table 1 for details).

**Fig. 2.**
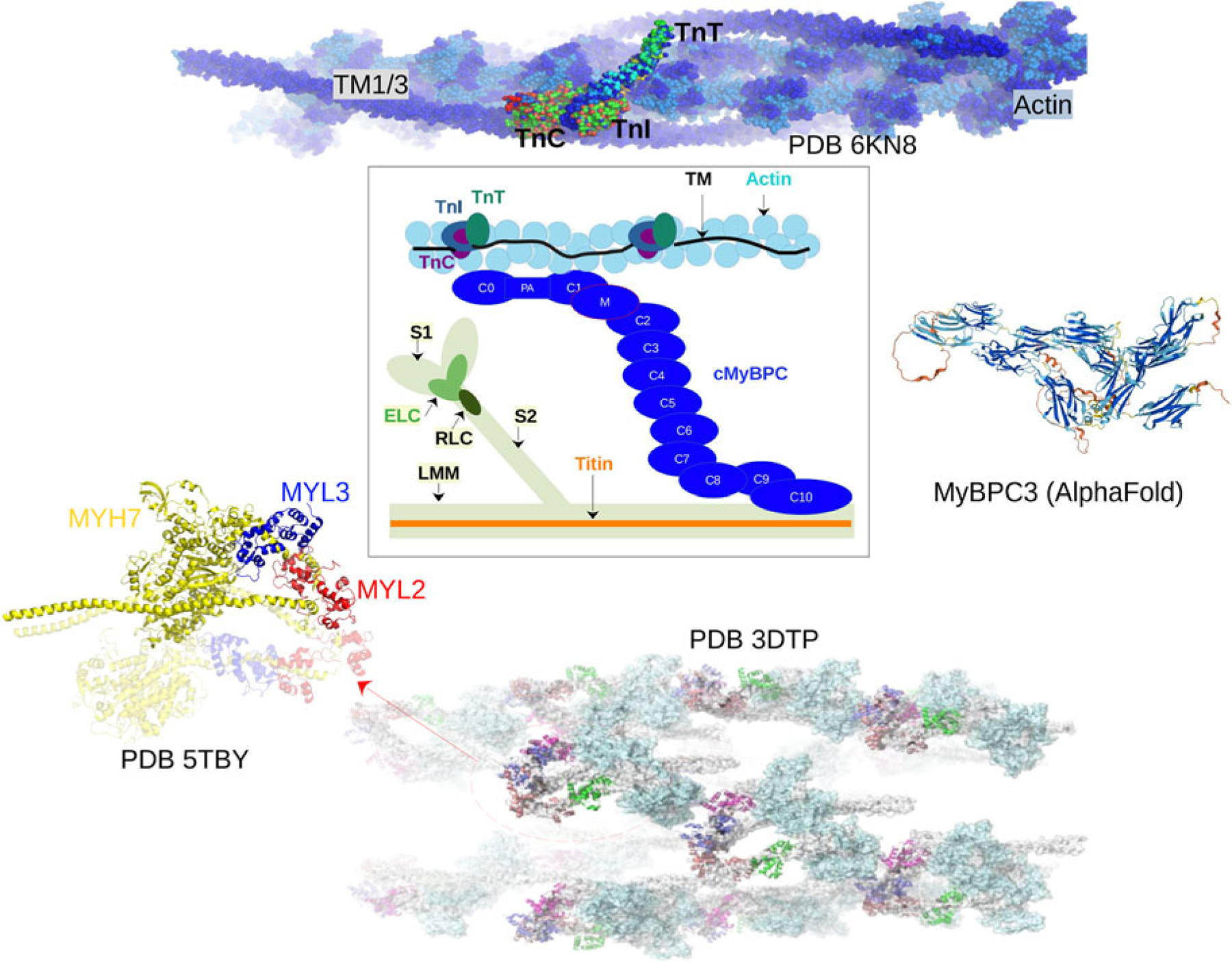
Core proteins of the thin and thick filament, based on the schematic from Harris *et al*. (2011). The thin filament structure PDB 6KN8 was constructed from a cryo-EM study (Yamada *et al*., 2020). PDB 5TBY was generated from homology modeling. The MyBPC3 structure was predicted by AlphaFold and was downloaded from the UniprotKB database. A 2 nm resolution model of tarantula thick filament was built by fitting atomistic component structures to EM density map (PDB 3DTP (Alamo *et al*., 2008)).

Cardiac contraction is driven by the concerted activity of myofilament proteins that contract the sarcomeres of the cell (see Fig. 1). Although the major protein components of the sarcomere have been identified, the composition of the sarcomere is dynamic (Willis et al., 2008). For this reason, myofilament protein isoform expression can vary during development and in response to pathological stimuli (Marston and Redwood, 2003). Therefore, we limit the scope of this review to the myofilament-associated genes of the adult rat cardiac myofilament reported in Kooij et al. (2014) and depicted in Fig. 1. Myofilament proteins can be loosely divided into those belonging to the thin filament (section ‘MAPIDs of the thin filament’), the thick filament (section ‘MAPIDs of the thick filament’), and Z-disk (section ‘MAPIDs of the Z-disk’). The thick filaments are formed from the inter-twining tails of myosin protein dimers (see representative structure in Fig. 2). These myosins bind to actin monomers of the thin filament, upon which energy released by the hydrolysis of adenosine triphosphate (ATP) is used to generate mechanical force. The thin filament comprises approximately 15 actin (ACTC1) monomers, two troponin macromolecules, and two tropomyosin chains that together form the repeating contractile unit of the sarcomere (Yamada et al., 2020). Thin filament proteins primarily sense elevated intracellular Ca^2+^ following an action potential to unveil binding sites on actin for myosin. The thin filaments of adjacent sarcomeres are joined by proteins that form the Z-disk. In addition to forming a scaffold for myofilaments, Z-disk proteins are subject to, and perform, sundry regulatory roles that help adapt sarcomere force generation to demand. Several proteins including myosin-binding protein C (MyBPC3) and nebulin bridge filaments or link the Z-disk to filaments, which are also discussed in sections ‘MAPIDs of the thin filament’ and ‘MAPIDs of the Z-disk’. Excellent reviews on myofilament proteins and their functions include Russell and Solís (2021) and others (Sols and Solaro, 2021), albeit with limited discussion of their intrinsic disorder.

Contraction begins with the resting sarcomere. In that state, the myosin binding sites on the thin filament are mostly blocked by tropomyosin at resting Ca^2+^ levels during diastole (ca. 100 nM) (Clapham, 2007). Activation of calcium channels on the plasma membrane and sarcoplasmic reticulum (SR) following depolarization of the cell conducts Ca^2+^ and thereby rapidly increases the intracellular Ca^2+^. TnC, a Troponin (Tn) protein, binds to one equivalent of free Ca^2+^, which exposes a hydrophobic domain on its N-terminus. The exposed domain provides a binding site for the TnI C-terminus. TnC/TnI binding leads to shifts in the positions of TnT and tropomyosin (Tm). As Tm slides along the actin filament (Rynkiewicz et al., 2015), binding sites on actin for myosin are unveiled. Actin-bound myosin results in what is commonly called a cross-bridge. Following thin filament activation, cross-bridge formation and ATP hydrolysis generate force that contracts sarcomere. Thin filament activation and cross-bridge formation are highly cooperative, that is, the activation of one contractile unit facilitates the activation of its neighbors. Restoration of diastolic calcium level via SR Ca^2+^ pumps and plasma membrane (PM) ion exchangers ultimately returns the sarcomere to its relaxed state. Many genes beyond those named here (see Fig. 1) couple the Z-disks and filaments, as well as tune the sarcomere’s responsiveness to Ca^2+^, stretch, and external forces (namely those arising from the filling of ventricles and atria).

Sarcomere contraction is tightly regulated on a beat-to-beat basis and dynamically adapts to demands on cardiac output. Rapid regulation is afforded through post-translational modification (PTM) of myofilament proteins that include phosphorylation, oxidation, ubiquitination, acetylation, and methylation among others (Liddy et al., 2013; Jin et al., 2019). To date, phosphorylation is likely the best understood of myofilament PTMs. Many sites are suggested for titin (999 sites), myosin (87 sites for MYH6 and 126 sites for MYH7), TnI (20 sites), and MyBPC3 (35 sites) based on our queries of the PhosphoSitePlus database (Hornbeck et al., 2015). Phosphorylation most commonly occurs through the kinases Ca^2+^/calmodulin-dependent protein kinase II (CaMKII) and protein kinase A (PKA), as well as protein kinase C (PKC) (DeSantiago et al., 2002; LeWinter, 2005; Hidalgo et al., 2013). PKA generally increases force by potentiating Ca^2+^ release and enhances relaxation by decreasing the Ca^2+^-sensitivity of force generation, while PKC typically opposes these changes (LeWinter, 2005); CaMKII is implicated in accelerating the rate of relaxation while the heart is pacing rapidly (DeSantiago et al., 2002). However, these adaptations are sensitive to the specific sites that are phosphorylated. Recent applications of high-throughput mass spectrometry are revealing a host of other PTM modalities in myofilament proteins (Jin et al., 2019), including acetylation, methylation, oxidation by reactive oxidation species, and conjugation with sundry other biomolecules. Unlike myofilament protein phosphorylation, these PTMs and their impact on contractility are less understood. As will be later discussed, PTMs are common in IDRs of myofilament proteins.

Other regulatory changes include cellular and tissue adaptations to demands on cardiac output that generally occur more slowly than those afforded by PTMs. Hypertrophic adaptations, for instance, result in enlargement of the heart due to physiological drivers (development, exercise, and pregnancy) and pathophysiological (congenital defects, disease and infection, lifestyle or adverse environmental) conditions. The heart may also undergo atrophic changes that reduce the heart size and under pathophysiological conditions, the thinning of cardiac tissue, which altogether decrease cardiac output. These adaptations can entail the up- and down-regulation of myofilament proteins including their isoforms (Bers, 2001), and changes in the number and assembly of sarcomeres (Martin and Kirk, 2020). While important, these modalities of regulation generally impact the number and organization of myofilament proteins, not their intrinsic properties, and are thus beyond the scope of our review.

Cardiac disease remains one of the most prolific causes of death. The majority of etiologies correspond to pathological adaptations to diet or sedentary lifestyle (Forman and Bulwer, 2006), although hereditary origins to congenital defects are also common. At the myofilament protein level, cardiac disease can be accompanied by dysregulated contractility, such as altered Ca^2+^ sensitivity (Messer and Marston, 2014), kinetics of force generation (Belus et al., 2008), maximum contractile force (Crocini and Gotthardt, 2021), and cooperativity (Ramirez-Correa et al., 2015). Genetic causes or susceptibilities can include missense mutations in myofilament proteins or translational defects (Xu et al., 2010; Mazelin et al., 2016). As an example, the cardiac myosin isoforms MYH7 and MYH6 have 58 and 3 disease-associated variants, respectively, and another 247 and 10 variants of unknown significance (VUSs) based on the ClinVar database (Landrum et al., 2013).^1^ A variety of these mutations exhibit loss- or gain-of-function at the protein-level (Moore et al., 2012), which stem from impacts on myosin’s intrinsic properties or its interactions with other myofilament proteins. Similarly, PTMs within its IDRs can also contribute to myosin dysfunction (Mahmud et al., 2021). While gain- and loss-of-function phenotypes at the protein level are unlikely to explain all aspects of a pathological phenotype (Ono et al., 2020), studies of IDRs are instructive for understanding mechanisms of myofilament dysfunction.

#### Myofilament proteins and structure determination efforts

Determining the structures of myofilament proteins at atomistic resolution is an important preliminary step in uncovering their functional roles. The prevalence of well-folded myofilament-associated proteins has enabled hundreds of structural studies via X-ray crystallography, NMR, cryo-electron microscopy (cryo-EM), and small-angle X-ray scattering (SAXS). Troponin C (TnC) from chicken was one of the first myofilament proteins whose structure was determined in atomistic detail, initially via crystallography in 1988 (Ka et al., 1988) and later by NMR (Dvoretsky et al., 2002). Structures of the troponin complex, including the complete TnC molecule with fragments of TnI and TnT were more recently resolved via X-ray (PDB: 1J1E) in 2003 (Takeda et al., 2003). Macromolecular structures of intact filaments or the Z-disk are less common, but have relied on techniques including cryo-EM spectroscopy, SAXS, and computational protein/protein docking techniques (Alamo et al., 2016; Yamada et al., 2020; Wang et al., 2021). As an example, a reconstruction of the thin filament was obtained by docking proteins like troponin and tropomyosin to actin filaments, using data collected from cryo-EM (Yamada et al., 2020). Similar approaches were also used for the thick filament (Alamo et al., 2008) and the Z-disk (Wang et al., 2021). Despite the strengths of these methods in determining the Angstrom-resolution structures of many well-folded myofilament proteins, at most limited details of IDRs are revealed through these approaches. The paucity of IDR information in these structural models therefore leaves a large gap for linking structure to function.

Computational approaches have grown in tandem with experimental techniques to utilize and inform structure determination studies of myofilament proteins. Computer simulations of protein structure, properties, and functions have long served and will continue to play vital roles in elucidating myofilament mechanics and regulation. Early studies relied on descriptions of proteins as static bodies that could interact through steric and long-range interactions (Millman and Irving, 1988). For instance, myofilaments have been described as charged rods with negative electrostatic potentials, from which electrostatic fields within the myofibril could be predicted (Millman and Irving, 1988). Dynamic models of myofilament proteins have largely consisted of molecular dynamics (MD) simulations, coarse-grained simulations, and implicit representations (Kekenes-Huskey et al., 2013; Lindert et al., 2015; Aboelkassem et al., 2019), which have been made possible through the availability of hundreds of experimentally determined protein structures. These simulations have provided critical insights into the molecular mechanisms underlying sarcomere contraction, its modulation by PTMs, and impacts of missense variants on contractile function. Bosswman and Lindert (2019) offer an excellent review of such applications applied to folded myofilament proteins.

#### Myofilament-associated protein with intrinsic disorder (MAPID)s

**Fig. 3.**
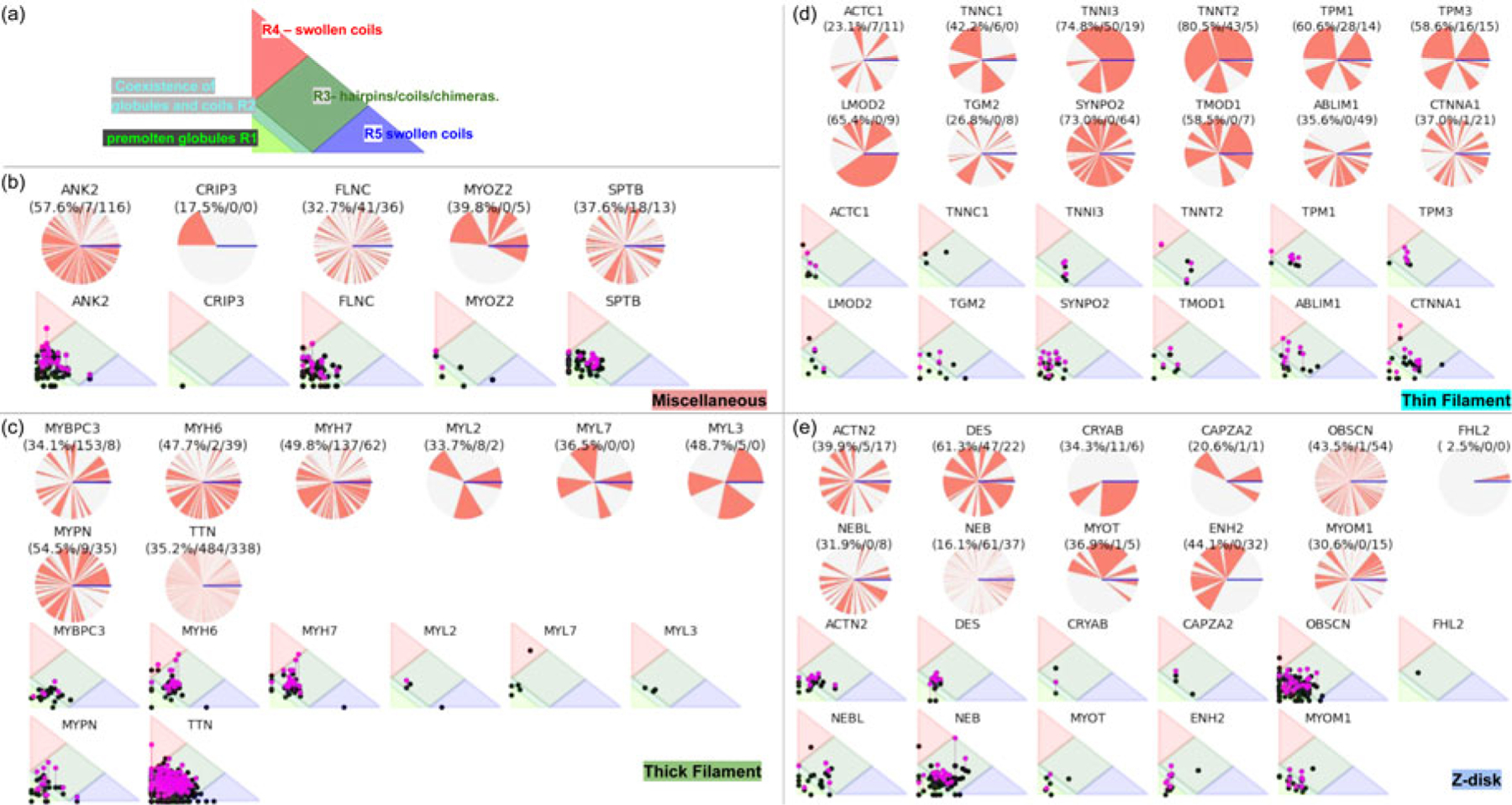
(*a*) The IDP-phase diagram developed by the Pappu lab (Holehouse *et al*., 2017), which groups proteins by characteristic disorder including molten, extended, or compact (Uversky, 2020) classes have also been proposed based on their charge patterns (R1–R5) (Holehouse *et al*., 2017): R1 corresponds to weak polyampholytes and resembles pre-molten globules. R3 signifies strong polyampholytes with a comparable amount of positively and negatively charged residues and is described as hairpins/coils/chimeras. R2 is the boundary between R1 and R3 where coils and pre-molten globules coexist. R4 and R5 are strong polyampholytes like R3, but with dominant negative and positive residues, respectively. IDPs in R4 and R5 are swollen coils. (*b*–*e*) PONDR-VLXT predicted disordered regions in cardiac myofilament proteins. These proteins are categorized into thin/thick filament(s), Z-disk, and miscellaneous. The IDP region is colored red and interlaced with folded regions. The blue line depicts the first and last amino acid and the number is increasing counterclockwise. The numbers in the parentheses present the percentage of predicted IDR residues, ‘pathogenic or likely pathogenic’ mutations, and phosphorylation sites located in the predicted IDP regions, respectively. The structural state estimation of predicted >5 residue IDR regions before (black dots) and after phosphorylation (magenta dots, if PTM site exists in the IDP region) in the IDP-phase diagram were also shown.

Despite advances made with well-folded, globular proteins, a substantial fraction of the myofilament lacks well-folded structure. Proteins lacking folded structures are referred to as proteins with IDRs when partially folded, or as an IDP if the protein is mostly or completely unfolded. We describe such myofilament proteins as MAPIDs to distinguish them from their well-folded myofilament counterparts. MAPIDs play pivotal roles in myofilament function. One such example is the troponin complex consisting of TnI, TnC, and TnT proteins that triggers muscle contraction after binding Ca^2+^ (Metskas and Rhoades, 2016). Both TnI and TnT have IDRs that are involved in initiating contraction (Hoffman and Sykes, 2008; Hwang et al., 2014; Johnston et al., 2019). Another example is the behemoth titin protein, which features numerous proline, glutamate, valine and lysine-rich (PEVK, ∼28 residue IDR (Linke et al., 1998; Ma and Wang, 2003)) repeats that help maintain passive tension in myocytes (Yamasaki et al., 2001). In fact, IDRs appear to be very common among the proteins of the myofilament. To estimate their propensity, we used the PONDR software ‘PONDR-VLXT’ (Li et al., 1999; Romero et al., 2001) to predict IDRs in the sequences of myofilament proteins listed in Fig. 3. PONDR identified that among over 30 myofilament genes that we considered in this work, approximately 42% of the amino acids in the sequences have potential disorder. This number resembles estimates of 30–50% for the entire Eukaryotic proteome (Best, 2017; Clarke and Pappu, 2017).

In this review, we discuss predominant myofilament proteins identified in the adult rat cardiac myofilament by Kooij et al. (2014) and several additional genes of recent interest including ENH2, MYOT, NEB, MYPN, and LMOD2. We limit our discussions to the major isoforms of these proteins. For those with multiple isoforms, we select the most common based on Kooij *et al*..^2^ We classify these proteins further using the classification scheme introduced in section ‘Molecular function of myofilament proteins’ and Fig. 1 which defines three regimes: the thin filament, the thick filament, and the Z-disk. For convenience, proteins that are localized to two more regimes, such as MyBPC3 linking the thin and thick filaments, are assigned to a single class. For each gene, we briefly introduce available IDR studies for it, where applicable. Experimental approaches used for the IDR studies are described in part 2. If IDR studies are not reported, we use two sequence-based approaches, the PONDR IDR predictor (Obradovic et al., 2003) and IDR state-diagrams (Das and Pappu, 2013; Das et al., 2015; Holehouse et al., 2017) in Fig. 3 to estimate IDR propensity and structure. We also refer to the ClinVar (Landrum et al., 2013) and PhosphoSitePlus (Hornbeck et al., 2015) databases for single nucleotide polymorphisms and PTMs within IDRs, respectively.

##### MAPIDs of the thin filament.

We first describe the thin filament proteins, which we divide into two groups: those forming the core of the thin filament and those associated with the thin filament. The core proteins of the thin filament include actin, troponin (TnC, troponin I (TnI), troponin T (TnT)), and Tm. The structures of many of these proteins have been determined down to Angstrom-level resolution, though many of these include IDRs that have not been completely resolved. The core structure of the intact thin filament comprising the troponin complex, actin, and tropomyosin has been resolved by cryo-EM (Yamada et al., 2020) (see Fig. 2).

##### Core thin filament MAPIDs.

*Actin (ACTC1)* Actin is a 42 kD protein that forms the backbone of the thin filament (Despond and Dawson, 2018; Frank et al., 2019). In the thin filament, actin has a well-folded structure when co-assembled with troponin and tropomyosin (PDB 6KN8 (Yamada et al., 2020)). Actin-binding proteins participate in actin filament formation (Miao et al., 2018). However, experimental evidence suggests that actin does not fold spontaneously without ligand binding or chaperones (Neirynck et al., 2006; Turoverov et al., 2010). This agrees with bioinformatics studies that suggest actin contains a significant degree of IDR content (Turoverov et al., 2010; Povarova et al., 2014). Within these IDR regions, we identified 42 phosphorylation sites and two disease-associated mutations using the PhosphoSitePlus and ClinVar databases, respectively.

*Troponin C (TNNC1)* The Tn complex comprises troponin C (TnC), troponin I (TnI), and troponin T (TnT). This complex serves as the central hub on the thin filament that transduces Ca^2+^ binding to priming the thin filament for myosin binding and ultimately contraction (Marston and Zamora, 2020). Troponin C (18 kD) has been extensively studied in isolation or in the intact Tn macromolecule with TnI and TnT (Hoffman et al., 2006; Hoffman and Sykes, 2008; Lindert et al., 2012a, 2012b; Zamora et al., 2016; Marques et al., 2019). The Ca^2+^ sensor TnC is one of the seemingly few myofilament proteins for which a complete, well-folded structure has been resolved. One of the complete structures for TnC was crystallized as a complex of TnC, TnI, and TnT via X-ray crystallography at 3.3 Å resolution (PDB code:1J1E (Takeda et al., 2003)). Nonetheless, the linker bridging its N- and C-terminal domains is predicted to be intrinsically disordered (Na et al., 2016), while predictions using PONDR in Fig. 3 suggest even greater propensity for disorder. We discuss this in greater detail in Fig. S1. Coincidentally, it has few PTMs (4 from PhosphoSitePlus database (Hornbeck et al., 2015)) and 10 likely pathogenic variants from the ClinVar database (Landrum et al., 2013).

*Troponin I (TNNI3)* TnI is a 24 kD protein that binds to a hydrophobic patch on TnC that is exposed following Ca^2+^ binding (Marston and Zamora, 2020). TnI binding to TnC primes the thin filament for myosin/actin cross-bridge formation (Marston and Zamora, 2020). TnI is perhaps the best studied of the IDR-containing proteins that form intact Tn. TnI’s N-terminal fragment, which consists of residues M1–H34, is intrinsically disordered and is not represented in troponin crystal structures from Takeda et al. (2003). The mobility of the disordered region in the TnI’s N-terminal domain is integral to its function (Hoffman et al., 2006). Using solution NMR spectroscopy, Hwang *et al*. revealed that this region plays an important role in positioning troponin C for its function (Hwang et al., 2014) and its conformational fluctuations impact Ca^2+^-regulated myosin binding to the thin filament (Hoffman et al., 2006; Hoffman and Sykes, 2008). This IDR also harbors three PTM sites (S5/S23/24) and possible cardiac disease-related mutations (Hwang et al., 2014; Metskas and Rhoades, 2016; Na et al., 2016). In addition, Takeda *et al*. suggested that TnI’s residues E66–R79 are likely disordered because this region folded only when interacting with troponin C (Takeda et al., 2003). Residues G137–R146 that form its inhibitory peptide are also unresolved (Takeda et al., 2003). This inhibitory peptide binds to TnC’s hydrophobic patch, which is a process that has been the subject of many computational studies in recent years (Lindert et al., 2012b, 2015; Bowman and Lindert, 2019). Lastly, the TnI C-terminus (residues I125–S210) is also predicted to be an IDR (Hoffman and Sykes, 2008) and contains sites for PTMs. For instance, a mouse model examining the phosphorylation of S199, which resides in the C-terminal IDR, was found to impair diastolic cardiac function by increasing Ca^2+^ sensitivity (Li et al., 2017). Nearby, an acetylation mimetic at K132 exhibited accelerated relaxation relative to the native amino acid (Lin et al., 2020). TnI also presents 12 disease-associated variants as reported by the ClinVar database and tens of likely pathogenic mutations associated with cardiomyopathy (Lu et al., 2013).

*Troponin T (TNNT2)* TnT is a 36 kD protein that regulates muscle contraction by binding to tropomyosin following Ca^2+^-activation of TnC/TnI (Marston and Zamora, 2020). TnT has two IDR regions. The first is a ∼50 residue linker (approximately residues R158–Q203) between two structured motifs (Deranek et al., 2022). Cross-linking mass spectroscopy (MS) shows that the binding of TnT’s intrinsically disordered C-terminus to TnC contributes to force generation in the myofilament (Johnston et al., 2019). Using Foerster resonance energy transfer (FRET) and molecular dynamics (MD) simulations, the linker’s conformational ensembles on the full cardiac thin filament have been elucidated (Deranek et al., 2022). The second region is the C-terminus of TnT, which has been predicted to be an IDR (Na et al., 2016), and confirmed by our PONDR results in Fig. 3. PhosphoSitePlus indicates that the C-terminus harbors several PTM sites (T213, S249, Y251, and T294). Phosphorylation of several of these sites is reported to alter cardiac contractility by either reducing Ca^2+^ sensitivity or ATPase activity (Streng et al., 2013). Similar to TnI, TnT hosts tens of mutations that are linked to cardiomyopathy (Lu et al., 2013).

*Tropomyosin (TPM1 and TPM3)* The 33 kD tropomyosin isoforms engage TnT to activate the thin filament. Modeling studies to date have targeted the well-folded helices that shift (Rynkiewicz et al., 2015) along the actin filament (Lehman, 2016) to unveil myosin-binding sites. Tm forms a flexible coiled-coil structure that binds to the thin filament (Singh and Hitchcock-DeGregori, 2003; Yamada et al., 2020). This flexibility is a key modulator of TM’s function, as mutations of a highly conserved residue D137L, and a dilated cardiomyopathy (DCM) mutation D230N in α-tropomyosin (TPM), causes a structural rearrangement of its coiled-coil structure that consequently alters its flexibility (Yar et al., 2013; Lynn et al., 2017). These changes ultimately impair tropomyosin (TM) function (Yar et al., 2013; Lynn et al., 2017). Its N-terminal domain is confirmed to be an IDR by NMR and circular dichroism (CD) studies, which show that this domain gains helical content upon binding to tropomodulin (Kostyukova et al., 2007). This IDR character is believed to explain the inability to resolve the region via cryo-EM in an earlier study (Milligan et al., 1990). Two PTM sites (S6/S16 for TPM1 and T5/T6 for TPM3) reside within the N-terminal extension as inferred from PhosphoSitePlus (Hornbeck et al., 2015). The ClinVar database also indicates several possible pathogenic variants in the N-terminal IDR (K30Δ for TPM1; E3Q, D15N, and M9R for TPM3). In addition, a K15N mutation in TPM1 is reported to change actin’s slow-growing (pointed) end dynamics (Colpan et al., 2017), which may impact sarcomere assembly.

##### Thin filament associated MAPIDs.

*Leiomodin (LMOD2)* LMOD2 is a 62 kD protein that helps lengthen the thin filament by driving actin assembly at the filaments’ barbed ends (Pappas et al., 2015). While three leiomodin isoforms are known, LMOD2 has the highest expression level in cardiac tissue (Tolkatchev et al., 2021). A linker connecting three actin binding sites in LMOD2 are likely to be intrinsically disordered, since (1) it is enriched in negatively charged residues and (2) was not resolved in its crystal structure (Tolkatchev et al., 2021). Immunofluorescence imaging and co-sedimentation experiments showed that this potential IDR facilitates the binding of LMOD2 to the thin filament by displacing bound tropomodulin (Tsukada et al., 2010; Colpan et al., 2017; Tolkatchev et al., 2021). Three possible pathogenic variants within this IDR region are reported in ClinVar (R513Ter, L415fs, and W398Ter) in addition to PTMs at sites Y369, T384, Y390, T409, S412, S416, T420, and T456 in the PhosphoSitePlus database.

*Tropomodulin-1 (TMOD1)* Tropomodulin (40 kD) is an actin-binding protein that belongs to the same protein family as leiomodin (Tolkatchev et al., 2021). TMOD1 regulates actin filament assembly (Boczkowska et al., 2015) and requires tropomyosin (Tm) for its regulatory functions (Kostyukova et al., 2007). The N-terminus of tropomodulin is an IDR based on its susceptibility to proteolysis (Kostyukova et al., 2000) and CD spectroscopy (Kostyukova et al., 2007). The region assumes an alpha-helical configuration, however, when bound to the tropomodulin IDR (Kostyukova et al., 2007). This IDR’s dynamic equilibrium exhibits ‘avidity’, in that it favors multiple binding interactions with tropomyosin and actin (Tolkatchev et al., 2021). The dynamics of the complex binding arrangement was recently examined in a multiscale modeling strategy entailing docking and MD refinement (Tolkatchev et al., 2021). Putative PTM sites are identified at S2, Y3, Y10, and T23 from PhosphoSitePlus (Hornbeck et al., 2015) that may suggest regulatory control of thin filament assembly. Several mutations (A21K/E33V (Moroz et al., 2013) and T54E (Dorovkov et al., 2008)) in the N-terminal IDR have also been characterized. These mutations are shown to alter TMOD1’s binding affinity toward tropomyosin (Moroz et al., 2013) and abolish TMOD1’s actin capping function (Dorovkov et al., 2008). As of yet, no variants of this gene have been reported in ClinVar.

*Catenin Alpha 1 (CTNNA1)* is a 100 kD mechanosensitive protein that couples the actin cytoskeleton with cadherins of the cell membrane (Vite et al., 2015). CD spectroscopy indicates that its helix bundle E (residues Q260–R360) is intrinsically disordered in the free protein (Hirano et al., 2018). This unfolding facilitates binding to vinculin (Hirano et al., 2018). Eight PTM sites in the helix E were reported in the PhosphoSitePlus database, in addition to two possible pathogenic variants (E307K and L318S) in ClinVar.

*Transglutaminase (TGM2)* is a 77 kD protein that catalyzes covalent bonding of glutamine and lysine side chains (Lorand and Graham, 2003). In the heart, it is implicated in cardiomyocyte development and signaling (Sane et al., 2007). The enzyme may localize to the thick filament, based on observations of its co-localization with the A-band in cultured, embryonic chicken myoblasts (Kang et al., 1995). TGM2 possesses several IDRs spanning the entire protein as suggested by missing regions within its crystal structures (Pinkas et al., 2007; Kanchan et al., 2015). Bioinformatics studies by Thangaraju *et al*. indicate that human TGM2 has more IDRs forming short linear motifs (SLIMs) than in other species (Thangaraju et al., 2017). These SLIMs are important as they enable TGM2 to interact with multiple protein partners, which contributes to its multi-faceted functionality in human (Thangaraju et al., 2017). PTM sites within potentially disordered loops have been reported in the PhosphoSitePlus database and include Y245, S250, T368, Y369, S415, and S4192. To our knowledge, disease-associated mutations in TGM2 have not yet been reported.

*Synaptopodin 2 (SYNPO2)* (aka myopodin) is an 118 kD protein involved in actin assembly during myofibril development (Linnemann et al., 2013), where it stimulates actin polymerization and aggregation (Chalovich and Schroeter, 2010). Synaptopodin 2 shares a high sequence identity of about 70% with fesselin. The latter protein has limited secondary structure as measured by CD and its large Stokes radius, which suggest that fesselin, and potentially synaptopodin 2 given its sequence similarity, are unfolded in their native state (Khaymina et al., 2007). Almost 100 PTM sites have been reported for SYNPO2 in PhosphoSitePlus. While we did not identify SYNPO2 mutations in its IDR that are attributed to cardiomyopathy, it has been reported that the reduced expression of SYNPO2 destabilizes myofibrils (Lohanadan et al., 2021).

*Actin-binding LIM protein 1 (ABLIM1)* is an 88 kD protein that traverses the actin cytoskeleton (Roof et al., 1997) and links the Z-disk binding domains of titin; the latter engagement is believed to help regulate length-dependent activation of cardiomyocytes (Stachowski-Doll et al., 2022). Although studies of the mammalian ABLIM1 gene’s IDRs have not been reported in the literature, the actin-binding plant LIM protein has been experimentally confirmed to have an IDR linker connecting its two LIM domains (Ma and Miao, 2020). Interestingly, this IDR mediates self-aggregation of plant ABLIM proteins and thereby shapes F-actin remodeling in plants (Ma and Miao, 2020). By extension, the homologous region in the cardiac ABLIM1 may also be disordered. We base this suggestion on our PONDR prediction of ABLIM1, which indicates that the residues flanking the first and fourth LIM domains, as well as the C-terminal fragments, are IDRs (Fig. 3). Within these putative IDRs, 92 PTM sites are identified in the PhosphoSitePlus, although no variants have yet been reported in the ClinVar database.

##### MAPIDs of the thick filament.

The thick filament generates force when the thin filament is activated. Myosin is the predominant constituent of the thick filament, while titin and MyBPC3 link the thick filament to the Z-disk (LeWinter and Granzier, 2010) and thin filament (Flashman et al., 2004), respectively. It is now appreciated that most hypertrophic cardiomyopathy (HCM)-causing mutations are found in thick filament genes MYH7 and MyBPC3 (Xu et al., 2010; Harris et al., 2011), although the thin filament troponin complex presents additional HCM variants (Willott et al., 2010). DCM mutations have also been identified in MYH7 (Xu et al., 2010), MyBPC3 (Xu et al., 2010), and TTN (Harris et al., 2011).

*Myosin (MYH6-7 and MYL2,3,7)* The cardiac myosins belong to a super family containing numerous isoforms (Hartman and Spudich, 2012). The myosin isoforms comprising the heavy (MYH6 and MYH7) and light (MYL2, MYL3, and MYL7) chain are the work-horses of myofilament contraction, leveraging the hydrolysis of bound ATP to ratchet along exposed actin-binding sites (Spudich et al., 1995). The heavy chains are approximately 220 kD while the light chain isoforms are significantly smaller at 20 kD. Our PONDR results in Fig. 3 indicate that there are about 30 putative IDR regions in MYH6/MYH7, and about five in MYL2/MYL3/MYL7. Roughly 130 HCM mutations and 30 DCM mutations are reported in myosin, with most in the MYH7 gene (Carniel et al., 2005; Alamo et al., 2017; Kim et al., 2020). Consistent with those reports, the ClinVar database reports 346 pathogenic and likely pathogenic variants for these five myosin genes. Of these, 152 reside within its potential IDRs (Fig. 3). The cardiac myosins are also prime targets for PTMs and especially phosphorylation. According to PhosphoSitePlus, nearly 103 of these PTMs fall within potential IDRs for the cardiac myosin genes (Fig. 3). This abundance of PTM sites likely helps regulate thick filament assembly and contraction (Pfitzer, 2001). In support of this, it has been reported that blunting myosin light chain 2 phosphorylation leads to abnormal cardiac structure and function in mice (Sanbe et al., 1999).

MYH7 is perhaps the most well-studied of the two heavy chains, including several computational studies, as it is the predominant isoform in the human heart (Kelly et al., 2018). A structural model of the human MYH7 complex, including its myosin light chains, was built from tarantula skeletal muscle thick filaments (Nag et al., 2017). The interactions between protein domains were verified via biochemical assays. Based on this model, some HCM mutations are shown to destabilize the myosin complex, which may explain their detrimental effects on cardiac function (Nag et al., 2017).

One study of note aimed to infer the phenotype for VUSs in well-folded regions of myosin (Toepfer et al., 2020). This study indicated that known pathogenic variants disturb myosin’s functional conformation dynamics through altering the myosin head domain’s interactions. The proximity of five myosin VUSs to these pathogenic variants was found to correlate with clinical phenotypes; namely, VUS located close to the head domain had more severe clinical outcomes (Toepfer et al., 2020). It would be interesting to determine if VUS within IDR regions near the head domain had similar impacts on myosin function. In addition, HCM mutations are reported in the S2 motif (R870H, E924K, and E930Δ), which were shown to reduce myosin binding to MyBPC3 (Singh et al., 2021). These mutations uncouple the coiled-coil structure upon addition of denaturant as evidenced by CD. These findings raise the possibility that other variants may induce disorder that disrupts myosin function. Adjacent to the S2 motif, the light meromyosin (LMM) region of myosin also harbors disease mutations. Parker et al. (2018) combined experimental assays and MD simulations to show that two disease mutations, A1603P and K1617Δ, in the LMM motif reduce coiled-coil helicity and lead to abnormal sarcomere assembly. Large-scale gene sequencing has additionally identified mutations in MYH6 and MYH7 that are associated with congenital heart disease (Jin et al., 2017; Theis et al., 2021; Yu et al., 2021).

The myosin heavy chains contain several IDRs, which play important roles in binding to actin (Robert-Paganin et al., 2020). These IDRs have been more extensively studied in both cardiac and non-muscle myosin isoforms (Risi et al., 2017; Doran et al., 2020). Risi *et al*. constructed a structural model of the cardiac actomyosin complex by fitting available high-resolution myosin/thin-filament structures (von der Ecken et al., 2016) to the cryo-EM density of the cardiac thin filament (Risi et al., 2017). This model shows key structural motifs of the myosin, such as the highly dynamic loop 4, which has direct contact with the thin filament. More importantly, this cardiac model shows that tropomyosin assumes a different angle compared with that in skeletal model, which may explain the higher activation potential of cardiac filament by Ca^2+^ (Risi et al., 2017). Similarly, a state model of the skeletal myosin/F-actin/tropomyosin complex was built via a combination of docking/cryo-EM fitting and MD simulations (Doran et al., 2020). This model provides atomic-level resolution for the myosin motor functional cycle and shows that the interactions between myosin’s dynamics loop 4 (amino acids 354–380) and the thin filament are crucial for myosin motor activation (Doran et al., 2020).

A complex between myosin VI (MYO6) with F-actin at 4.6 Å via cryo-EM spectroscopy and MD simulations was reported (Gurel et al., 2017) that could yield mechanistic clues for cardiac myosin. Specifically, the study suggested that two disordered loops form essential interactions with actin that stabilize the complex, but were not resolved (Gurel et al., 2017). Importantly, one of these loops (T392–P410) is homologous with cardiac myosins and is a locus for several HCM-causing mutations (Gurel et al., 2017). Based on studies in scallop striated muscle myosin, a helix motif (C693–F707) undergoes a disorder-to-ordered transition during its functional cycle (Houdusse et al., 1999). The human cardiac myosin (MYH7) likely exhibits the same transition given that the motif is conserved. Similarly, the α-helical coiled-coil structure of Myosin Va (MYO5a) only folds upon binding to the myosin Va light chain (Wagner et al., 2006), which may also occur in cardiac myosin. Along these lines, MD simulations have shown that the N-terminal IDR of smooth muscle myosin regulatory light chain undergoes a disorder-to-ordered transition upon phosphorylation (Espinoza-Fonseca et al., 2007), highlighting the importance of PTMs in regulating myosin function.

Atomistic structures of the myosin regulatory light chains (MYL2 and MYL3) in complex with the cardiac myosin heavy chain (MYH7) have been obtained via homology modeling (PDB 5TBY (Alamo et al., 2017), Fig. 2). While the MYL2 structure is mostly complete with only a few unresolved N-terminal residues, the MYL3 structrure is missing nearly 40 N-terminal residues (Alamo et al., 2017), which suggests that the region is intrinsically disordered. To our knowledge, IDR studies of the myosin light chain isoforms have not been reported.

*Cardiac myosin-binding protein C (MyBPC3)* is a 137 kD protein that bridges the thin and thick filament (Oakley et al., 2004). It is generally thought to simultaneously modulate myosin availability to bind actin as well as the availability of myosin-binding sites on actin (Heling et al., 2020). In this capacity, its chief interaction partners are myosin, actin, and titin (Oakley et al., 2004). Details continue to emerge, but there is a growing appreciation that MyBPC3 maintains the thick filament ‘off states’ and thin filament ‘on states’ (Kampourakis et al., 2014) that prevail during diastole and systole, respectively. At low Ca^2+^, MyBPC3 may also sequester myosin heads in a super-relaxed state, which describes a shift of their conformational ensemble from actin to the myosin tails of the thick filament (Palmer et al., 2011). Using skinned myocardial strips experiments, Tanner *et al*. showed that phosphorylation of cardiac MyBPC3 accelerates the rates of myosin detachment from thin filament (Tanner et al., 2021), suggesting MyBPC3 plays a key role in regulating myofilament force generation. The abundance of HCM-causing mutations identified on MyBPC3 (Harris et al., 2011), including 153 pathogenic or likely pathogenic variants located in its predicted IDRs (Fig. 3), have been the topic of studies using both experimental and computational methodologies (reviewed in Sequeira et al., 2014; Main et al., 2020).

Three MyBPC3 isoforms are found in muscle: fast skeletal, slow skeletal, and cardiac. Although all three isoforms share a common domain organization consisting of seven immunoglobulin I-like (Ig) domains and three fibronectin 3-like domains (Flashman et al., 2004), the cardiac isoform has several additional motifs that are indispensable for its function (Howarth et al., 2012). For example, MyBPC3’s ∼270 residue N-terminus comprising the C0–C1 domains is crucial for heart function, as deletion of these residues resulted in DCM in a mouse model (Lynch et al., 2021). The modular nature of MyBPC3 likely has the advantage that it allows the protein’s function to be tuned by modifying its domain properties. Napierski *et al*. for instance demonstrate that an MyBPC3 construct lacking the C0–C7 domains exhibits abnormal function, which is rescued by inserting recombinant C0–C7 domains (Napierski et al., 2020). Another unique motif in cardiac MyBPC3 is the ∼100-amino acid M-domain that bridges its C1 and C2 domains (Howarth et al., 2012) and binds myosin (Singh et al., 2021). The N-terminal fragment of the M-domain was identified as an IDR by CD and NMR (Howarth et al., 2012). Atomic force microscopy (AFM) studies additionally indicate that its phosphorylation reduces the M-domain’s extensibility and likely attenuates MyBPC3’s ability to regulate cardiac muscle contraction (Previs et al., 2016). Intriguingly, NMR studies have also identified a highly flexible linker in the M-domain that serves as a major binding site for the regulatory calmodulin (CaM) (Michie et al., 2016), which we speculate may confer Ca^2+^-dependent conformational changes to its IDR. Additionally, Colson et al. (2016) used MD simulations to demonstrate that M-domain (residues T255–R357) phosphorylation reduced its structural disorder, stabilized the C0/C1 domain folds, and exposed a cryptic protein–protein binding site. This finding provides mechanistic insight into how regulatory control via the PTMs within IDRs in the M-domain impacts cardiac contractility.

In addition to the M-domain, other IDRs in MyBPC3 are speculated to exist. AFM analyses from Karsai *et al*. indicate that the force–extension relationship of the intact MyBPC3 is heterogeneous, with some regions extending more easily than would be expected for folded Ig domains (Karsai et al., 2011). These regions are believed to be disordered and likely correspond to the linkers connecting MyBPC3’s folded domains. Doh et al. (2022) showed that the flexible linker connecting the C4 and C5 domains of MyBPC3 not only modulates the secondary structure content in the C4 and C5 domains, but also affects their relative interdomain orientations and associated kinetics (Doh et al., 2022). By using MD simulations, they pinpointed specific residue–residue contacts, and the local conformation changes of the linker, that contribute to its modulatory role. Another study focusing on the interdomain flexible linker from Potrzebowski et al. (2018) combined Bayes inference and molecular simulations to build structural models of an MyBPC3 construct including an M-domain fragment, C2-domain, and a linker. The structural models that best match the experimental SAXS data show diverse interdomain orientations, demonstrating the flexible linker’s role in supporting a broad conformation ensemble. Interestingly, genome scale bioinformatic analysis has also revealed that MyBPC3 splice isoforms tend to overlap with disordered regions (Lau et al., 2019), which implicates the IDRs in myofilament function.

*Titin (TTN)* is a behemoth protein (ca. 3816 kD) that secures the thick filament to the Z-disk, by spanning one-half sarcomere to the M-line (LeWinter and Granzier, 2010). Titin is highly modular in that it contains folded Ig domains interspersed with unstructured regions such as PEVK motifs (Linke et al., 1998). The PEVK repeats are intrinsically disordered, ∼28 residue motifs enriched in proline, glutamic acid, valine, and lysine (Ma and Wang, 2003). These IDRs contribute to titin’s passive elasticity (Linke et al., 2002; Ma and Wang, 2003) and are substrates for proteins like S100A1 (Yamasaki et al., 2001). It has been speculated that S100A1 binding at these repeats modulates passive tension (Granzier et al., 2010). The PEVK repeat was observed to have mostly disordered secondary structure (Poly II helix, b-turn and coils) and larger Stokes radius using CD, gel permeation chromatography, and gel electrophoresis, which confirm its intrinsically disordered nature (Ma and Wang, 2003; Duan et al., 2006). Interestingly, idiopathic restrictive cardiomyopathy mutations have been found in the PEVK motifs as well as the fibronectin-type III (FnIII) domains, of which the latter are also likely to be disordered (Tarnovskaya et al., 2017). Mutations in these regions were predicted via PONDR-FIT (Xue et al., 2010) to alter their disorder (Tarnovskaya et al., 2017).

Beyond titin’s PEVK motifs, linkers connecting its modular domains are likely IDRs based on predictions from PONDR (Fig. 3). Simulations from the Schulten lab investigated the effect of the PEVK domains and domain unfolding on tension, which led to TTN’s characterization as an entropic spring (Lu et al., 1998; Lee et al., 2007). In addition, steered MD simulations have been extensively performed on TTN constructs consisting of multiple Ig domains and interdomain linkers (reviewed in Hsin et al., 2011). These simulations put forth a structural basis for TTN’s impressive plasticity, how interdomain bending is mostly mediated by flexible linkers, and how domain unfolding may influence plasticity. Moreover, IDRs in TTN could also serve as binding motifs for protein–protein interactions (PPIs). As an example, the N2A titin isoform contains long IDR linkers flanking its binding site for ankyrin repeat proteins that were determined to be intrinsically disordered using NMR and HDXMS (Zhou et al., 2021a). ClinVar and PhosphoSitePlus identify more than 400 pathogenic/likely pathogenic variants and over 300 PTMs within its predicted IDRs (Fig. 3). This concurs with suggestions that its defects are responsible for the majority of dilated cardiomyopathies (Herman et al., 2012).

##### MAPIDs of the Z-disk.

The Z-disk is a central hub (Sols and Solaro, 2021) that interfaces adjacent sarcomeres and sarcomeres to organelle membranes (see Fig. 1a). It is a nexus for sensing changes in mechanical demand and can communicate these changes to myriad signaling pathways to regulate cardiac function (Sols and Solaro, 2021). For this reason, it is also a prime target for a number of regulatory mechanisms (Sols and Solaro, 2021). Unlike the thin and thick filament for which the primary constituents have largely been identified, proteins composing and interacting with the Z-disk are continuing to be found.

α-*Actinin (ACTN2)* is a large (104 kD), dimeric protein that interfaces with titin and actin filaments, where it contributes to sarcomere assembly (Chopra et al., 2018). α-actinin cross-links actin filaments with the Z-disk (Maruyama and Ebashi, 1965) and also competes with calsarcin to bind calcineurin (CN) (Frey et al., 2000; Seto et al., 2013). α-actinin’s structure is almost entirely resolved at 3.5 Å resolution for residues Y19–L894 (PDB 4D1E (Ribeiro et al., 2014)). In its dimeric state, the structure does not present disordered structural domains. However, PONDR suggests α-actinin contains several IDRs, which perhaps may be evident in the isolated monomeric structure. Thirty-four PTMs have been reported by the PhosphoSitePlus, 17 of which are located in the predicted IDRs (Fig. 3). Fourteen pathogenic or likely pathogenic variants are reported in ClinVar, with five in the predicted IDRs.

*Crystallin alpha B (CRYAB)* CRYAB is a 20 kD, ubiquitously-expressed, small heat shock protein that regulates cellular responses to stress (Dimauro et al., 2018). As a chaperone, CRYAB associates with misfolded proteins to suppress their aggregation (Dimauro et al., 2018). At least under ischemic conditions, it binds with titin to potentially protect the protein from degradation (Golenhofen et al., 2002). The protein consists of three domains, the N-terminal domain (NTD, residues M1–S59), the α-crystallin domain (ACD, residues W60–K150), and the C-terminal domain (CTD, residues Q151–K175) (Braun et al., 2011). Although its complete structure is deposited at 9.40 Å resolution from cryo-EM as 24-meric alphaB-crystallin (Braun et al., 2011), higher-resolution NMR structures reveal only its ACD domain, while the NTD and CTD remain unresolved (Jehle et al., 2010). NMR experiments indicate that its C-terminus is intrinsically disordered and may self-aggregate (Baldwin et al., 2012); this aggregation is considered a pathological marker in histologies (Zhang et al., 2010). Three PTM sites S19, S45, and S59 have been identified in its N-domain (Chiappori et al., 2016). MD simulations reveal that phosphorylation of S59 alters a key interface for its multimeric self-assembly (Chiappori et al., 2016). Interestingly, three cataract-associated mutations were also found in this region, which were shown to both alter self-assembly and its interaction with other proteins (Muranova et al., 2020). In addition, nearly 10 other muscle disease-associated mutations have been reported in its ACD and CTD (Dimauro et al., 2018).

*Enigma homolog isoform 2 (ENH2)* is a 64 kD protein that interacts with calsarcin in the Z-disk and α-actinin (Cheng et al., 2010). As a splice isoform of the PDLIM5 gene (Huang et al., 2020a), ENH2 contains a postsynaptic density protein of 95 (PSD-95) PSD-95/Discs large/Zonula occludens-1 domains (PDZ) domain and three LIM domains. Of these, the PDZ domain plays important roles in signal transduction through PPIs formed with targets (Ivarsson, 2012). The LIM domain has two zinc fingers and also engages a diverse range of signaling pathways. The PDZ and LIM domains are generally folded (Elkins et al., 2010), which is consistent with our PONDR predictions of ENH2 in Fig. 3. The regions between the PDZ and LIM domains are predicted to be IDRs, which agrees with annotations from UniProt for EHN2. Emerging evidence also suggests that even the folded LIM domain likely contains IDRs, as an unpublished solution NMR structure of the LIM domain (PDB 2DAR) exhibits highly dynamic N- and C-termini.

Knock-out of the enigma homolog protein leads to impaired cardiac contraction and DCM in a mouse model (Cheng et al., 2010). Top–down proteomics, which utilizes mass spectrometry to characterize intact proteins, demonstrate that changes in ENH2 phosphorylation at S118 occur in ischemia (Peng et al., 2014) and HCM (Tucholski et al., 2020). PhosphoSitePlus suggests 32 PTM sites in the predicted IDR regions, while ClinVar does not report any pathogenic or likely pathogenic variants that change the protein sequence.

*Obscurin (OBSCN)* is an 869 kD protein that links myofibrils to the SR (Lange et al., 2009) and possibly to the cytoskeleton (Geisler et al., 2007). Similar to other modular proteins like titin and nebulin, obscurin consists of many folded domains joined by disordered linkers (Young et al., 2001). MD simulations have shown that the linkers between the modular domains of obscurin are flexible, which allows the bridged folded domains to sample broad conformational ensembles that likely contribute to the protein’s elasticity (Whitley et al., 2019). In addition, a solution NMR structure of obscurin’s PDZ domain (residues R3614–P3713, PDB 2EDH) is highly dynamic, which is consistent with PONDR predictions in Fig. 3. Of the putative PTMs found in PhosphoSitePlus (see Fig. 3), 54 are found within these IDRs. While ClinVar reports just one pathogenic or likely pathogenic variant that changes the sequence of the predicted IDRs, 16 cardiomyopathy-linked variants spanning the OBSCN sequence were reported in 2017 (Marston, 2017). Given the many intermittent IDR regions predicted in OBSCN (Fig. 3), these variants are likely to be located within or immediately adjacent to IDR regions. As OBSCN’s role in the sarcomere continues to be clarified, new cardiomyopathy-linked variants may continue to emerge (Marston, 2017).

*Myotilin (MYOT)* is a pseudonym for limb-girdle muscular dystrophy 1A (LGMD1A) protein (Salmikangas et al., 2003). The 55 kD protein localizes to the Z-disk and cross-links α-actinin, where it is believed to contribute to the assembly of actin filaments (Salmikangas et al., 2003). The N-terminal fragment and C-tail of myotilin are predicted to be disordered and contain binding sites for proteins such as α-actinin-2 and Z-disk-associated, alternatively spliced, PDZ motif-containing protein (ZASP) (Puž et al., 2017). This prediction is in excellent agreement with our PONDR scores for MYOT in Fig. 3. The presence of IDRs in MYOT is further confirmed by an unpublished solution NMR structure of myotilin’s C-terminal fragment (PDB 2KKQ) that shows highly dynamic regions. The IDR in the N-terminal fragment of MYOT harbors several muscle disorder associated mutations (Puž et al., 2017), and PhosphoSitePlus reveals five PTMs in its predicted IDRs.

*Myomesin 1 (MYOM1)* is a 188 kD protein that together with titin and obscurin form the dynamic ‘M’ band found between the Z-disks of the cardiac sarcomere (Lamber et al., 2022). In this arrangement, the protein plays an important role in sarcomere organization (Lamber et al., 2022). Myomesin 1 is also suggested to bind myosin (Lamber et al., 2022), which could directly impact sarcomere contraction. Predictions using IUPred2A (Mészáros et al., 2018) reveal that MYOM contains IDRs, while genome scale bioinformatic analysis suggests MYOM splice isoforms frequently overlap with IDRs (Lau et al., 2019). One of these splice isoforms contains an insertion of an ∼100 residue elastic segment (EH domain) at the center of the protein. This EH-myosin is the main component of the M-band in higher vertebrates (Schoenauer et al., 2011). Expression of the EH-myomesin is significantly up-regulated in DCM patients (Schoenauer et al., 2011). Moreover, AFM, transmission electron microscopy, and CD data suggest that the EH segment is disordered and contributes to the protein’s elasticity (Schoenauer et al., 2005). PONDR scores for MYOM1 in Fig. 3 reveal multiple IDRs spanning the protein. While PhosphoSitePlus reports 15 PTMs in the predicted IDRs, no variants that alter its IDR sequences are reported in Clinvar.

*Desmin (DES)* is a 54 kD protein that anchors the myofibrils by interconnecting the Z-disks to the cell cytoskeleton (Goldfarb et al., 1998; Brodehl et al., 2013). It serves both signaling and structural roles in cardiomyocytes (McLendon and Robbins, 2011). Mutations in desmin are associated with desmin-related (cardio)myopathy, which is also known as desminopathy (Goldfarb et al., 2008). These defects are accompanied by aggregates of misfolded proteins (McLendon and Robbins, 2011), which suggests potential correlations between desmin structural defects and protein quality control. Predictions using DISOPRED3 and DICHOT reveal that both the N- and C-terminus of desmin are intrinsically disordered (Anbo et al., 2019). This prediction is in agreement with our PONDR scores (Fig. 3). Moreover, the C-tail IDR harbors the binding site for the chaperone alpha B-crystallin (CRYAB) (Anbo et al., 2019). PhosphoSitePlus and ClinVar report 22 PTMs and 47 pathogenic or likely pathogenic variants, respectively, in the predicted IDRs.

*Four and a half LIM domains 2 (FHL2)* is a 32 kD protein that serves as a biochemical stress sensor in the sarcomere, in part through its interactions with titin (Sheikh et al., 2008). In principle it may also regulate phosphorylase activity given its binding interactions with CN phosphatase (Hojayev et al., 2012). According to several studies (Chu et al., 2000; Okamoto et al., 2013; Friedrich et al., 2014), the protein is believed to play a minimal role during normal cardiac development, but may limit the development of cardiac hypertrophy. Our PONDR scores for FHL2 show negligible IDR content, which is consistent with annotations from UniProtKB. However, solution NMR structures of its LIM domains are highly dynamic at their N/C-termini (PDB 2D8Z, 1X4L, 1X4K) and therefore may be characterized as IDRs. While no pathogenic/likely pathogenic variants that change the IDR sequence have been reported in the ClinVar database, PhosphoSitePlus reports five PTMs within the LIM termini.

*Nebulin (NEB) and nebulette (NEBL)*. Nebulette belongs to the nebulin family, but has a much smaller size of 116 kD compared with the 773 kD nebulin (Bang and Chen, 2015). Nebulin is primarily expressed in skeletal muscle while nebulette is highly expressed in cardiac muscle (Bang and Chen, 2015). Nebulin and nebulette are modular proteins that share a common building block called the ‘nebulin repeat module’ (Bang and Chen, 2015). Sequence comparisons between nebulette and nebulin from different species show that their basic building blocks are highly conserved (Moncman and Wang, 2000). Similar to nebulin (Labeit and Kolmerer, 1995), cardiac nebulette bridges actin to desmin (Hernandez et al., 2016) and regulates Z-disk assembly (Moncman and Wang, 1999). Studies of IDRs in nebulette have not yet been reported, therefore we refer to work focused on its homolog, nebulin.

Nebulin is a ∼1 μm long intrinsically disordered scaffolding protein along the thin filaments, with a significant percentage of its residues predicted as IDRs by the PONDR-VL3H algorithm (Wu et al., 2016). Its IDRs are suggested to regulate sarcomere assembly, given the close alignment between the periodicity of high disorder scores with the positions of myosin-associated proteins in the A-band (Wu et al., 2016). These disordered regions most likely reside between the nebulin building blocks. PTM sites have been identified in both nebulin and nebulette throughout their sequences, while most of those sites tend to localize to regions that share high homology (Moncman and Wang, 2000). Missense mutations of nebulin are mainly related to nemaline myopathy, while several mutations in nebulette are linked to familial and idiopathic dilated cardiomyopathy (Bang and Chen, 2015). Given the many intermittent IDRs in nebulin predicted by us (Fig. 3) and by Wu et al. (2016), it is likely that some of these disease-associated mutations fall within its IDRs. ClinVar reports 61 pathogenic/likely pathogenic variants in the predicted IDRs, in addition to 37 PTMs reported in the PhosphoSitePlus database (Fig. 3).

*Myopalladin (MYPN)* is a 145 kD protein that bridges nebulin (skeletal) or nebulette (cardiac) to α-actinin in the Z-disk (Bang et al., 2001). Myopalladin is a modular protein that contains five Ig-like domains (Bang et al., 2001); linkers connecting the modular Ig-like domains are likely to be IDRs based on the PONDR data in Fig. 3. Studies targeting these potential IDRs have not been reported in the literature. However, genetic screens of patients with cardiomyopathy have identified dozens of mutations in MYPN (Duboscq-Bidot et al., 2007; Purevjav et al., 2012), of which two fall within its IDRs. Several of these mutations cause disrupted Z-disk assembly along with abnormal co-expression of MYPN with α-actinin, desmin, and ankyrin (Purevjav et al., 2012). The PhosphoSitePlus reports 35 PTMs in the predicted IDRs.

*F-actin-capping protein subunit alpha-2 (CAPZA2)* is a 33 kD protein localized to the Z-disk that directs the orientation and direction of actin during muscle fiber development (Solis and Russell, 2019). It does so by regulating the growth of the barbed end of actin filaments (Funk et al., 2021). To date, variants in CAPZA2 have not been linked to cardiac disorders, although there have been associations established with mental impairment (Huang et al., 2020b). IDR-related studies have not been reported for CAPZA2, although based on Fig. 3, there is evidence of IDRs within residues M1–F20, D99–V124, and R259–W271. ClinVar and PhosphoSitePlus report two pathogenic/likely pathogenic variants (K256E and R259L) and one PTM (S4) located near the predicted IDRs.

##### Miscellaneous MAPIDs of the sarcomere.

Several proteins are associated with the myofilaments but are not unambiguously classified into the Z-disk, thick filament, or thin filament assemblies. Many contribute to sarcomere assembly, for instance through cross-linking proteins, or play important signaling roles, such as mechanosensing.

*B type ankyrin (ANK2)* is a 434 kD protein that bridges the sarcomere M-line to the cell membrane via obscurin (Cunha and Mohler, 2008), where it interacts with ion channels and transporters. Its dysfunction is linked to a variety of electrical defects in cardiac function (Sucharski et al., 2020). Its N-terminus contains well-folded 24 ankyrin repeats, which form a groove that can bind many IDR-containing membrane proteins (Wang et al., 2014). Its C-terminus is intrinsically disordered as evidenced by little to no secondary structure content in CD studies and its large Stokes radius (Abdi et al., 2006). The ankyrin repeats are auto-inhibited by several IDR fragments from its C-terminus, which was determined via binding assays and X-ray crystallography (Chen et al., 2017). Two idiopathic restrictive cardiomyopathy mutations are found in ankyrin’s IDRs (Tarnovskaya et al., 2017) in addition to about 80 PTM sites via PhosphoSitePlus.

*Cysteine-rich protein 3 (CRIP3)* is a 24 kD protein and together with CRIP1 and CRIP2, it forms the cysteine-rich intestinal protein (Crip) family (Hempel and Kuhl, 2014). CRIP3 and its homolog CRIP2 are expressed in cardiovascular tissue (Wei et al., 2011; Hempel and Kuhl, 2014), where they are speculated to have a role in mechanosensing (Boateng et al., 2007). Its precise localization in the sarcomere is not established, although the STRING database of PPIs (Szklarczyk et al., 2019) suggests it may associate with CAPZA3. CRIP3 is composed of two LIM domains connected by a ∼30 residue flexible linker. This linker is predicted to be an IDR by our PONDR scores (see Fig. 3). Mutations have been identified in the cysteine-rich protein family that are linked to dilated cardiomyopathy (Knöll et al., 2002). Two PTMs, Y132 and S139, located in the second LIM domain are reported in the PhosphoSitePlus database. No pathogenic or likely pathogenic variants in CRIP3 have been reported in ClinVar.

**Fig. 4.**
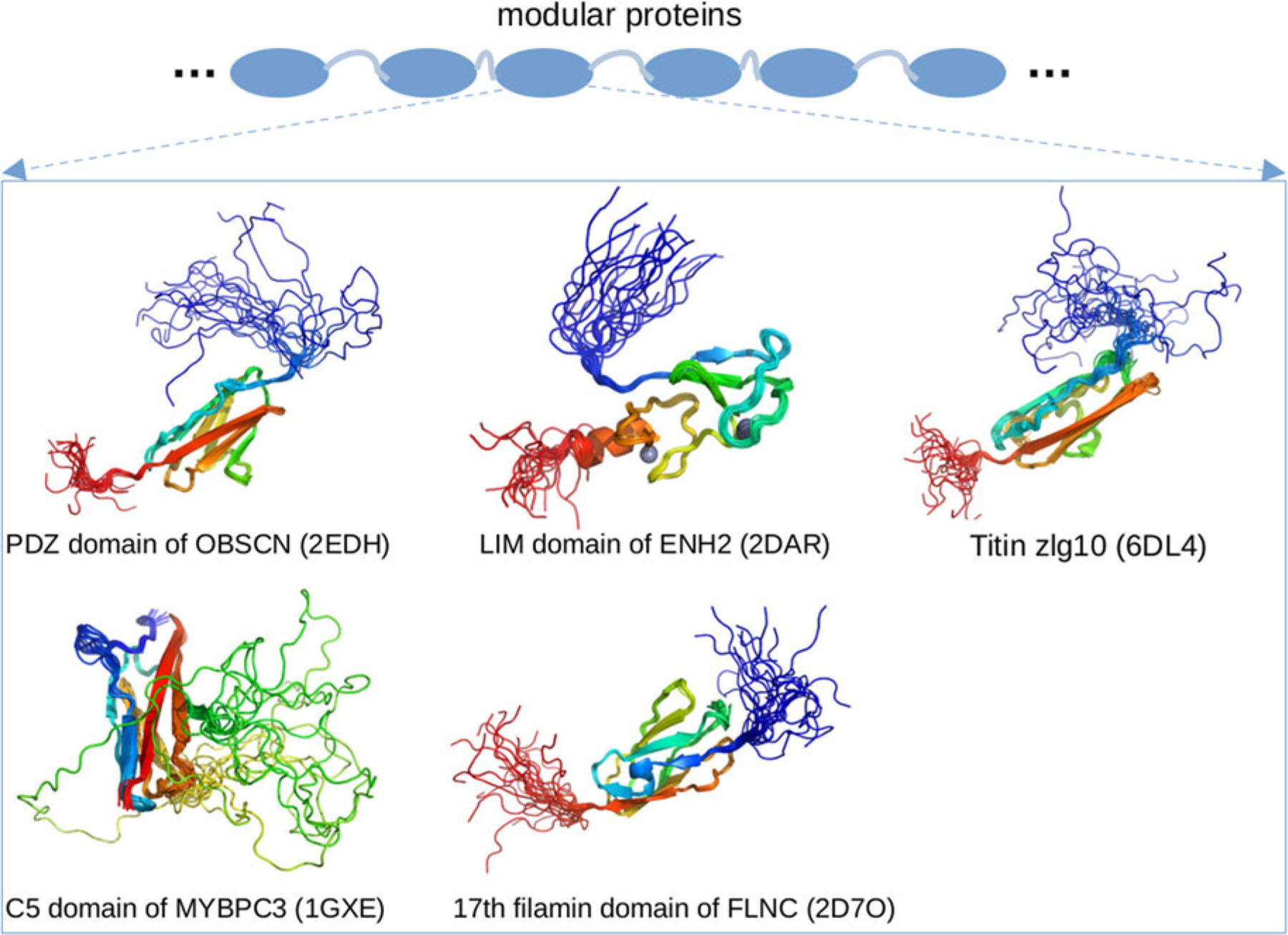
Solution NMR structures of the common modular domains that are used as building blocks of Z-disk proteins. These structures show highly dynamic termini and evidence of intrinsic disorder in the Z-disk. The PDB IDs are given in parentheses.

*Filamin-C (FLNC)* is a 291 kD protein that binds nebulette and ankyrin (Holmes and Moncman, 2008; Maiweilidan et al., 2011), where it contributes to sarcomere assembly (Agarwal et al., 2021). Filamin-C contains 24 folded repeats (Ig-like domains) in its C-terminus (Nakamura et al., 2011). These 24 repeats form two rod-like structures, with the first rod assuming an extended chain configuration and the second, a relatively compact folded configuration (Nakamura et al., 2011). These two rod configurations are necessary for filamin’s dimerization and binding to actin (Nakamura et al., 2011). Although IDR studies have not yet been reported for filamin-C, an unpublished solution NMR structure of its modular domain (Fig. 4, PDB 2D7O) shows highly dynamic N- and C-termini. PONDR scores for FLNC in Fig. 3 predict that its IDRs are interspersed in the protein’s sequence. Based on these predictions, 41 pathogenic or likely pathogenic variants and 36 PTMs are localized to the IDRs, based on the ClinVar and PhosphoSitePlus databases, respectively.

*Myozenin 2 (MYOZ2 or FATZ-2)* is a 39 kD Z-disk protein that binds to and inhibits CN, hence its pseudonym calsarcin (Frey et al., 2000; Ruggiero et al., 2012). While activation of CN in the adult heart is typically associated with cardiac hypertrophy (Ruggiero et al., 2012), mutations in myozenin 2 that are linked to cardiomyopathy and disorganization of the Z-disk may arise independently of CN activity (Ruggiero et al., 2012). Sponga *et al*. found that the MYOZ1 isoform, which shares around 36% sequence identity with MYOZ2, is intrinsically disordered (Sponga et al., 2021). This was rationalized by the MYOZ1 constructs having higher apparent molecular weights and high percentages of random coil conformations, as measured by size exclusion chromatography and CD, respectively (Sponga et al., 2021). They additionally obtained a diverse ensemble of atomistic-resolution structures by fitting a pool of randomly generated models to SAXS data (Sponga et al., 2021). Lastly, using binding assays, NMR, X-ray crystallography, and SAXS, the authors revealed that the intrinsically disordered MYOZ1 forms tight, fuzzy interactions with α-actinin (Sponga et al., 2021). This raises the possibility that myozenin 2 may exhibit similar interactions with cardiac α-actinin that are important to its function. Our PONDR scores for MYOZ2 indicate that the N-terminal fragment and C-terminus of the protein are IDRs (Fig. 3). Although no pathogenic or likely pathogenic variants were identified in the predicted IDRs of MYOZ2 from the ClinVar database, two HCM mutations, S48P and I246M, have been identified in the N- and C-termini, respectively (Ruggiero et al., 2012). The PhosphoSitePlus database reports five PTMs in the predicted IDRs (Fig. 3).

*Spectrin beta, erythrocytic (SPTB)* is a 247 kD protein that binds to the actin filament (An et al., 2005) and is a major structural component of the cytoskeleton (Winkelmann et al., 1990). SPTB binds to α-spectrin to form hetero-tetramers (Long et al., 2007). Although both α and β spectrins are largely characterized as coiled-coil structures (Park et al., 2003), at least the N-terminus of α-spectrin and residues Q1898–E2083 of β-spectrin were determined to contain IDRs, as determined by CD and NMR studies (Park et al., 2003; Long et al., 2007). Spectrin isoforms bind at these IDRs and gain helical character after binding (Long et al., 2007). Our PONDR scores suggest that many IDRs span the SPTB sequence, including those already confirmed to be intrinsically disordered (Long et al., 2007). Within these predicted IDRs, PhosphoSitePlus and ClinVar report 13 PTMs and 18 pathogenic or likely pathogenic variants, respectively.

### Properties of MAPIDs and their characterization

#### Advantages and vulnerabilities of IDRs in MAPIDs

Nearly all the proteins we describe in section ‘Myofilament-associated protein with intrinsic disorder (MAPID)s’ have regions that were either determined by experiment to be IDRs or were suggested to be disordered by PONDR. The abundance of these regions suggests that they perform distinct functional roles relative to their folded counterparts. We speculate there are several advantages to these IDRs in myofilament proteins.

##### Native structure.

IDRs’ roles in cellular signaling and transcription are well-known (Gibbs and Showalter, 2015; Clarke and Pappu, 2017). This involvement is likely due to their transient structures that can afford functional advantages over folded domains. For one, IDRs permit more rapid and efficient regulation compared to their folded counterparts (Uversky et al., 2005; Sugase et al., 2007). They can bind partners with broad selectivity and significant avidity; avidity refers to enhancing binding through many low-affinity poses in equilibrium (Wright and Dyson, 2015; Erlendsson and Teilum, 2021). These low affinity interactions are frequently characterized by fast association and dissociation kinetics that enable switch-like changes in function (Wright and Dyson, 2015). For IDR-mediated PPIs, there is likely also a desolvation energetic advantage, as many IDPs have hydrophobic binding interfaces and undergo apolar desolvation upon binding to targets (Gibbs and Showalter, 2015).

IDRs are ubiquitous in myofilament proteins, which suggests their importance in muscle contraction and regulation (Best, 2017; Clarke and Pappu, 2017). To quantify this propensity, we used PONDR (Obradovic et al., 2003) to predict disordered regions within the genes listed in section ‘Myofilament-associated protein with intrinsic disorder (MAPID)s’. These results are summarized in Fig. 3. We provide pie charts for each of the MAPIDs, categorized into thin/thick filaments(s), Z-disk, and miscellaneous, for which gray denotes folded regions versus red for IDRs. The MAPIDs we consider in this review are predicted to contain IDRs, though we recognize that some of these proteins are known to be folded as isolated (TNNC1) or co-assembled (ACTC1) proteins. These discrepant examples may just be due to inaccuracies of the PONDR algorithm. Alternatively, they may support the idea that all proteins are composed of both folded and disordered regions, but to varying degrees (Sormanni et al., 2017). For instance, the predicted IDRs in TNNC1 all contain loops that resemble IDRs in the absence of Ca^2+^ (Fig. S1).

We next show how the IDR primary sequence affects the ensemble topology using an IDP state diagram developed by Pappu and coworkers (Das and Pappu, 2013; Das et al., 2015; Holehouse et al., 2017) (Fig. 3a). This diagram relies on two inputs, $f_{+}$ and $f_{-}$, that reflect the fractions of positively and negatively charged residues, respectively. These inputs classify IDRs into five ensemble states that differ in compactness (see the legend of Fig. 3 for detailed descriptions of these states). These state diagram coordinates allow us to estimate the approximate topology of the predicted IDRs (Figs 3b–3e). For instance, for MYL7 we see that most of its IDRs fall within the R1 and R2 regions that are characterized as ‘premolten globules’ and the coexistence of globules and coils, respectively. However, one IDR is described as a swollen coil topology. Other MAPID examples feature IDRs that span diverse topologies. It is not yet understood if the distributions of topologies are by chance or are shaped by their respective roles in regulating myofilament function.

##### Post-translational modifications.

PTMs provide a mechanism to rapidly, and often reversibly (Jideama et al., 2006), perturb the ensemble from its native (unmodified) configuration. Just as the state diagrams show how an IDP’s charge distributions determine its conformation ensemble topologies, they can be used to predict how PTMs may alter the native ensemble. Phosphorylation is a well-studied PTM from the broad variety of modifications found among MAPIDs. Phosphorylation is a form of chemical modification of tyrosines, serines and threonines that alters a given IDR’s charge distributions, changes local/non-local electrostatic interactions (inter- and intrachain), and impacts solvation. Moreover, IDRs usually have multiple phosphorylation sites (Martin et al., 2016) that can amplify those changes. Human cardiac TnI, for instance, has 14 PTMs that have so far been characterized, of which the majority are phosphorylations (Biesiadecki and Westfall, 2019). These PTMs are believed to be the cornerstones of TnI’s regulatory role on myofilament function in response to kinase activities (Biesiadecki and Westfall, 2019). Of these, a host of phosphorylation sites on the disordered N-terminus of TnI influence its conformation and are implicated in weakening the Ca^2+^ sensitivity of TnC (Cheng et al., 2014; Lindert et al., 2015; Zamora et al., 2016).

To illustrate this potential across the broad MAPID family, we collected phosphorylation sites within predicted IDRs from the PhosphoSitePlus database (Hornbeck et al., 2015) (see section S1 for methodology). The statistics reported in Figs 3(b)–3(e) demonstrate that MAPID IDRs are rife with PTMs, for which only 5 out the 31 proteins (MYL3, MYL7, TNCC1, CRIP3, FHL2) do not yet report PTM sites in the predicted IDR regions. Genes with the most abundant PTMs include ANK2 and TTN with 119 and 338, respectively. We next show how these phosphorylation sites may affect the IDRs’ ensemble properties using the IDR state diagrams (Figs 3b–3e). To mimic the negative charge brought by phosphorylation, we mutated the residues identified as PTM sites to glutamic acids, which altered the IDR charge distributions. The pink dots in Figs 3b–3e highlight the corresponding changes in ensemble topology upon phosphorylation. Since the phosphate groups modulate the intrinsic charge and charge density of the IDRs, the ensemble properties for the modified proteins are shifted relative to the unmodified states. For many examples, the modified *versus* unmodified proteins’ distributions fall in different phases of the diagrams. As an example, the unmodified myosin heavy chains (MYH6 and MYH7) are distributed along the R2/R3 borders, which represent the coexistence of random coils and globules. The phosphorylation-modified states, however, are redistributed toward the R3/R4 border, suggesting an ensemble change to relatively extended swollen coils. Although it remains unclear which conformational ensembles are important for myosin heavy chain function, we speculate that topological changes may enable IDRs’ influence on myofilament function to be tuned, such as to increase the likelihood of a PPI or to enhance its avidity.

##### Single nucleotide polymorphisms.

The apparent importance of IDRs in myofilament function and PTM suggests that missense mutations in these regions similarly perturb myofilament function. To assess this potential, we retrieved pathogenic and likely pathogenic variants for each protein from the ClinVar database. We focused on the protein-changing variants, e.g. those resulting in missense mutations or deletions. Of the 36 proteins considered for this study, only 8 proteins did not have reported protein-changing variants. These include ABLIM1, CRIP3, FHL2, SYNPO2, TGM2, TMOD1, MYL7, and ENH2. We report in Fig. 3 disease-related mutations that reside in predicted IDRs. Similar to phosphorylation, missense mutations that alter an IDR’s intrinsic charge have the potential to alter the IDR topology. Those that conserve charge could still impact native structure, depending on the side chain properties or backbone torsion angles (Jumper et al., 2018). Here we find that potentially pathological missense mutations are found in most of the MAPID IDRs, which is consistent with observations made for proteins in general (Vacic and Iakoucheva, 2012). In contrast to the seemingly uniform PTM abundance across the core and associated protein sets, the disease-related mutations are more prevalent in the core proteins. This may imply that either disturbance of core protein functions presents a more obvious phenotype than the associated proteins, or simply that the core proteins have been genotyped to a greater degree.

#### General challenges in characterizing native IDR structures

**Fig. 5.**
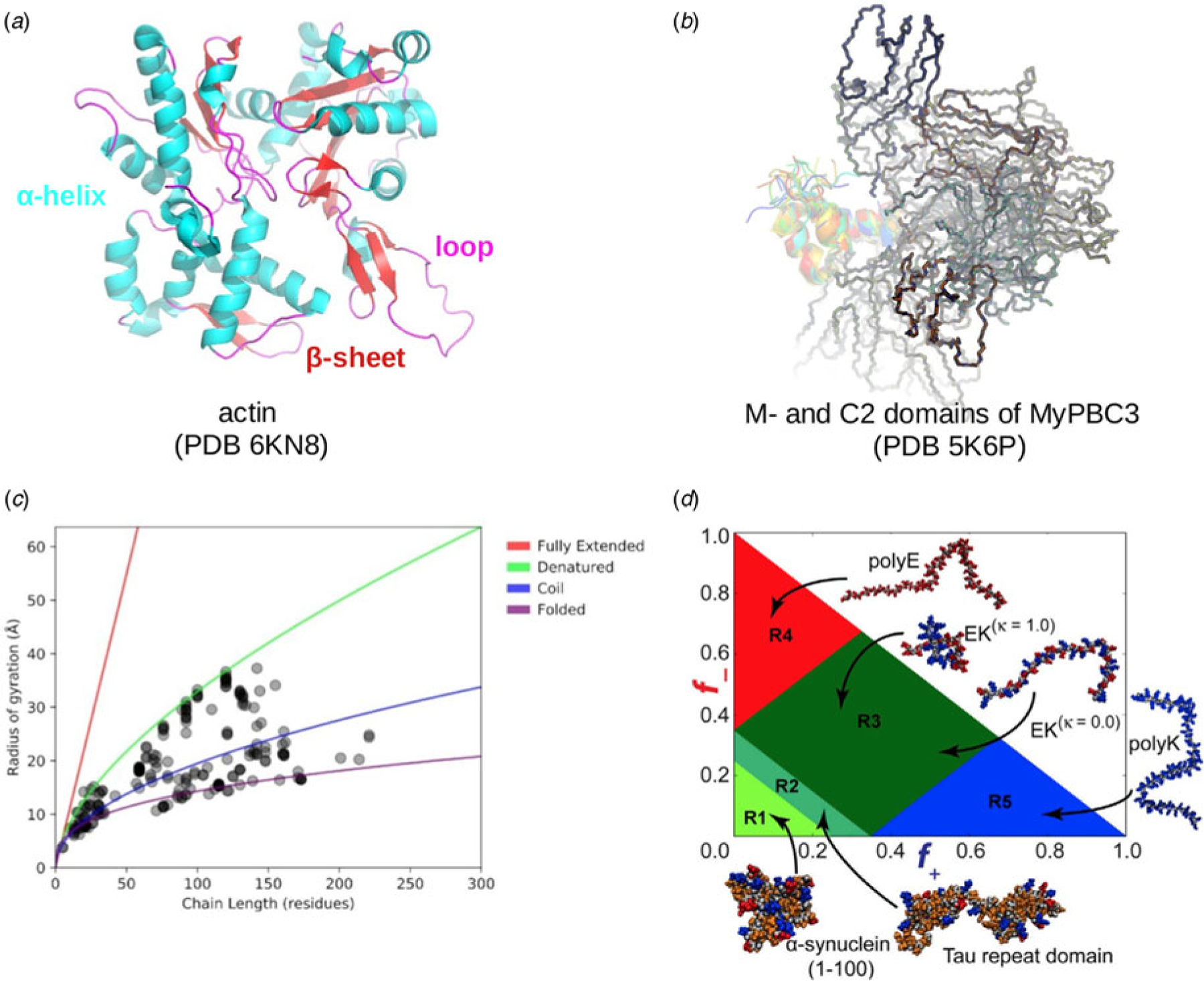
(*a*) Major secondary structural elements present in folded proteins like actin (PDB 6KN8 (Yamada *et al*., 2020)). (*b*) NMR structures of a MAPID, the MyBPC3 construct consisting of the M- and C2 domains (Michie *et al*., 2016), are shown as an example to illustrate the IDR ensemble. (*c*) Scaling of radius of gyration $\left(R_{g}\right)$ versus chain length for proteins at different states (Lazar *et al*., 2021). (*d*) The IDP phase-diagram for classifying IDPs into five structural states (R1–R5) based on the charge patterns (Holehouse *et al*., 2017).

Bioinformatic approaches generally determine *whether* an amino acid sequence would give rise to a disordered polypeptide, but do not in general predict their physicochemical properties. Many of these approaches rely on the observation that IDPs lack the hydrophobic core typical of globular proteins, and are instead enriched with charged and polar residues (Clarke and Pappu, 2017). These residues in turn determine the local/non-local intrachain interactions, solvent/protein interactions, and ultimately IDP ensemble properties (Fig. 5). A key challenge for understanding these interactions and properties in IDRs is that their complete conformation ensembles must be resolved instead of a single conformational state. This is because IDRs have rugged potential energy surfaces that present numerous conformations in equilibrium that rapidly interchange. Relatedly, unlike globular proteins whose folding landscape is usually single-funneled, IDPs’ energy landscapes are multi-funneled, which significantly challenges structure prediction tools such as AlphaFold (Strodel, 2021). Altogether, the large number of poorly resolved, functional conformations, and their equally important transition kinetics, pose fundamental challenges for the understanding of structure and function relationships among MAPIDs.

##### The folded and disordered continuum.

Dividing proteins into ordered and disordered states is an oversimplification. Recent experiments, especially solution NMR techniques that can capture both conformations and their dynamics, suggest that protein ensembles are heterogeneous and contain both ordered and disordered components (Sormanni et al., 2017). Similarly, IDRs can contain fragments with folded character. These are called residual structures and are of great importance to protein functions, including binding to protein partners (Wicky et al., 2017). It is increasingly recognized that proteins function as ensembles and even the so-called native states of folded proteins consist of multiple conformations (Gibbs and Showalter, 2015). Therefore, an IDR represents an extreme case of this paradigm (Gibbs and Showalter, 2015), which we illustrate with two examples. As one example, the switch peptide region of TnI (residues R148–K164) is disordered in the absence of a target, but adopts an alpha-helix upon binding Ca^2+^-activated TnC (Lindert et al., 2015; Cool and Lindert, 2021). Another example is the M domain of MyBPC3 (residues T255–R357), which retains a small tri-helix bundle that is believed to bind myosin (Howarth et al., 2012; Michie et al., 2016; Singh et al., 2021). Along these lines, loops or linkers between folded elements like helices (such as TnC’s loops), and N/C termini (such as TnI) are frequently disordered (as has been predicted by PONDR in Fig. 3). The disorder inherent in these linkers can influence the function of folded domains (Sun and Kekenes-Huskey, 2022), as opposed to just serving a passive role in linking the folded domains.

##### Modifiers of IDR ensembles.

Physicochemical properties of IDRs are encoded both by the number of covalently bonded amino acids and non-covalent properties bestowed by their side chains. Non-covalent interactions of an IDR with its environment help determine its properties. We discuss these factors in detail below.

*Covalent interactions* The ‘size’ of an IDR is often characterized by its radius of gyration $\left(R_{\text{g}}\right)$. Intuitively and generally, the longer the IDR’s sequence, i.e. the number of covalently linked amino acids, the larger its $R_{\text{g}}$. Analytic approximations for $R_{\text{g}}$ as a function of the numbers of amino acids are explained in section ‘Implicit/semi-analytic representations’. Independent of the non-covalent effects discussed below, covalent character can influence IDR properties in two ways. (1) Crosslinking of an IDR through disulfide bonds will reduce an ensemble’s $R_{\text{g}}$. (2) Differences in amino acid backbone distributions, often characterized by their ϕ/ψ (Ramachandran) angles, can shape the IDR conformation ensemble. Prolines and glycines, as an example, constrain and relieve constraints respectively (Huang and Nau, 2003), on peptide backbone conformations. More generally, the ϕ and ψ dihedral angles assumed by contiguous amino acids support secondary structure inclusive of beta sheets, helices (α-helix/π-helix/3–10 helix), and turns, though non-covalent interactions arising from hydrogen bonding ultimately stabilize these structures.

*Non-covalent interactions* The abundance of polar amino acids that favorably interact with a polar solvent, water, relative to hydrophobic residues is a strong determinant of IDR character. These properties constitute non-covalent effects that simply dictate whether an amino acid prefers (thermodynamically speaking) to interact with the solvent or with the solute protein.

*Hydrophobicity and hydrophilicity* Well-folded proteins comprise hydrophobic residues that are thermodynamically disfavored to interact with polar solvents. For this reason, there is a thermodynamic driving force for hydrophobic residues to coalesce into compact domains that minimize polar solvent interactions. This occurs despite the loss of entropy that occurs during protein folding (Cheung et al., 2002). Conversely, polar residues favor interactions with both other polar residues and solvent. In many cases, since the enthalpy of a polar amino acid’s interaction with water or other polar residues are similar, maximizing entropy favors unfolded states of the protein (Toal et al., 2014). For this reason, IDRs tend to have an abundance of polar and charged amino acids (Lieutaud et al., 2016).

**Fig. 6.**
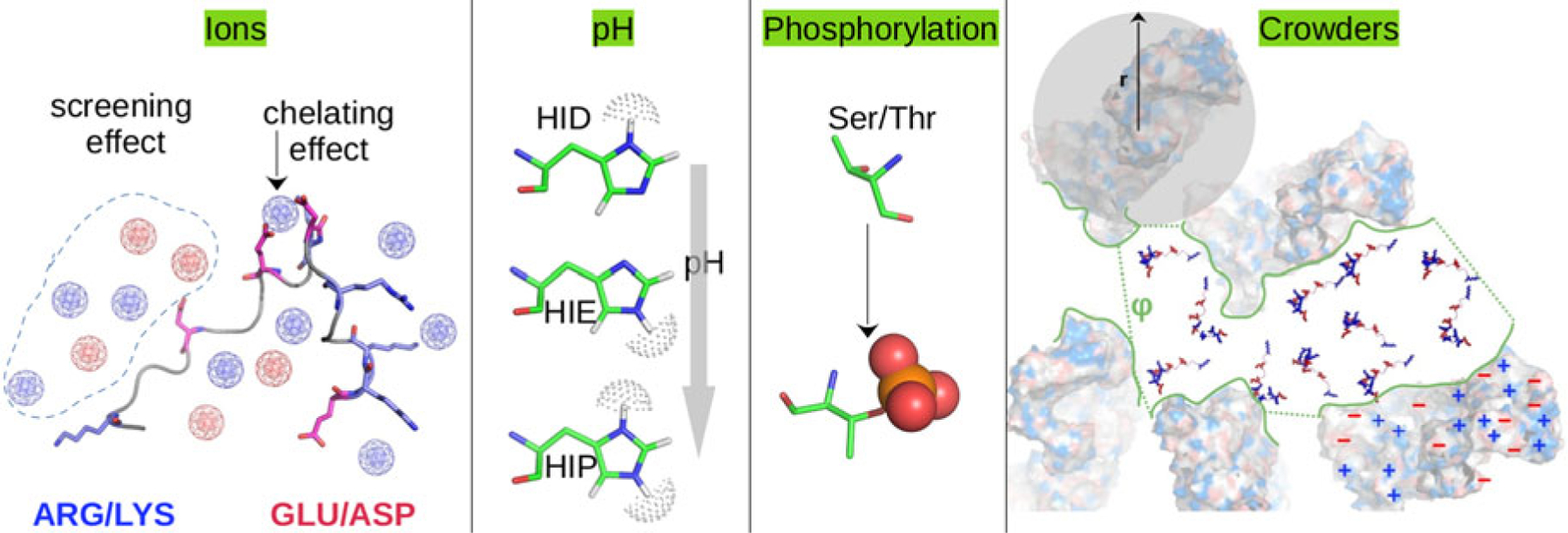
Schematic of transient and covalent influences on IDR structure. Ions can screen intramolecular electrostatics or directly coordinate with charged residues. pH affects the protonation state of ionizable residues (e.g. histidine). Phosphorylation introduces negative charges into the sequence. For crowding, many factors such as size and surface charges of crowders, and volume fraction affect IDR structure (Eq. (7)).

*Electrostatic effects* A solute’s free energy of solvation depends on its charge, the solvent dielectric, and the presence of other charged solutes (Eq. (9)). Hence, the driving force for folding *versus* maintaining an intrinsically disordered, unfolded configuration will depend on the solute’s environment and its physicochemical properties (Fig. 6). Factors of the environment include changes in ionic strength and temperature (see κ and T in Eq. (9)), as well as the presence of crowders or organelles that impact charges $\left(q_{\text{i}}\right)$ and dielectric constants $\left(\epsilon_{\text{r}}\right)$. Physicochemical properties can include reversible (protonation, phosphorylation, and Nϵ-acetylation) and frequently irreversible (oxidation, glycation, and Nα-acetylation) chemical changes to a protein’s structure (Vu et al., 2018; Narita et al., 2019).

*Ions* Ionic strength changes follow the addition or removal of ionic species (monatomic species such as K^+^ and Cl^−^, as well as molecules like ATP^4−^), denaturants like guanidinium chloride, or species that impact pH (discussed below). Ions primarily modulate IDR structural properties in two ways: they directly interact with IDR residues to modify net charges or screen intrachain electrostatic interactions (Uversky, 2009). In the former case, binding ions such as Zn^2+^ change the net charge of a residue or even ionically link residues to exert a direct impact on an IDR’s secondary structure (Uversky, 2009). The dependence of these factors on ion types has been reported in Wicky et al. (2017). Namely, the authors found that the residual structures of an IDR in its isolated state and the transition state for binding are sensitive to different types of ions, resulting in altered association and dissociation kinetics. Similarly, ions can bind to and interfere with the dynamics of mobile loops in proteins, as was shown for the binding of Ca^2+^ to titin’s Ig domains (Kelly et al., 2021). In addition, ions can screen electrostatic interactions, which is common in physiological conditions. The extent of this effect as a function of ionic strength can be estimated via the Debye–Huckel model (Eq. (9)). At high ionic strength, ions can have a ‘salting-out’ effect that reduces amino acid solubility. This effect is dependent on the type of ion (Hofmeister series) (Wohl et al., 2021).

*pH* The pH quantifies the balance of H^+^ and OH^−^ ions in solution. IDR ensemble properties can be altered by pH through changing the protonation of ionizable amino acids or ionic strength (Sun et al., 2020). Protonation changes in turn alter an IDR’s charge distribution and salt-bridges. Ionization state changes are most apparent in very acidic or basic conditions, where dramatic structural changes may be evident (Uversky, 2009). The PEVK repeats of titin exemplify this effect, as it shows a pH-dependent compaction of its structure (Manukian et al., 2022). An IDR prediction tool named DispHred has been developed to characterize disorder propensity as a function of pH (Santos et al., 2020).

*Crowding* Crowders refer to molecular and subcellular material that vary in terms of charge density, volume, capacitance (membranes), and internal dielectric constants (Fig. 6). Crowders can modify an IDR ensemble (Banks et al., 2018), by altering the free energies of the unfolded, solvated states. In a dilute solution, configurational entropy is maximized (reducing the free energy) as a polymer disperses into the medium. In the presence of crowders, the space within which the polymer can disperse is reduced, which in turn reduces the entropy gain. Hence, crowding can shift the balance between folded and unfolded states, simply by reducing the free volume that a polymer can occupy. Along these lines, it is intriguing that the spacing between myofilaments changes occurs during contraction, which in principle could modulate the free volume available to MAPIDs (Irving et al., 2000). An excellent review on the topic of how crowders influence protein thermodynamics was published by Zhou (2008).

*Post-translational modification (PTM)* Post-translational modifications represent chemical (covalent) changes to protein structure that typically alter the non-covalent properties of an IDR. These can include increasing the size of an amino acid, its charge, and even modify inter-/intraprotein contacts. PTMs provide a reversible mechanism for rapid modulation of a protein’s structure, protection against reactive species, and tagging for degradation or subcellular transport (Vu et al., 2018). IDRs usually have multiple phosphorylation sites (Martin et al., 2016), which provides a reversible means to alter a protein’s charge distribution, local and non-local electrostatic interactions, and solvation thermodynamics (Martin et al., 2016). However, at least in some cases, global properties of IDRs such as the radius of gyration are insensitive to phosphorylation owing to compensatory changes in intrachain interactions (Martin et al., 2016). This suggests cell signaling may recognize local changes in sequence and charge, rather than changes in the global structure of the conformational ensemble. For example, many IDRs have SLIM regions that are used for binding protein targets. Since PTMs are abundant among IDRs and approximately 2000 SLIMs have already been identified (Lindorff-Larsen and Kragelund, 2021), we speculate that PTMs could be an important mechanism for toggling the availability of SLIMs for target binding.

A well-studied example of PTMs among MAPIDs include phosphorylation of TnI at Ser22/23 in its N-terminal IDR, which alters TnI binding to TnC (Hwang et al., 2014). Another includes MyBPC3, for which phosphorylation impacts myofilament contraction by altering its interactions with actin (Previs et al., 2016). In recent years, broad data sets of PTMs in MAPIDs have been identified through bottom-up (proteolytic digestion before mass spectrometry) (Kooij et al., 2014) and top–down (digestion-free) (Zabrouskov et al., 2008) mass spectrometry approaches. Extensive reviews of PTM types are in the literature (Deribe et al., 2010; Prabakaran et al., 2012), but their impacts beyond phosphorylation and oxidation have not been extensively investigated in myofilament proteins, much less MAPIDs. This knowledge is critical for understanding both regulatory and dysregulated functions of proteins with IDRs (Uversky, 2014), including MAPIDs.

#### Challenges specific to MAPIDs

Many challenges have stymied the characterization of IDRs in MAPIDs. The staggering size of some myofilament proteins is one such challenge. Titin, as an example, consists of approximately 34 000 residues, of which nearly 35% of its primary sequence is predicted to be intrinsically disordered by PONDR (Obradovic et al., 2003) (Fig. 3). However, extensive simulations and experimental techniques that are commonly used to probe IDRs (Robustelli et al., 2020; Chang et al., 2021; Ding et al., 2021) are best suited for much smaller proteins. Therefore, many studies of intact MAPIDs are limited by the large number of disordered residues, as well as the likely presence of multiple disordered regions within a single protein.

It is also recognized that the *in vitro* environment complicates the identification of IDRs. The myofilament is a complex structure consisting of dozens of proteins that function interdependently. As an example, actin has a well-folded structure in complex with troponin, and tropomyosin as shown in the crystal structure resolved by Yamada *et al*. (PDB 6KN8 (Yamada et al., 2020)). At the same time, actin exhibits promiscuous target binding that is a signature of IDPs (Povarova et al., 2014) and is also predicted to contain several IDRs (Turoverov et al., 2010; Povarova et al., 2014). In addition, actin uses chaperones to fold onto the thin filament (Grantham, 2020). These observations suggest that the presence of other accessory proteins in an MAPID’s environment can dictate the extent of its folding.

IDRs also often undergo disordered-to-ordered transitions, such as during a coupled binding and folding process (Sugase et al., 2007). The C-terminal region of TnI provides an excellent example of this phenomenon. When the N-terminal domain of TnC is free of Ca^2+^ during diastole, its hydrophobic domain is closed and thus unavailable to bind the TnI C-terminal domain. This TnI domain therefore remains unbound and assumes an intrinsically disordered ensemble as determined via NMR (Julien et al., 2011). At saturating calcium during systole, TnC’s hydrophobic domain is unveiled and supports binding of the TnI ‘switch’ peptide, during which the region folds into an alpha-helix (see Fig. 5a, e.g.). Modeling these processes is non-trivial and often suffers from force field inaccuracies and sampling insufficiency (Pietrek et al., 2020).

Lastly, there remains a challenge of relating computation-predicted IDR ensemble properties to experimental probes of the myofilament. A main reason for this is that there are spatial and temporal gaps in the resolution of experimental *versus* computational data sets. We refer to our experience with TnI binding to TnC, where ultimately the properties of TnI’s conformational ensemble determine its experimentally observed affinity to TnC (Siddiqui et al., 2016). Although multi-state models that take molecular-level IDR descriptions as inputs are helpful in predicting myofilament observables like binding affinity (Siddiqui et al., 2016; Sun and Kekenes-Huskey, 2020), these models are often system-specific and difficult to generalize to other IDR-driven processes.

## Part 2: computational modeling and characterization of MAPIDs

In this part, we overview the state-of-the-art computational methods used to characterize IDRs and IDPs. We organized this section as a potential workflow for an investigator to characterize a newly identified IDR. Hence, we divide these approaches into those targeting ensembles of IDRs in steady state (section ‘Computational methods for predicting conformation ensembles of isolated MAPIDs’), the kinetics of intramolecular dynamics (section ‘Computational methods for predicting intramolecular dynamics of MAPIDs’), and complex assembly (section ‘Computational methods for predicting the MAPID co-assembly’). In each section, we motivate the specific challenge for each type of approach with a brief synopsis of relevant experimental techniques, since excellent reviews of experimental methods for IDRs are readily available (see Gibbs and Showalter, 2015; Schramm et al., 2019). These synopses are followed by detailed summaries of commonly used computational approaches and their applications to MAPIDs, when available.

### Computational methods for predicting conformation ensembles of isolated MAPIDs

#### Problem and application

A primary goal in the characterization of proteins with IDRs is to describe their structure and physicochemical properties under equilibrium and steady-state conditions. We distinguish this challenge from that of assessing intraprotein kinetics and intermolecular assembly that we discuss in subsequent sections. Characterizing the steady-state properties of isolated IDRs typically entails describing the spatial extent of the conformation ensemble, the predominant conformations forming the ensemble, as well as the secondary, tertiary, and quaternary structure of the conformations. Also of interest is how the molecular and environmental factors summarized in section ‘Modifiers of IDR ensembles’ alter the ensembles’ properties. These insights provide an initial foundation for understanding the biological role of MAPIDs. Such is the case for studies seeking to characterize the disordered switch region in TnI that primes its interaction with TnC (Lindert et al., 2015; Siddiqui et al., 2016; Cool and Lindert, 2021). Equilibrium properties like the shape of an IDR’s ensemble can impact physiologically important parameters such as myofilament length (nebulin (Wu et al., 2016)) and passive tension (titin PEVK motifs (Yamasaki et al., 2001)). The ensemble’s shape also impacts the ability to assemble macromolecular structures or bind targets (discussed further in section ‘Computational methods for predicting the MAPID co-assembly’) to engage in PPIs, such as TnC binding to TnI (Metskas and Rhoades, 2015; Siddiqui et al., 2016; Cool and Lindert, 2021).

#### Experimental techniques

The state-of-the-art for the experimental characterization of IDPs has been robustly reviewed (see, e.g. Gibbs and Showalter, 2015; Schramm et al., 2019), therefore here we briefly introduce methods that are commonly used in tandem with computational approaches. Among these include SAXS, NMR, CD, hydrogen/deuterium exchange mass spectrometry (HDXMS), and FRET. Generally, SAXS provides information on gross protein shape and compactness, CD assesses the relative abundance of secondary structure content, NMR and HDXMS can monitor conformational dynamics, and FRET can probe quaternary structure or oligomerization (Schramm et al., 2019; Metskas and Rhoades, 2020). In addition, cryo-EM and atomic force techniques have also been used to study IDP oligomerization and mechanical parameters (Karsai et al., 2011; Mostofian et al., 2022). These methods can also be used in combination. Because the aforementioned methods are priorly used for probing equilibrium properties of IDRs, we extensively discuss the fundamentals of each technique in this section. Specific applications of these methods to the kinetics of IDR ensembles and kinetics are discussed briefly in subsequent sections of the review.

##### Size-exclusion chromatography.

Size-exclusion chromatography is used to separate proteins by their sizes according to their elution time in a porous column. The elution time of a protein is related to its Stokes radius:

(1) $$R_{\text{s}}=\frac{k_{\text{B}}T}{6\pi \eta D}$$

where η is the solvent’s viscosity, D is the diffusion coefficient, $k_{\text{B}}$ is Boltzmann constant and T is temperature. Proteins with the same mass but larger Stoke radius will diffuse through the column instead of being captured by its porous interior and thus elute earlier than would be predicted from the protein mass alone (Schramm et al., 2019). A larger Stokes radius is indicative of an unfolded protein relative to a folded protein of a similar size. This method has similar advantages and disadvantages relative to electrophoretic probes of mobility discribed next.

$R_{\text{g}}$ is a commonly used metric to assess the size of an IDP. Although the $R_{\text{g}}$ and the Stokes radius $\left(R_{\text{s}}\right)$ are typically measured by different methods (static scattering measurements for $R_{\text{g}}$ and dynamic light scattering for Stoke radius, respectively), they are intrinsically related (Tande et al., 2001). For a hard sphere, $R_{\text{g}}\text{/}R_{\text{s}}\approx 0.77$, and this ratio changes with the aspect ratio of the polymer. A rod-like polymer can have a ratio of up to 1.27 (Tande et al., 2001). Size exclusion chromatography has been used quite extensively for MAPIDs. These applications include probes of titin’s PEVK motifs (Duan et al., 2006) and the disordered C-terminus of ankyrin B (Abdi et al., 2006), as well as assays using fesselin, a homologue of synaptopodin 2 (Khaymina et al., 2007), and FATZ-1 (Sponga et al., 2021).

##### Gel electrophoresis.

Gel electrophoresis leverages the principle that proteins with different molecular weights and net charges have different mobilities in an applied electric field. There are two kinds of gel electrophoresis: (1) native gel electrophoresis, and (2) SDS-PAGE (sodium dodecyl sulfate–polyacrylamide gel electrophoresis). For SDS-PAGE, the protein’s intrinsic charges are masked by the sodium dodecyl sulfate. This masking enables all protein components to adopt similar charge-to-mass ratios, thus proteins are separated solely based on their molecular masses. In the native gel, proteins migrate in their native states; therefore, the migration is affected by the protein’s intrinsic charge, size, and folding state (Arndt et al., 2012).

Both strategies are useful tools for IDP studies. IDPs usually have larger apparent molecular masses than would be expected for a folded protein due to (1) their unique residue compositions, e.g. high percentages of charged residues and less hydrophobic residues, which make them bind less strongly to SDS, thus they migrate slower, (2) their unfolded states yield large Stokes radii, and (3) high proline content that leads to stiff conformations (Schramm et al., 2019). Therefore, SDS-PAGE measurements of mobility are commonly used to probe whether a given protein has intrinsically disordered content.

The native gel is useful to infer the binding and folding of IDRs. As an example, Neirynck *et al*. probed how the CCT chaperone folds actin using native gel electrophoresis (Neirynck et al., 2006). In that experiment, an alanine scan was performed on actin, whereby amino acids speculated to bind the chaperonin were mutated to alanine to assess if the binding interactions were compromised. The mutagenesis of amino acids that were directly involved in chaperone binding was identified by their impact on the electrophoresis of the variant actin relative to wild-type (Neirynck et al., 2006). A primary advantage of gel electrophoresis for assessing IDR characteristics is that it is a straightforward and inexpensive technique to perform. The main disadvantages are that these analyses do not directly provide structural insights and the migration-based assessments of IDRs are qualitative. There are other factors such as detergent binding that can also affect migration (Rath et al., 2009).

##### Circular dichroism (CD).

CD is a technique that relies on the polarization of incident light through a sample. In the context of protein structure determination, secondary structures such as α-helices and β-sheets (see Fig. 5) rotate light at different angles. Hence, the secondary structure content can be monitored in IDPs to determine unfolded to folded transitions or partially folded domains in an IDR. The primary advantage is that CD can work with small concentrations of proteins with wide-ranging sizes. A disadvantage is that CD spectra of β-sheet structures are quite broad and overlap with α-helices (Micsonai et al., 2015), thus limiting the analysis to qualitative assignment of secondary structures. CD has been used for probes of many MAPIDs, including titin’s PEVK motifs (Ma and Wang, 2003; Duan et al., 2006), ankyrin B’s C-terminus (Abdi et al., 2006), tropomodulin’s intrinsically disordered N-terminus (Greenfield et al., 2005), and for FATZ-1 (Sponga et al., 2021). Other examples are listed in Table 1.

##### Small-angle X-ray scattering (SAXS).

SAXS is an approach that measures the scattering of X-rays upon colliding with a solute in solution. In most applications, SAXS data are reported in Guinier plots that summarize the intensity of scattered X-rays *versus* the scattering vector. The radius of gyration $\left(R_{\text{g}}\right)$ is readily obtained from this analysis, which lends itself to the assessment of IDR sizes (Zheng and Best, 2018):

(2) $$\ln\frac{I(q)}{I(0)}=\left(-R_{\text{g}}^{2}\text{/}3\right)q^{2}$$

where q is the scattering vector, I(q) is the SAXS intensity at the scattering vector, q. I(0) is the intensity when q=0. The I(q) profile from the Guinier plot also exhibits profiles that can indicate a protein ensemble’s shapes, such as spherical *versus* oblong (Grupi and Haas, 2011). A popular SAXS-based IDR ensemble generation strategy, called the ‘ensemble optimization method (EOM)’ (Bernadó et al., 2007), has been developed for which conformations are picked from a pool of conformations randomly generated by simulation to match SAXS data. Advantages of this method are that it is a label-free technique that can yield structural information for arbitrarily large proteins and macromolecular complexes. A chief limitation of the method is that access to a synchrotron is needed for an X-ray source. Examples of MAPIDs characterized by SAXS include FATZ-1 (Sponga et al., 2021) and the M-domain of MyBPC3 (Michie et al., 2016).

##### Fluorescence spectroscopy.

The intrinsic fluorescence of aromatic residues, namely tryptophan and to a far lesser extent tyrosine and phenylalanine, can be measured using fluorescence spectroscopy. Changes in fluorescence indicate differences in the amino acids’ environment (Ghisaidoobe and Chung, 2014), such as due to folding or the binding of targets. For instance, tryptophans buried inside the hydrophobic core of folded proteins are brighter and blue-shifted relative to those exposed to aqueous solvent. Upon solvent exposure of tryptophans due to unfolding, the emission spectrum red-shifts and dims (Khaymina et al., 2007; Yang et al., 2015). The advantage of this technique is that there is no need to introduce extrinsic fluorescent probes and generally fluorimeters are inexpensive. Disadvantages include the low extinction coefficient that results in dim signals and ambiguity when more than one fluorescent species is present. Intrinsic fluorescence has been applied to synaptopodin 2’s homolog fesselin to show that the fluorescent signals of the tryptophan residues have maxima around 334 nm, suggesting that those residues are solvent-exposed and therefore the protein’s native state is unfolded (Khaymina et al., 2007).

##### Förster resonance energy transfer (FRET).

FRET is another fluorescence-based technique frequently used in protein structure characterization. The method relies on stimulating a donor fluorophore at its absorption wavelength, after which it emits at longer wavelengths. An acceptor fluorophore can absorb the energy and emit the energy at an even longer wavelength. The strength of this energy transfer decays as $I(r)=I(0)\text{/}r^{6}$, where r is the distance between donor and acceptor fluorophores. Given this rapid spatial decay of the intensity, FRET is commonly used to measure 1–10 nm distances between probes. An additional advantage is that the technique can use more than one pair of probes (Lee et al., 2015). A disadvantage of the method is that a chemical or protein construct, such as green fluorescence protein, is typically introduced to the protein of interest, which can alter its structure. A study of IDRs in troponin I made extensive use of FRET to resolve the positioning of their conformations relative to folded troponin C structures (Metskas and Rhoades, 2015).

##### Nuclear magnetic resonance spectroscopy (NMR).

NMR-based techniques have been extensively used to determine the structures of modestly sized (commonly <30 kD (Xu et al., 2006)) isolated proteins and protein/protein complexes at Angstrom-level resolutions that can rival X-ray crystallography. The list of NMR-based techniques amenable to probing IDPs is ever-growing (reviewed in Schneider et al., 2019; Dyson and Wright, 2021). In short, NMR leverages the interaction between an applied magnetic field and the nuclear spins of atoms. The applied magnetic field splits into spectral lines, or energy levels, of different energies (Zeeman effect). Those energies may shift depending on their local environment (chemical shift). These properties enable one to assign peaks in NMR spectra to specific nuclei types in functional groups (typically hydrogen, carbon, and nitrogen), as well as determine their proximity to other functional groups such as aromatic rings. 2D heteronuclear NMR broadens this approach by exciting different nuclei and accounts for the relaxation times for coupled spins, which improves the ability to resolve and assign peaks.

Excited nuclear spins can be transferred to nearby, coupled nuclear spins (nuclear overhauser effect, NOE), which enables the determination of distances between coupled nuclei. This approach is generally more readily applied to folded proteins. In complement, residual dipolar couplings (RDC)s, which measure the angles between partially ordered magnetic nuclei and the magnetic field (Prestegard et al., 2004), are also increasingly used in IDP experiments (Fisher and Stultz, 2011). In addition, order parameters that report on the mobility of bond vectors, such as N-H, can also be measured from spin relaxation rates. Overall, NMR spectroscopy offers a wide-array of techniques that can be used to probe both steady-state and dynamic properties of protein conformation ensembles in IDPs (Schneider et al., 2019; Dyson and Wright, 2021). Disadvantages of these approaches include the need for large amounts of protein, sample preparation such as labeling that can vary considerably in cost, and expensive instrumentation.

NMR techniques for IDPs and proteins with IDRs have been applied to several MAPIDs. Ma *et al*. used 2D H-H NMR (TOCSY and ROESY) to probe the residual structure of PEVK motifs in titin and how the disordered content changes with temperature and ionic strength (Ma and Wang, 2003). Other applications used ^15^N-^1^H HSQC to investigate the secondary structure content of tropomodulin’s N-terminal IDR (Greenfield et al., 2005) and its binding site for tropomyosin (Greenfield et al., 2005; Kostyukova et al., 2007). 2D ^13^C-^13^C solid-state NMR analyses of $C_{\alpha }\text{/}C_{\beta }$ peaks of serine and alanine residues in the desmin head domain revealed that approximately 80% of serine and 40% alanine residues are in β-strand conformations, respectively (Zhou et al., 2021b), suggesting the presence of both structured and disordered elements (Zhou et al., 2021a). Applications of NMR to MyBPC3 via 2D ^1^H-^15^N HSQC show that its M-domain consists of an N-terminal IDR and a C-terminal folded subdomain (Howarth et al., 2012). Heteronuclear NOE values of each amide group and ^15^N T2 relaxation suggest that the linker in the M-domain of MyBPC3 is highly dynamic and disordered (Michie et al., 2016).

##### Other techniques.

There is an extensive collection of less-commonly used techniques for probing IDR structure. HDXMS is one such example that measures the extent to which a proton from a deuterated solvent is exchanged with those of the protein. Amino acids that are exposed to the solvent will have higher deuterated protein content than those that are well-buried and isolated from the solvent. Furthermore, highly mobile regions will exchange more rapidly than rigid regions (Fanning et al., 2018). The proton content is assessed via mass spectrometry and was recently used to characterize disordered linkers in titin (Zhou et al., 2021a).

AFM is commonly used to investigate the extensibility of proteins under a traction force. Unfolded proteins or IDRs exhibit more pliant force–length relationships relative to folded proteins. This technique has been applied to titin’s PEVK motifs (Linke et al., 2002) to discriminate between folded and unfolded regions, as well as MyBPC3 (Previs et al., 2016).

X-ray crystallography is used to probe well-folded proteins with unique conformations. While IDRs are generally not amenable to this technique, those that undergo unfolded to folded transitions upon binding a target can be resolved. An example application resolved the autoinhibited state of ankyrin, in which its IDR tail was folded (Chen et al., 2017). Similarly, X-ray crystallography was used to reveal the complex between α-actinin and FATZ-1 (myozenin-1) fragments that contain IDRs (Sponga et al., 2021). Lastly, for transglutaminase 2, X-ray crystallography was used to infer that this protein has several IDRs, based on missing structural information in the loop regions (Pinkas et al., 2007). Other less commonly used techniques include electron paramagnetic resonance, proteolytic degradation, and Fourier transform infrared spectroscopy (Uversky, 2020).

#### Computational approaches

Computational approaches are important tools that complement experiments in revealing molecular bases for IDR ensembles. Key computational approaches are described below.

##### Bioinformatics approaches.

An initial objective in IDR studies is to identify regions in protein sequence data that may be disordered. Significant advancements have been made in the area of IDP and IDR prediction from amino acid sequenc alone. IDPs feature unique sequence patterns including the enrichment of charged and polar residues relative to globular proteins. This impacts their ensemble compactness based on the charge distribution in the sequence (Das et al., 2015). They also tend to contain more serine, glycine, and proline residues (Lieutaud et al., 2016), of which the latter two impose different torsional constraints on the protein backbone relative to the other amino acids (Krieger et al., 2005). Additionally, IDRs tend to be depleted of aromatic residues (F, W, and Y) (Povarova et al., 2014) and some hydrophobic amino acids (I, L, and V) (Lieutaud et al., 2016). These sequence trends lend themselves to predicting the IDP propensity based on amino acid identities. Currently, there are tens of web servers or stand-alone tools for IDP prediction based on physicochemical properties, templates, metadata, and machine learning (Liu et al., 2019). Others focus on the detection of SLIMs important to binding IDRs to partnering proteins (Lindorff-Larsen and Kragelund, 2021). These tools have comparable performance, but can be dataset dependent (Liu et al., 2019).

There are also techniques for predicting structures for IDPs. An IDP’s ensemble can be explicitly generated from sequence via tools like flexible-meccano (Ozenne et al., 2012), which uses basic conformational potentials derived from coil statistics together with user-customized potentials. The tool directly generates IDP conformations which, with proper user-customized potentials, can match NMR and SAXS measured IDP ensemble properties. Qualitative attributes of IDPs structural states, such as compactness and coil/globule properties, can be estimated from its sequence via the IDP diagram from the Pappu lab (Holehouse et al., 2017). It is important to note that although being termed ‘intrinsically disordered’, IDPs commonly adopt partially folded structures. These residual structures can serve as molecular recognition fragments important to function (Gibbs and Showalter, 2015). However, predicting these residual structures is challenging as mainstream tools such as DSSP, STRIDE, and KAKSI show significant discrepancies (Zhang and Sagui, 2015).

Given their reasonable accuracy, inexpensive computational expense, and user-friendly features, IDP prediction tools are routinely used to gain insight into structural disorder in myofilament proteins, especially for those whose structural information is scarce. As one example, bioinformatics approaches have been used to predict the IDP regions of different TnI isoforms (Hoffman et al., 2006) and concluded that the N- and C-termini of TnI are intrinsically disordered across all isoforms. A similar bioinformatic method was applied to the intact Tn complex (TnI, TnC, and TnT) and disease-associated variants (Na et al., 2016). This study revealed that all three components have IDRs, even the well-folded TnC. Furthermore, the authors conclude that some disease-associated mutants reduce the disorder of the Tn complex, due to localized disorder-to-order transitions (Na et al., 2016). An additional example includes a study utilizing two predictors that reveal that the C-terminus of desmin is an IDR (Anbo et al., 2019). This IDR harbors the binding site for alphaB-crystallin (CRYAB) according to IDEAL (Intrinsically Disordered proteins with Extensive Annotations and Literature) annotations (Fukuchi et al., 2012; Anbo et al., 2019).

##### Implicit/semi-analytic representations.

Polymer theory can provide insights into IDP ensemble properties, such as the end-to-end distance probability $p\left(r\right)$. The most basic polymer model ignores interactions between monomers and assumes they are freely jointed (ideal chain model). This model yields quantities like the mean squared end-to-end distance, $\langle R^{2}\rangle$ and radius of gyration, $R_{\text{g}}$, as a function of the number of monomers (amino acids), N:

(3) $$\langle R^{2}\rangle =Nb^{2}$$

(4) $$\langle R_{\text{g}}^{2}\rangle =\frac{\langle R^{2}\rangle }{6}$$

where b, the Kuhn length, reflects the stiffness and local interactions. Other models can avoid overlapping monomers (worm-like chain (WLC) and excluded volume chain models) (Milstein and Meiners, 2013). The WLC uses the persistence length, ζ, which is approximately b/2, to give the mean squared end-to-end distance:

(5) $$\langle R^{2}\rangle =2\zeta L_{0}\left[1-\frac{\zeta }{L_{0}}\left(1-\text{exp}\left(-L_{0}/\zeta \right)\right)\right]$$

where $L_{0}=NL_{b}$ with $L_{b}$ and N representing the length of an amino acid and the number of amino acids, respectively. These models, when coupled with FRET experiments, are particularly useful for revealing chain compactness and dynamics of IDRs (reviewed in Schuler et al., 2016).

To account for intramolecular steric effects and interactions between monomers and solvents, the excluded volume concept was introduced in Zimm et al. (1953). This led to the Flory theory that quantifies competition between monomer/monomer and monomer/solvent interactions in determining compactness. This model accounts for excluded volume and entropic contributions in estimating the free energy of a polymer. It yields an approximation of the form:

(6) $$\langle R\rangle \approx N^{\nu }$$

where v is a scaling parameter (v=0.6 is common for disordered chains) (Grupi and Haas, 2011). Treatments based on Flory–Huggins theory have been used to fit radii of gyration as a function of a solution’s crowded volume fraction and the overlap of disordered chains (Soranno et al., 2014). Revised polymer models that explicitly account for charge distributions and monomer interactions, such as short-range repulsion and long-range electrostatics interactions, are also available to better describe IDP ensemble properties (reviewed in Ghosh et al., 2022).

Related to the concept of excluded volume is that of crowding, which refers to the mixing of a solute in a solution composed of solvent and other biomolecules. This is appropriate for the cell cytoplasm, which has a free volume fraction of roughly 0.78 (Kekenes-Huskey et al., 2016). Along these lines, a theoretical framework has been developed to predict the scaling of an IDP’s compactness $\left(\langle R_{g}\rangle \right)$ with the free volume fraction ϕ in the presence of hard sphere crowders (Kang et al., 2015):

(7) $$\langle R_{\text{g}}(\phi )\rangle =\langle R_{\text{g}}(0)\rangle f(x)$$

(8) $$x\approx \langle R_{\text{g}}(0)\rangle \text{/}{\left(4\pi \text{/}3\right)}^{1\text{/}3}\sigma_{\text{c}}\phi^{-1\text{/}3}$$

where $\langle R_{g}(0)\rangle$ is an IDR’s native compactness in crowder-free solutions and $\sigma_{\text{c}}$ is the radius of the crowder. While the exact solution of the scaling factor $f\left(x\right)$ is not defined exactly, it is bounded by x≾𝓞(1) and x≫𝓞(1), which correspond to a decreasing $\langle R_{g}\rangle$ and a coil-to-globule transition as ϕ increases, respectively.

Electrostatic interactions can also dramatically alter the size of a polymer. Here it is useful to define the electrostatic potential, $V_{ele}$, between two charged spheres representing residues i and j that are separated by distance $r_{ij}$ with point charges $q_{i}$ and $q_{j}$ via the Debye–Huckel model (Chu and Wang, 2019):

(9) $$V_{ele}=K_{coul}B(\kappa )\sum \frac{q_{i}q_{j}\text{exp}\left(-r_{ij}\kappa \right)}{\epsilon_{r}r_{ij}}$$

In this expression, $\kappa \equiv \lambda^{-1}$, where λ represents the Debye length, the distance over which electrostatic effects are most strongly screened by electrolytes. The Debye length is formally defined as $\lambda =\sqrt{\epsilon_{r}k_{\text{B}}T\text{/}\sum_{i}n_{i}^{0}q_{i}^{2}}$, for which the denominator reflects the solution’s ionic strength. B(κ) is an approximate constant for a dilute electrolyte concentration. While it is intuitive that like- and unlike-charged residues will repel and attract one another, it is evident from Eq. (9) that the ionic strength will dictate the strength of these interactions. Namely, high ionic strength attenuates electrostatic interactions, which thereby influences IDR compactness.

The IDRs predicted in Fig. 3 either cap the N- or C-terminus of a well-folded protein or link two well-folded domains. Here, polymer theoretic models can be used to predict an ensemble’s distribution about a folded protein or domain anchor. An example model from Van Valen et al. (2009) provides an analytic form for a binding domain linked to an anchor by an unstructured region. Here, the effective concentration, $c_{\text{eff}}$, is estimated via a Gaussian chain model (Van Valen et al., 2009):

(10) $$c_{\text{eff}}(R)={\left(\frac{3}{4\pi \xi L}\right)}^{3/2}\text{exp}\left(-\frac{3R^{2}}{4\xi L}\right)$$

where R is a user-provided distance between the polymer’s two ends and L is the linker length. The effective concentration of the untethered domain at a given R can be used to estimate its likelihood of binding a target (see section ‘Implicit and semi-analytic representations’ Eq. (56); Siddiqui et al., 2016).

##### Explicit representations.

*All-atom simulations* Molecular simulations are commonly used to sample IDP conformations constituting an ensemble. Simulation-generated conformations enable direct interpretation of an IDP’s structure–function relationships. Molecular simulation methods can be categorized into Monte Carlo and MD approaches. These forms of simulations are all based on a Hamiltonian, 𝓗, for estimating the energy associated with N particles, which is given by the sum of the system’s potentials and kinetic energies. These particles can be linked through bonding potentials. For example, the bonded potential of the Amber force field (Weiner et al., 1984) consists of three energy terms representing bond, angle, and dihedral terms:

(11) $$V_{bonded}=\sum_{i\in bonds}k_{b,i}{\left(l_{i}-l_{i,0}\right)}^{2}\;\;\;\;+\sum_{i\in angles}k_{a,i}{\left(\theta_{i}-\theta_{i,0}\right)}^{2}\;\;\;\;+\sum_{i\in torsions}\sum_{n}V_{n,i}\left[1+\cos\left(n\omega_{i}-\gamma_{i}\right)\right]$$

where $l_{i,0}$, $\theta_{i,0}$, and $\gamma_{i}$ are the bond length, angle value, and dihedral phase angle at equilibrium, $k_{b,i}$, $k_{a,i}$, $V_{n}$ are the force constants, and n in the torsion term refers to the multiplicity. The potentials associated with non-bonded interactions including van der Waals (vdW) and electrostatics are commonly given by:

(12) $$V_{non-bonded}=V_{ele}+V_{vdw}\;\;\;\;\;=\sum_{i}\sum_{j>i}\frac{q_{i}q_{j}}{\epsilon r_{ij}}\;\;\;\;\;+\sum_{i}\sum_{j>i}\frac{A_{ij}}{r_{ij}^{12}}-\frac{B_{ij}}{r_{ij}^{6}}$$

where $r_{ij}$ is the separation between atoms (beads) i and j, $q_{i}$, $q_{j}$, are point charges, and $A_{ij}$, $B_{ij}$ are vdW constants of atoms i and j.

The parameters for the expressions are defined in a force field. Force fields have generally been optimized for well-folded proteins, thus early applications to IDPs presented artifacts including overpredictions of compaction and secondary structure formation (Henriques et al., 2015). As simulations of IDPs matured, corrections to these force fields resulted in versions such as the Amber ff99SB-ILDN (Lindorff-Larsen et al., 2010) that mitigate these effects to a certain extent.

*Monte Carlo and lattice simulations* Monte Carlo (MC) algorithms utilize random displacements of molecular coordinates to identify the most thermodynamically favorable conformations. MC approaches define a Hamiltonian similar to those in Eq. (11) comprising bonded and non-bonded terms. At each iteration of the algorithm, a new system is generated from the current state (or initial state) by randomly displacing its coordinates. For the Metropolis MC algorithm, if the new system ${\left\{x\right\}}^{\prime }$ yields a total energy that is lower than that of the previous state $\left\{x\right\}$, the new state is stored as the current state. Otherwise, a new state is accepted if (Earl and Deem, 2008):

(13) $$N_{unif}(0,1)<p$$

(14) $$\text{with}\;p=\text{exp}\left(-\beta \left[U\left({\left\{x\right\}}^{\prime }\right)-U(\{x\})\right]\right)$$

where $U\left({\left\{x\right\}}^{\prime }\right)$ and U({x}) are the energies of the new and previous state, respectively, and $\beta =1\text{/}k_{B}\;T$ is the thermal energy of the system. The random displacements can be constrained to a uniformly spaced grid (lattice simulation) or assume continuous distributions. Metropolis MC simulations were recently used to show that the conformation ensemble of an 81 amino acid IDP transcription factor Ash1 from *Saccharomyces cerevisiae* in its native and phosphorylated had similar radii of gyration (Martin et al., 2016).

The simulation process accumulates an ensemble of conformations, e.g. $X=\left\{{\left\{x\right\}}_{1},{\left\{x\right\}}_{2},\ldots ,{\left\{x\right\}}_{n}\right\}$, which can be used to compute metrics such as an ensemble-averaged radius of gyration via:

(15) $$\langle R_{\text{g}}\rangle =\frac{1}{\left|X\right|}\sum_{n\in X}\sqrt{\frac{\sum_{i}{\left\|r_{i}\right\|}^{2}m_{i}}{\sum_{i}m_{i}}}$$

where $r_{i}$ and $m_{i}$ are the position and mass of atom i, respectively. Because there is an energy associated with each conformation from evaluating Eqs. (11) and (12) (above), one can compute ensemble-averaged, thermodynamic quantities such as the free energy, 〈G〉, using:

(16) $$\langle G\rangle =\frac{1}{\left|X\right|}\sum_{i}G\left({\left\{x\right\}}_{i}\right)$$

where $G\left({\left\{x\right\}}_{i}\right)$ is the free energy for each simulated conformation, such as from molecular mechanics calculations.

*Molecular dynamics (MD) simulations* MD entails minimization and displacement of atoms according to forces, based on Eqs. (11) and (12). Minimization techniques including steepest descent and conjugate gradients move particle positions according to the gradient of the potential energy surface (PES) until the system energy is minimized (often local):

(17) $${\left\{x\right\}}_{N^{\prime }}={\left\{x\right\}}_{N}-\gamma \nabla U\left({\left\{x\right\}}_{N}\right)$$

where ${\left\{x\right\}}_{N}^{\prime }$ and ${\left\{x\right\}}_{N}$ are the new and current configurations consisting of N particles, respectively. $\nabla U\left({\left\{x\right\}}_{N}\right)$ is the energy gradient at the current system’s configuration, and γ is the step size along the gradient. This is done in order to reconcile bond and non-bond energies that are incompatible with the force field. However, for IDRs, minimization is generally likely to yield a single, local minimum despite many states sharing similar energies.

Dynamic approaches temporally evolve a system by integrating Newton’s equations of motion for each atom:

(18) F({x})=−∇U({x})

(19) $$\frac{dv}{dt}=\frac{F(\{x\})}{m}$$

where $F\left(\left\{x\right\}\right)$ is the force acting on the system, v and m are the atom’s velocity and mass. During the simulation, v and $\left\{x\right\}$ are repeatedly updated at every time step δt:

(20) $$v_{t+\delta t}=v_{t}+\delta tF\left({\left\{x\right\}}_{t}\right)\text{/}m$$

(21) $${\left\{x\right\}}_{t+\delta }={\left\{x\right\}}_{t}+\delta tv_{t}$$

An initial guess for the system’s coordinates $\left(\{\vec{r}(0)\}\right)$ may come from the Protein Data Bank. Particle velocities, $v_{i}$, are randomly drawn from a Maxwell–Boltzmann velocity distribution to establish the system’s temperature:

(22) $$p\left(v_{i}\right)=\sqrt{\frac{m_{i}}{2\pi kT}}\text{exp}\left(-\frac{m_{i}v_{i}^{2}}{2k_{\text{B}}T}\right)$$

where k is Boltzmann’s constant, T is temperature, $m_{i}$ and $v_{i}$ are the mass and velocity of atoms i, respectively. Because the ensemble generated by MD is a function of time, this technique is frequently used to assess the kinetics of intra- and intermolecular interactions, which we later describe in sections ‘Computational methods for predicting intramolecular dynamics of MAPIDs’ and ‘Computational methods for predicting the MAPID co-assembly’.

Similar to MC simulations, MD simulations yield trajectories of conformations, $X=\left\{{\left\{x\right\}}_{1},\;{\left\{x\right\}}_{2},\;\ldots ,\;{\left\{x\right\}}_{n}\right\}$. MD simulations have been used to provide atomic resolution structural characterizations of IDRs in MAPIDs. For example, unbiased μs-long explicit MD simulations of the troponin complex with TnI IDRs were conducted to study its dynamics, stability, intramolecular interactions, and Ca^2+^ affinity (Cheng et al., 2014; Lindert et al., 2015; Zamora et al., 2016). The N-terminal IDR of TnI was shown to interact with TnC, whereupon the IDR’s phosphorylation at S23/S24 reduces TnC Ca^2+^ affinity (Cheng et al., 2014; Zamora et al., 2016).

*Brownian dynamics (BD) simulations* BD facilitates coarsening of the system spatial resolution to lengthen the simulation time steps and ultimately the duration of the simulation. This is done by describing the influence of explicit solvent molecules on the solute with a random force and a dissipative friction term. In this way, the solute alone can be simulated without solvent, which permits much larger simulation step sizes:

(23) $$mv^{\prime }=-\nabla U(\vec{r})-\gamma v+\sqrt{2\gamma k_{\text{B}}Tn(t)},\;n(t)=𝓝(0,1)$$

(24) $$v=-\nabla U(\vec{r})\text{/}\gamma +\sqrt{2\gamma k_{\text{B}}Tn(t)}\text{/}\gamma ,\;mv^{\prime }\to 0$$

where γ is a friction coefficient and 𝓝 is a Gaussian distribution of random variates with a mean of zero and variance of 1. The latter equation represents an overdamped system, for which the inertial terms are insignificant. As with MD, BD generates a trajectory comprising protein conformations at successive time points. BD has been used to reveal the ensemble properties of several naturally occurring IDRs as well as engineered (Glu-Lys)_25_ IDRs (Ahn et al., 2022). When coupled with an appropriate interaction potential, such as the coarse-grained force field for proteins (Ahn et al., 2022) and a Debye–Huckle model (Eq. (9)), molecular properties such as inter-residue distances, the IDP’s aggregation propensity, and ionic strength effects can be determined (Ahn et al., 2022). BD has also been widely used to explore both the intramolecular and intermolecular dynamics of IDRs, as will be discussed in sections ‘Computational methods for predicting intramolecular dynamics of MAPIDs’ and ‘Computational methods for predicting the MAPID co-assembly’, respectively. This technique has been applied to the myofilament proteins TnC and Tm. Specifically, BD was used to estimate Ca^2+^ binding rates to TnC (Lindert et al., 2012a) and to simulate the binding of Tm to actin (Aboelkassem et al., 2019).

*Coarse-grained (CG) simulations* CG represents another strategy of reducing a system’s degrees of freedom to improve sampling efficiency. In general, CG schema treat each residue as a single bead located at the $C_{\alpha }$ atom, or two beads with the second bead representing a residue’s side chain. One such CG scheme is the Martini model (Marrink et al., 2007) that provides an intermediate representation between CG and all-atom (AA). This is done primarily by representing large side chains by more than two beads. Compared to AA simulations, the degrees of freedom in CG are greatly reduced and thereby enable larger time steps than all-atom simulations.

CG molecular dynamics simulations have been used with IDPs (Ramis et al., 2019), such as for investigating IDP-aggregation or liquid–liquid phase transition (LLPS) phenomena (Benayad et al., 2021). LLPS describes the tendency of IDRs to form multi-valent PPIs with themselves or with other proteins. This aggregation can enable assemblies to phase separate from the surrounding solution (Harmon et al., 2017). It is believed that this phenomenon is used in biological systems to sequester biomolecules (Zhao and Zhang, 2020). To model this behavior, lattice and CG simulations have been proposed for describing phases of multi-valent protein assemblies (Harmon et al., 2017). Recently, Sponga *et al*. showed that in the sarcomere, FATZ-1 contains IDRs that condense into a liquid phase, which may provide a new mechanism to interact with α-actinin (Sponga et al., 2021). These models can benefit from coarse-graining the Hamiltonian to include only bond, Lennard-Jones, and bending potentials (Garaizar et al., 2020). In parallel, new force fields are also being developed to better align these simulations with experiments (Regy et al., 2021; Wohl et al., 2021).

*Enhanced sampling techniques* One of the most significant challenges in conducting simulations of IDRs arises from the proteins’ conformational diversity and expansive timescales to match experimentally measured ensemble-level properties. This challenge stems from two limitations of MD methods: (1) force field accuracy and (2) sampling efficiency. A variety of force fields have been developed to improve IDR simulation accuracy, such as the ff14IDPSFF (Song et al., 2017). Experimental constraints are also frequently introduced to restrict the conformational space sampled during simulation, thus focusing the search on biologically relevant states (see reviews Bhattacharya and Lin, 2019; Gong et al., 2021; Wang et al., 2021). Brute force, sub-ms length AAMD simulations have been performed to describe isolated IDP ensembles and binding mechanisms to partners (Robustelli et al., 2020; Chang et al., 2021). However, these simulations are either performed on specialized machines such as Anton (Robustelli et al., 2018) or require extensive computational resources. A number of enhanced sampling techniques have been developed (Gong et al., 2021) to reduce the computational expense with conventional computing resources.

**Fig. 7.**
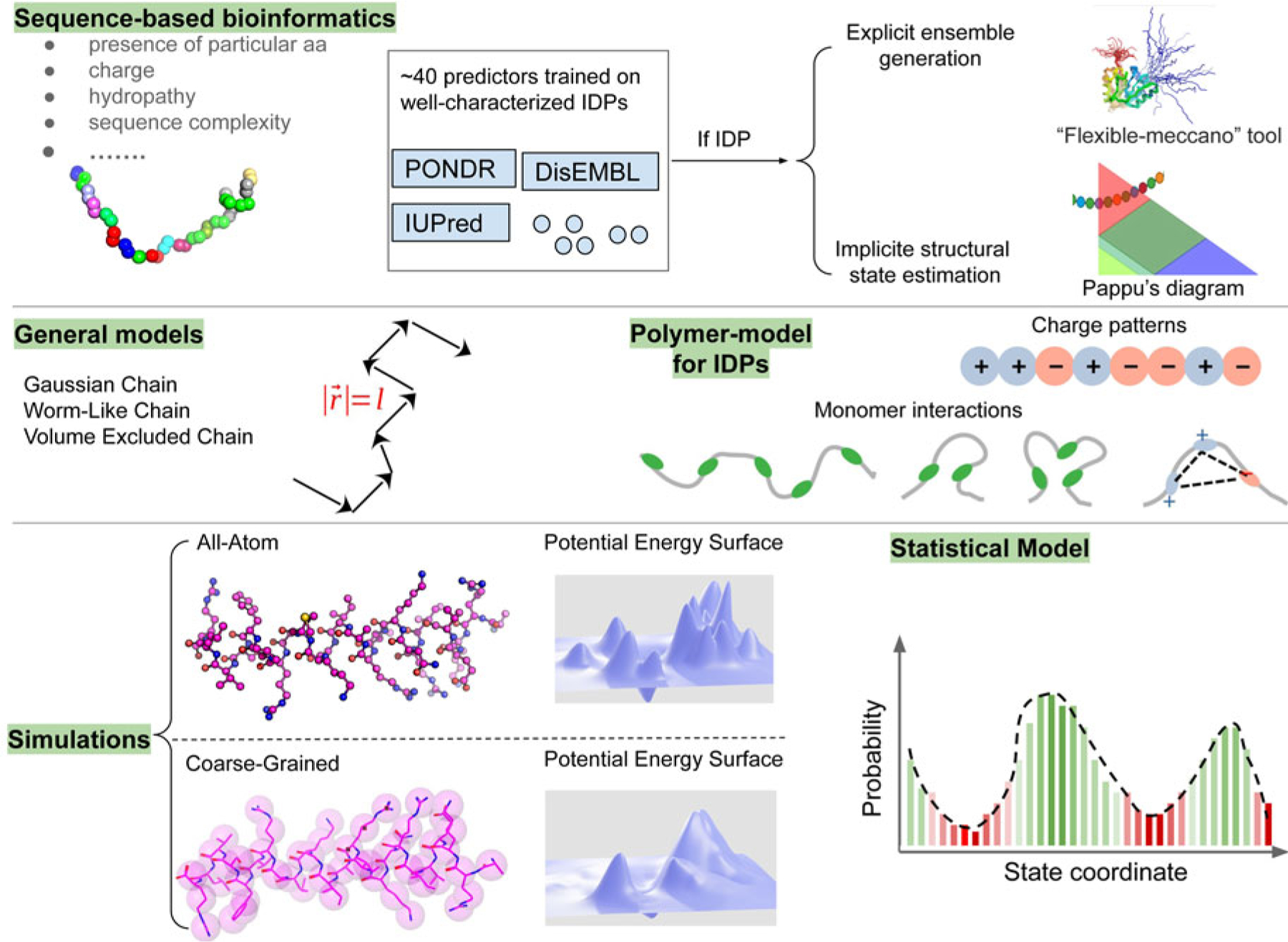
Computational methods commonly used to model IDR structural properties. The IDR propensity of a structure can be predicted from its amino acid sequence by tens of established tools (Liu *et al*., 2019), and structural states of IDP can be either explicitly modeled (Ozenne *et al*., 2012) or characterized by an implicit phase diagram (Holehouse *et al*., 2017). Polymer models including the simplistic freely jointed monomer model and advanced models that account for intramonomer interactions can be used to characterize IDR ensembles (Milstein and Meiners, 2013; Schuler *et al*., 2016). Particle-based simulations are commonly used to predict IDR conformer structures and associated kinetics (Wang, 2021). All atom simulations consider every individual atom in the system to determine detailed descriptions of the potential energy surface (PES) but are computationally intensive, while coarse-grained simulations lump atoms together to increase sampling efficiency at a modest loss of accuracy. Lastly, statistical models frequently use partition functions to obtain thermodynamic descriptions of IDRs (Hadzi *et al*., 2021).

*Temperature-based methods.* Elevating a system’s temperature increases the likelihood of the system to sample minima that are separated by large PES barriers (see Fig. 7). Replica exchange MD simulations exploit this principle by simultaneously running multiple replicas of the system at different target temperatures. During the simulations, states of neighboring replicas are exchanged at constant intervals, with the exchange probability defined as:

(25) $$\begin{array}{l} p\left({\left\{x\right\}}_{i}\right)↔\left({\left\{x\right\}}_{j}\right)\\ \;\;\;=\min\left(1,\text{exp}\left[\left(\frac{1}{k_{\text{B}}T_{i}}-\frac{1}{k_{\text{B}}T_{j}}\right)\left(U\left({\left\{x\right\}}_{i}\right)-U\left({\left\{x\right\}}_{j}\right)\right)\right]\right) \end{array}$$

where $T_{i}$ and $T_{j}$ are the target temperatures, at which the replicas i and j are simulated. The number of replicas and the target temperatures are determined by the number of particles to set the exchange probability to roughly p≈0.4 (Zhang et al., 2005). Temperature replica exchange MD has proven to be reliable in its many applications to both folded proteins and IDPs (Wu et al., 2009; Wang et al., 2020). Many variations of replica exchange techniques, such as the Hamiltonian replica exchange MD (HREMD), replica exchange with hybrid tempering (Appadurai et al., 2021), and Hamiltonian scaling in replica exchange with solute tempering (REST) (Liu et al., 2005) have been developed. For these variants, the main concept is to simulate only a subset of atoms to be at different temperatures, thus focusing sampling on the subsystem of interest. Applying these techniques can greatly shorten the simulation time for IDP studies. A recent example of this technique used unbiased all-atom molecular dynamics (AAMD) with HREMD to model the c-Src kinase N-terminal IDR ensemble over a 1 μs timescale (Shrestha et al., 2019).

##### Modified potential.

In complement to using elevated temperatures to cross PES barriers, reducing barrier heights by altering the potential can facilitate sampling. Biased potentials can be applied to user-defined collective variables (CVs) to enhance sampling. Adding boost potentials that raise the energy minima of a PES accelerates sampling by effectively lowering the energy barriers. Metadynamics is one such method that has been widely used to explore the conformational space of biomolecules and for determining free energy landscapes. In metadynamics, a biased potential with a Gaussian form is applied to a user-selected CV at a constant interval τ, which results in the potential (Laio and Parrinello, 2002):

(26) $$V(\vec{s},t)=\sum_{n\tau }W(n\tau <t)\text{exp}\left(-\sum_{i}^{d}\frac{{\left(s_{i}-s_{i}^{\left(0\right)}(n\tau )\right)}^{2}}{2\sigma_{i}^{2}}\right)$$

where W(nτ) and $\sigma_{i}$ are the height and width of a bias potential, respectively. $s_{i}$ is the targeted state of the CV and $s_{i}^{\left(0\right)}(n\tau )$ is its instantaneous value at time nτ. The advantage of metadynamics is that the sum of the added bias potentials over the simulation time course approximate the free energy profile along the CV:

(27) $$V(\vec{s})=-G(\vec{s})$$

In practice, however, the values of W, τ, and σ need to be carefully chosen for good performance.

The umbrella sampling method is a popular biased-sampling technique that enhances the sampling at windows along a CV by introducing a harmonic potential centered at each window’s midpoint. The sampling of CVs along the windows is then used to recover the unbiased potential of mean force (PMF). A prominent challenge for the CV-based accelerating simulation technique is the choice of CVs. Choosing the appropriate CV that can capture a protein’s biological-relevant degrees of freedom is challenging (Chen, 2021). Bias-exchange metadynamics (BE-MTD) alleviates this problem by performing metadynamics simulations with different CVs to identify the optimal sampling path (Piana and Laio, 2007). In BE-MTD, a metadynamics replica is performed for each CV, and the exchange of conformations from randomly paired replicas occurs at a constant time-interval with a Metropolis acceptance criterion (Eq. (13)). Lastly, machine learning techniques have also been trained to find optimal CVs (Chen, 2021). For example, the autoencoder model, which contains an encoder to map high-dimensional data $\left(\left\{x\right\}\right)$ to low-dimensional data $\left(\vec{s},\;\text{CV}\right)$ and a decoder to reverse the process, can be trained based on MD sampled configurations to identify an appropriate CV (Chen, 2021).

Biased potentials can also be applied to broader sets of coordinates beyond CVs. Accelerated and Gaussian-accelerated MD are two related methods that are similar to metadynamics, but require no prior knowledge of CVs (Hamelberg et al., 2004; Miao et al., 2015). For Gaussian accelerated MD, the boost potential is applied system-wide to a protein’s total potential, or the dihedral space (Miao et al., 2015), instead of user-selected CV, when the system’s original potential is smaller than a threshold:

(28) $$U(\vec{r})=U_{0}(\vec{r})+\Delta U(\vec{r}),\;\text{if}\;U_{0}(\vec{r})\leq E_{thres}$$

For this approach, the standard deviation of the boost potential $\sigma_{\Delta U(\vec{r})}$ is recommended to be less than $10k_{\text{B}}T$ to ensure accurate reweighting for recovering the unbiased PES (Miao et al., 2015).

These categories of enhanced sampling techniques can be combined to improve predictions of an IDR ensemble. For example, in Li et al. (2022), replica-exchange and metadynamics were combined to reveal ensemble properties of a 46 amino acid IDR derived from the DEAD-box protein DHH1. By using the advanced sampling techniques, the authors determined the free energy landscape as a function of secondary structure content and determined that the IDP has a low propensity for self-aggregation (Li et al., 2022). Lastly, while most of these strategies have been developed for explicit, AAMD simulations, in principle they can be adapted to any number of molecular simulation approaches.

*Statistical mechanics models* Statistical mechanics provides a framework to relate probabilistic representations of molecules to classical thermodynamics terms like the free energy. This is generally done by writing a partition function that enumerates the states available to a molecular system and their corresponding energies:

(29) $$\Omega (\beta )=\sum_{x_{i}}\text{exp}\left(-\beta 𝓗\left(x_{1},x_{2},\ldots \right)\right),$$

where 𝓗 represents an appropriate Hamiltonian for the system configuration (canonical) of interest. This partition function provides the appropriate normalization to determine the probability of a given state:

(30) $$P\left(x_{i}\right)=\frac{\text{exp}\left[-\beta 𝓗\left(x_{i}\right)\right]}{\Omega (\beta )}$$

Standard thermodynamic relationships such as the free energy can then be defined with respect to the partition function:

(31) $$G=-k_{B}T\;\ln|\Omega |$$

As such, they are most frequently used for systems in equilibrium.

Hadzi *et al*. proposed a statistical thermodynamics model to describe the fuzzy ensemble of IDPs when bound to targets (Hadzi et al., 2021). The core idea is that target-bound IDPs adopt heterogeneous structural states similar to the isolated state, but with higher fractions of α-helical content. Thus, in its bound state, an IDP’s ensemble properties are governed by a coil-to-helix transition propensity, and constraints on this transition imposed by interactions with the target. In that coil–helix transition model, each residue in the polypeptide can adopt either a coil state or helix state. Additionally, a subset of these residues are ‘hot spots’ that interact with the target. This yields a partition function of the form:

(32) $$\Omega =\sum_{2^{N}H-1}^{h=1}\Omega_{b,h}$$

where $\Omega_{b,h}$ is a partition function for a set of hotspots $\left(h\right)$ and $N_{\text{H}}$ is the total number of hotspots. $\Omega_{b,h}$ in turn is based on a generating function that gives the statistical weight for a residue’s propensity to undergo a coil-to-helix transition and a change in statistical weight based on its interaction energy, $\Delta G_{int}$, when bound with the target. These interaction energies can be obtained by MD simulations. This statistical model successfully predicted the changes of IDP helical content and binding affinity in two IDP–protein complexes driven by mutations in the IDPs, which were validated by NMR, CD, and isothermal titration calorimetry (ITC) binding assay data (Hadzi et al., 2021).

##### Solvation models.

Solvation is an important factor to consider when simulating an IDR ensemble, given that amino acid interactions with an aqueous solvent are strongly thermodynamically favorable for IDRs. The unstructured ensembles and dynamics of IDRs are more sensitive to protein/water interactions, compared to their folded counterparts (Wuttke et al., 2014). For this reason, current solvent models pose difficulties in accurately recapitulating experimentally determined IDR ensembles via simulation. This was recently illustrated in a study suggesting that the recently developed ‘general-purpose’ optimal opint charge (OPC) water model (Izadi et al., 2014) still yields predictions of IDP ensembles that deviate from experiment, despite its improved performance over standard transferable intermolecular potential with 3 points (TIP3P) models (Shabane et al., 2019). Efforts aiming to improve all-atom and CG solvation models for IDRs can be categorized into those improving the water model parameters alone or correcting the water/protein/ion interaction potentials. For example, refitting of the point charges in the TIP3P water model was reported to yield more accurately predicted IDP ensembles when validated against SAXS profiles (de Souza et al., 2020). In addition, Gil Pineda *et al*. used a modified TIP3P water model combined with the CHARMM36m protein force field to correct a tendency for simulations to over-predict the compactness of IDPs (Gil Pineda et al., 2020). Similarly, it was found that increasing the Martini forcefield’s protein–solvent interaction potential greatly improved the agreement between sampled IDP ensembles with data from SAXS and NMR (Thomasen et al., 2022). In tandem, optimization of metal ion parameters for different water models has been pursued toward reproducing experimentally determined hydration energies, coordination numbers, and ion-coordinated water exchange phenomena (Grotz and Schwierz, 2022). Lastly, there has been progress in adding energy terms to account for temperature-dependent protein stabilities into existing implicit solvent models to improve predictions for both IDP and globular proteins (Arsiccio et al., 2022).

##### Multi-scale simulations.

In contrast to ‘single-model’ approaches, other studies of IDRs have combined methodologies to enrich the diversity of sampling. As an example, a chain growth model has been combined with all-atom simulations to explore an IDP’s ensemble (Pietrek et al., 2020). Pietrek *et al*. used AAMD simulations, with a local-to-global step-wise divide-and-conquer strategy to achieve comprehensive ensemble sampling of the α-synuclein IDP. This entailed first sampling the local fragments independently, assembling the fragments with a ‘chain-growth Monte Carlo’ strategy, and then simulating the intact IDP as a final step (Pietrek et al., 2020). This process has been improved by incorporating experimental chemical shift data into the fragment assembly (Stelzl et al., 2022). Lastly, CG MD and AAMD can be combined, as shown by Garcia *et al*., to demonstrate the conformational space of a full-length IDP (Chvez-Garca et al., 2022).

##### Bridging computer models with experiments.

Simulations in section ‘Computational approaches’ are parameterized, validated, and refined using the experimental techniques listed in section ‘Experimental techniques’. We now discuss how comparisons between experimental data are made and how experimental data are used to constrain searches.

A variety of strategies have been proposed for aligning modeling results with, and validation against, experimental data. In principle, experimental data (such as FRET efficiency) and computational data (such as residue–residue distance) should yield similar distances between probed amino acids. However, in practice there may be limited overlap because of the intrinsically different spatiotemporal scales, at which these data are collected (Best, 2017) and measurement uncertainties. However, there are strategies that mitigate these comparisons, such as for co-aligning with NMR (order parameters (Lipari et al., 1982)), SAXS (radius of gyration (Henriques et al., 2015)), and fluorescence (pairwise distance (Metskas and Rhoades, 2015)). One such example includes a study that combined SAXS and NMR with the structure generation method Flexible-Meccano to characterize the ensemble of an IDP from the Sendai virus (Bernadó et al., 2005). In that study, the conformations of the IDP were iteratively generated via the ‘Flexible-Meccano’ modeling method, and the RDC and SAXS data predicted from these conformations were then compared against experimental values until convergence (Bernadó et al., 2005).

Similar approaches have been applied to the MAPIDs TnI, MyBPC3, and myotilin. Metskas *et al*. studied the IDRs in TnI, using MD simulations to probe the distances between multiple positions of TnI relative to TnC. These distances were compared against experimental FRET data (Metskas and Rhoades, 2015). Since the FRET observable time resolution (∼1 ms) was orders of magnitude longer than the protein’s conformational sampling (ns to μs), the experimental observable was reflective of the protein’s average dynamics. Toward that end, the authors used short MD replicas in parallel, starting from different initial structures to capture the protein’s conformational dynamics for direct comparison against FRET efficiency (Metskas and Rhoades, 2015). Michie et al. (2016) used a similar strategy to identify a linker in the M-domain of MyBPC3 for binding calmodulin via SAXS scattering and computational modeling. Another excellent MAPID study that utilized both experiment and simulation was performed by Kostan et al. (2021). Conformations comprising myotilin’s ensemble were selected from a pool of structures generated by the ‘EOM’ approach (Bernadó et al., 2007) to match experimental SAXS profiles. These conformations, together with biochemical binding assays, provide an integrative structural model that rationalizes the mechanism, by which myotilin’s IDR binds to F-actin (Kostan et al., 2021).

Other approaches have used Bayesian inference to relate predicted structural models to experimental data. With this framework, the conditional probability of simulating a protein conformation $y_{x}$, given an experimental observation, $\theta_{R\text{g}}$, is determined from Bayes theorem. This observation, for instance, could be a value of $R_{\text{g}}$ implied from SAXS data. This probability, $P(y_{x}|\theta_{R\text{g}})$, is known as the posterior distribution. The posterior is determined from the prior distribution, $P(y_{x})$, and the likelihood, $P(\theta_{R\text{g}}|y_{x})$. The prior distribution is the probability that the simulated protein yields a conformation $y_{x}$. The likelihood is the probability of making an experimental observation $\theta_{R\text{g}}$, given the simulated conformation $y_{x}$. These relationships culminate in the equalities:

(33) $$P\left(y_{x}|\theta_{R\text{g}}\right)=\frac{P\left(\theta_{R\text{g}}|y_{x}\right)P\left(y_{x}\right)}{P\left(\theta_{R\text{g}}\right)}$$

(34) $$=\frac{P\left(\theta_{R\text{g}}|y_{x}\right)P\left(y_{x}\right)}{\int_{y}P\left(\theta_{R\text{g}}|y_{x}\right)P\left(y_{x}\right)dy}$$

The denominator in Eq. (33) represents the probability of experimentally observing the value $\theta_{R\text{g}}$, and can be estimated from Eq. (34).

A study from Fisher *et al*. proposed a variation of this approach to determine population weights $\vec{\left(w(y\right)})$ of simulated protein structures that are consistent with a set of experimental measurements $\left(\vec{\theta }\right)$, using a posterior $P\vec{\left(w(y\right)}|\vec{\theta })$. The population weights were set by the Boltzmann factor (Eq. (30)), using energies calculated for all conformations. The prior $P(\vec{w})$ was based on Gaussian distributions using transformed representations of the weights. Importantly, the likelihood used the expected value of an experimental observable like $R_{\text{g}}$ from the weighted simulated structures, $𝓔\left(\theta_{i}|\vec{w}\right)$, as well as the error in the predicted observable, $\epsilon_{R\text{g}}$:

(35) $$P\left(\theta_{i}|\vec{w}\right)=\frac{1}{\sqrt{2\pi \epsilon_{R\text{g}}}}\text{exp}\left[-\frac{{\left(\theta_{i}-𝓔\left(\theta_{i}|\vec{w}\right)\right)}^{2}}{2\epsilon_{R\text{g}}}\right]$$

In practice, the authors generated an ensemble of protein conformations via MD simulations, after which their Bayesian framework was used to determine the most likely conformation weights that agreed with RDCs or chemical shifts from NMR (Fisher et al., 2010). Another variation of this approach defined an error function for SAXS data to penalize overfitting due to extraneous degrees of freedom (Bowerman et al., 2019). An example application to MAPIDs includes a study of MyBPC3, in which the Bayes inference is used to align simulation results with SAXS and NMR data. This strategy revealed that the flexible linker connecting the M-domain and C2 domain allows the two domains to sample diverse interdomain orientations, thus imparting considerable flexibility to the construct (Potrzebowski et al., 2018).

### Computational methods for predicting intramolecular dynamics of MAPIDs

#### Problem and application

The kinetics with which conformations exchange with one another are equally important relative to the breadth of conformations adopted by an IDP, e.g.

$${\left\{x\right\}}_{1}\underset{{\text{k}}_{21}}{\overset{{\text{k}}_{12}}{⇌}}{\left\{x\right\}}_{2}$$

where ${\left\{x\right\}}_{i}$ refers to the conformation i. This information is critical for understanding the timescale of MAPID functions relative to other physiological phenomena.

**Fig. 8.**
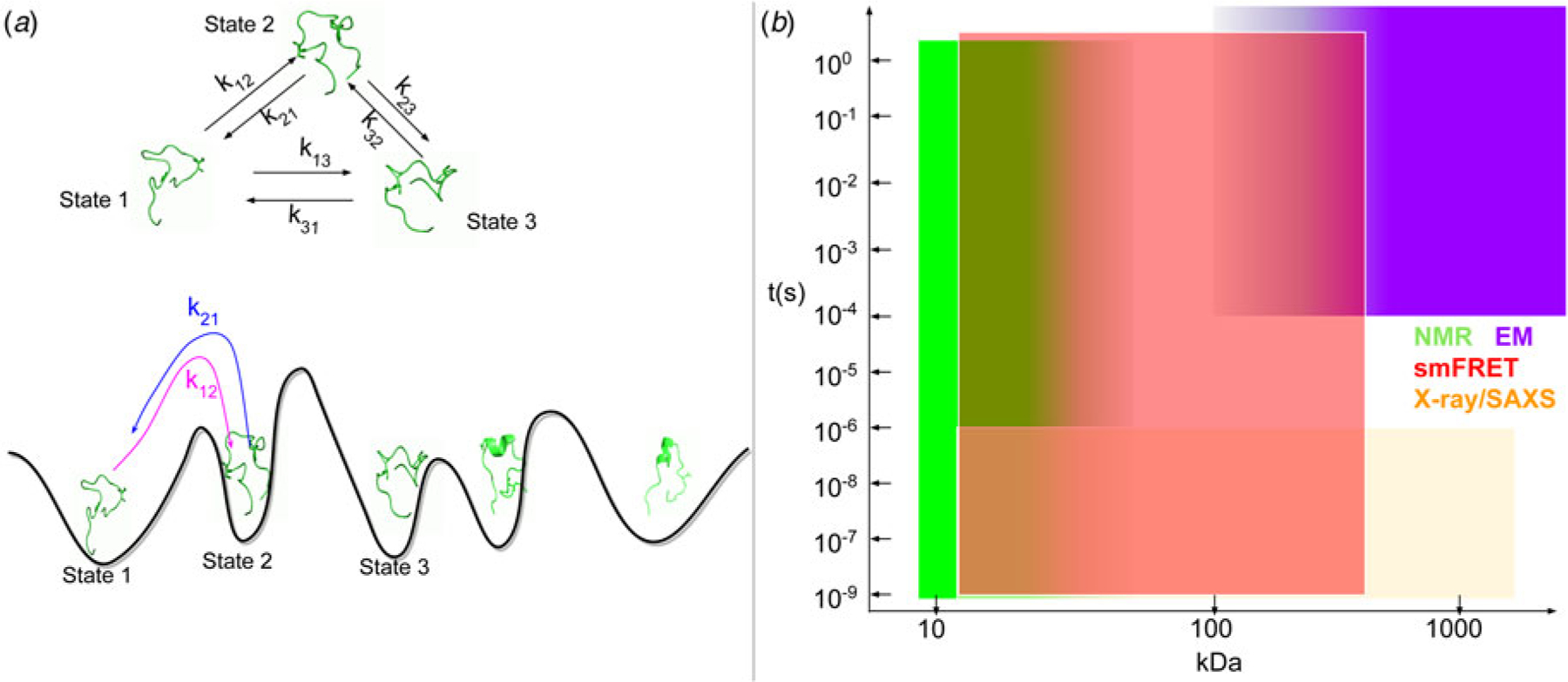
(*a*) Dynamics of an IDP ensemble represented by a Markov state model (MSM). (*b*) Experimental methods for structure determination and their temporal resolutions. Timescale information of NMR, X-ray, cryo-EM, and SAXS are taken from Ban (2020). FRET time resolution spans ns to seconds (Okamoto and Sako, 2017). Size information: EM is appropriate for proteins >100 kD (Yeates *et al*., 2020). NMR is most suitable for <30 kD proteins (Xu *et al*., 2006). X-ray can solve structures up to 4000 kD (PDB website statistics). FRET is used for medium-sized proteins but has been reported for up to a 540 kD protein (Sielaff *et al*., 2022).

As an example, the TnI switch peptide is disordered at resting (diastolic) Ca^2+^ levels. Binding of this peptide to TnC’s hydrophobic patch promotes a disorder-to-order transition of the peptide to form a folded α-helix in response to elevated cytosolic Ca^2+^ (Metskas and Rhoades, 2016). The timescale for this conformational search and fold process must occur within the brief rise in calcium that accompanies a typical heartbeat. For instance, the breadth of the TnI C-terminal IDR ensemble determines its effective concentration for steady-state measurements (section ‘Implicit/semi-analytic representations’). However, it is the rate at which the IDR samples the appropriate position and conformation to bind TnC, relative to the duration of elevated calcium, that will determine the rate of activating the thin filament. Similarly, the intrinsic timescales for myosin’s dynamic IDR loops to interact with actin could impact the rate of cross-bridge formation (Gurel et al., 2017; Doran et al., 2020). Altogether, the dynamics of these IDRs and those of other MAPIDs collectively influence the kinetics of force generation (Fig. 8).

#### Experimental techniques

##### NMR.

Probes of IDR conformational dynamics can be resolved via NMR experiments through analyzing spin relaxation data (Ban et al., 2017). For instance, residue mobilities and interactions can be obtained by fitting longitudinal (R1)/transverse (R2) relaxation rates and heteronuclear steady-state NOEs (Sibille and Bernado, 2012). Such techniques provide high structural resolution, although timescales can be limited to μs-ms for R1/R2 relaxations and ps-ns for NOEs (Palmer, 2004). RDCs can also be used to probe protein dynamics at atomic resolution in the ps to ms ranges (Tolman and Ruan, 2006). As an example, N^15^ relaxation provides ps to ns time resolution (Hwang et al., 2014), though interconversion of IDP conformations may approach timescales of μs or longer (Bernetti et al., 2017). Abyzov *et al*. also demonstrated that temperature-dependent NMR can yield ‘local’ activation energies for dynamic modes of an IDR ensemble (Abyzov et al., 2016). NMR relaxation techniques have also been used with TnI. Hwang *et al*. as an example assessed the dynamics of TnI’s N-terminus (residues M1–K37) and found that it remains intrinsically disordered even after binding to TnC (Hwang et al., 2014). In addition, relaxation rate constants and NOEs collected for MyBPC3 indicated that the linker spanning its tri-helix bundle and C2 domain exchanges conformations on a ps to μs timescale (Michie et al., 2016), that is much faster than the typical heart beat.

##### Time-resolved X-ray and SAXS.

Time-resolved X-ray crystallography enables observations of how electron density maps of protein domains evolve in time, which can provide functional insights (Schotte et al., 2003). While applications to MAPIDs have not yet been reported in the literature, the technique was used with myoglobin to reveal a series of structural intermediates exchanging on a 150 ps timescale during its functional cycle (Schotte et al., 2003). Time-resolved X-ray scattering was also applied to resolve quaternary structure dynamics on a 316 ns to 100 μs range for carboxyhemoglobin (Cho et al., 2021). In addition, SAXS has been used to complement NMR studies. In this context, SAXS can provide the general shape of an IDR ensemble, while NMR is used to reveal structural dynamics at ps–ns, μs, and ms timescales (Sibille and Bernado, 2012; Schneider et al., 2019). For example, the combination of SAXS and NMR enabled both conformational and dynamic characterization of IDRs in the ribosomal L12 protein (Sibille and Bernado, 2012), for which not only the ensemble of structures, but also their conformational transition timescales are obtained.

##### Fluorescence techniques.

Fluorescence-based techniques such as time-resolved fluorescence anisotropy are powerful tools for resolving protein motions occurring at different timescales. Time-resolved fluorescence anisotropy is well-suited for monitoring local backbone rotation and long-range correlation kinetics (Das et al., 2021). One example from Grupi and Haas (2011) introduced fluorescent labels at different positions of the α-synuclein IDP. Time-resolved FRET was then used to determine the α-synuclein end-to-end distance distributions, which revealed the disordered nature of the protein, as well as its rapid intrachain diffusion dynamics (Grupi and Haas 2011). Another example revealed TnT’s flexible linker conformations on the full cardiac thin filament. The time-resolved FRET data measured from labels in the IDR linker were then used as constraints to bias MD sampling of the TnT structure (Deranek et al., 2022). Fluorescence correlation spectroscopy is another technique that can be used to probe conformational changes occurring over 20–300 ns (Lee et al., 2015), but applications to myofilament proteins appear to be limited to determining protein concentrations *in vivo,* such as by Duggal et al. (2017).

#### Computational approaches

##### Implicit, semi-analytical approaches.

Examples of implicit, semianalytical methods for intramolecular IDP kinetics are fewer in number relative to the structural models introduced in the previous section. One model of note gives the rate, k, of two ends of a polymer to contact one another (Grupi and Haas, 2011):

(36) $$\frac{1}{k}=\frac{R^{2}}{3D}\left(\frac{\sqrt{\pi }}{2\alpha }+\ln2-1-\frac{\sqrt{\pi }}{2}\alpha +\frac{4}{3}\alpha^{2}\right)$$

where D is the intramolecular end-to-end diffusion coefficient, $R=\sqrt{\langle r^{2}\rangle }$ is the root mean square distance between the interacting residues, a is the radius of the residue, $\alpha ={\left(3\text{/}2\right)}^{0.5}\left(a\text{/}R\right)$, and D is the intramolecular diffusion coefficient. Similarly, there have been efforts to determine the kinetics of forming interior loops in polypeptide chains. For instance, loop closure kinetics for a WLC can be estimated if the PMFs are known in advance (Hyeon and Thirumalai, 2006).

##### Explicit simulations of intramolecular IDR kinetics.

Explicit models of IDP kinetics are common. Early approaches relied on explicit definitions of reaction coordinates (RCs), which are coordinates that describe the progress of a system exchanging between two states, such as dynamic changes in end-to-end distances (Doucet et al., 2007). Relating these dynamic changes to kinetics has been done using approaches based on the Smoluchowski equation (Hamelberg et al., 2005) to estimate dynamics along a one-dimensional PMF (Szabo et al., 1980; Hamelberg et al., 2005):

(37) $$\frac{\partial p(x,t)}{\partial t}=\frac{\partial }{\partial x}\left[D(x){\text{exp}}^{-\beta U(x)}\frac{\partial }{\partial x}\left({\text{exp}}^{\beta U(x)}p\right)\right]$$

where $p\left(x,\;t\right)$ is the time-dependent probability density along the RC, D is the diffusion coefficient, and $U\left(x\right)$ is the PMF. Other approaches include using Kramer’s rule (Berezhkovskii and Szabo, 2005) to calculate the transition rate (Berezhkovskii and Szabo, 2005):

(38) $$k={\left[\left(\int_{x_{b}-\Delta x}^{x_{b}+\Delta x}{\text{exp}}^{-\beta U(x)}dx\right)\left(\int_{x_{T}-\Delta x}^{x_{T}+\Delta x}\frac{{\text{exp}}^{\beta U(x)}}{D(x)}dx\right)\right]}^{-1}$$

where x is the RC, while $x_{b}$ and $x_{\text{T}}$ represent the initial state and transition state, respectively. Hyeon *et al*. also used Kramer’s rule to predict passage times over potentials of mean force based on the equilibrium distributions of the chains (Hyeon and Thirumalai, 2006).

These approaches rely on estimates of $D\left(s\right)$, which can be determined via time-resolved FRET (Grupi and Haas, 2011), or by using methods we discuss below. In many cases, a single (spatially uniform) diffusion coefficient suffices. This can be obtained via experiment or simulation by estimating a particle’s mean square displacements, $\langle x^{2}\rangle$, which are related to the diffusion coefficient in one dimension:

(39) $$\underset{t\to \infty }{\lim}\;\langle {\left(x(t)-x(0)\right)}^{2}\rangle =2Dt$$

(40) $$\Rightarrow \langle x^{2}\rangle =2Dt$$

The degree of disorder in an IDR can also be used to refine this constant (Chu and Wang, 2019). Other situations, especially those that use D(x) in parallel with potentials of mean force, benefit from estimates that are spatially dependent. In these cases, autocorrelation functions of a solute’s positions or velocities from explicit simulations can be helpful to obtain spatially resolved diffusion coefficients (Hummer, 2005) along a PMF and its barriers. Here one can rely on the principle that the autocorrelation function for Markovian processes decays exponentially over time with a correlation time $\tau_{c}$:

(41) $$\langle x(0)x(t)\rangle =\langle x^{2}\rangle \text{exp}\left(-t\text{/}\tau_{c}\right)$$

(42) $$\tau_{c}=\frac{1}{\langle x^{2}\rangle }\int_{0}^{\infty }\langle x(0)x(t)\rangle dt$$

The correlation time is related to the particle’s friction coefficient, ζ, and mass via $\tau_{c}=m\text{/}\zeta$. The Einstein relation $D=k_{\text{B}}T\text{/}\zeta$ then yields the diffusion coefficient from ζ. A derivation using harmonic forces to constrain the system along the PMF is provided in the appendix of Pace et al. (2021).

A key concern with such approaches is that force fields are generally optimized for equilibrium structures, not their dynamics, therefore the kinetic barriers to conformational motions may be poorly described (Ponder and Case, 2003). In addition, states with large energy barriers are much less frequently sampled or even inaccessible for conventional MD simulations, which could pose difficulties in identifying the most probable RCs. In general, RCs can be difficult to define or may consist of many parallel pathways (Hinczewski et al., 2010).

To partially address these limitations, enhanced sampling techniques have been used to sample regions that are separated by large energy barriers. For instance, accelerated MD simulations were reweighted and used Kramer’s rule (Eq. (38)) to estimate the unbiased kinetics of intramolecular transitions between conformations (Hamelberg et al., 2005). More recently, in Bernetti et al. (2017) metadynamics was used to reveal the PES of an IDP. Using a post-processing analysis called ‘Bin-Based Kinetic Model’, rate constants were recovered from the biased metadynamics trajectories.

*Markov techniques* A shortcoming of the preceding approaches is that an RC is needed to be defined *a priori*. Markov state modeling techniques (such as msmbuilder (Harrigan et al., 2017) and pyemma (Scherer et al., 2015)) bridge this gap by allowing the RCs to be directly determined from the MD simulation data. In brief, long simulations are performed to yield trajectories that span the conformational space of the protein. Then, the conformation ensembles from these simulations are discretized into microstates, based on a user-defined metric like RMSD (Prinz et al., 2011; Husic and Pande, 2018), from which rate constants for exchanging between microstates are determined. This approach is based on the idea that the timedependent change in the probability of a given state can be described as a rate of transition out of (or into) the state:

(43) $$\frac{dp_{j}}{dt}=-p_{j}\sum_{i\neq j}k_{ji}+\sum_{i\neq j}k_{ij}p_{i}$$

or more generally, for multiple states:

(44) $$\frac{dp(t)}{dt}=K(\delta t)p(t)$$

The relationship:

(45) $$p(t)=\text{exp}(Kt)p_{0}$$

can then be used to determine the state probabilities at time t.

If K from Eq. (44) is not known, which is generally the case for Markov state model (MSM) approaches, the change in probabilities can be expressed in terms of a transition probability matrix, T. This matrix evolves the state probabilities by some δt:

(46) p(t+δt)=T(δt)p(t)

The transition matrix is populated by counting the transition events from microstate i to j occurring within δt (the lag time), as calculated from the discretized trajectory. If the model is Markovian for n consecutive lag time periods, the following holds:

(47) $$p(t+n\delta t)=T^{n}(\delta t)p(t);$$

that is, as n→∞, the steady-state distribution $\left(p_{0}\right)$ is obtained. For systems that behave as Markovian processes (namely the transition probability is only dependent on the current state and is independent of previous states), the implied timescale of the mth eigenvalue, $t_{m}$ becomes independent of the lag time δt:

(48) $$t_{m}=-\frac{\delta t}{\ln\lambda_{m}}$$

where $\lambda_{m}$ is the mth eigenvalue of the transition probability matrix T. In practice, this implied timescale $t_{m}$ is used to validate that the model built from simulations is Markovian. Transition matrices from this approach can then be used to find K by recognizing that (Polizzi et al., 2016):

(49) $$K=\underset{\delta t\to 0}{\lim}\left(\frac{T-I}{\delta t}\right)$$

Once the transition matrix is determined, useful quantities such as the mean first passage time (MFPT), ${\langle t_{fp}\rangle }_{s}$ can be estimated. The MFPT represents the average time for a system to transition into an absorbing state, s. A germane example of an MFPT would be to estimate the folding rate of a protein, where the sth state is the folded end-point and all other states are unfolded intermediates (Dai et al., 2015). This quantity can be determined by:

(50) $${\langle t_{fp}\rangle }_{s}=\sum_{i\neq s}r_{i}$$

where $r_{i}$ is the residence time of state i. The residence times for all states can be determined from the rate matrix K, following the derivation from Polizzi et al. (2016). In their approach, a rate matrix, $\tilde{K}$, is defined that includes an absorbing condition for state s (e.g. $k_{si}=0\;\forall i\neq s$. This allows the residence times in each state to be determined by:

(51) $$r=\int_{0}^{\infty }\text{exp}(\tilde{K}t)\tilde{p_{0}}dt$$

(52) $$\Rightarrow r_{i}={\left[-{\tilde{K}}^{-1}\tilde{p_{0}}\right]}_{i}$$

where $\tilde{p_{0}}$ is the probability distribution with $p_{s}=0$.

The thermodynamics and transition kinetics between the metastable states can then be readily obtained from the MSM model (Qiao et al., 2013). The computational complexity of these simulations is daunting as increasingly larger numbers of states are considered; in general, dimension reduction techniques (e.g. time-lagged independent component analysis (Pérez-Hernández et al., 2013)) and state grouping methods (e.g. k-means clustering) are needed to achieve low-dimensional data and a manageable number of states (Scherer et al., 2015).

*Brownian dynamics (BD)* Brownian dynamics that describe molecular motions of systems with user-interested potentials could provide unique insights into IDP dynamics. When compared to polymer models like the WLC formalism, BD can not only provide ensemble properties like compactness, but also kinetic information such as contact rates, as done by Mühle et al. (2019). In their study, the dynamics of IDPs with different chain lengths were simulated with a minimal BD model, in which IDPs are treated as beads connected by fixed bonds. The authors showed that hydrodynamic interactions and excluded volume effects were key factors that governed the IDP’s dynamics, by comparing experimental measurements of end-to-end contacts obtained from fluorescence correlation data (Mühle et al., 2019). The dynamic properties of higher-order structures, such as those formed by the co-assembly of the conformations of intrinsically disordered Phe-Gly repeats, can also be probed by BD (Moussavi-Baygi and Mofrad, 2016). The fast recovery of high-order structures bestows IDPs with a tolerance to perturbations that may protect their function (Moussavi-Baygi and Mofrad, 2016).

##### Multi-scale approaches.

Multiscale methods can also be used to estimate kinetics. One such example relies on both state discretization (like MSM) and diffusion along a PES (like the Smoluchowski model). This combination was used to reveal the intramolecular rate constants for an IDP of the Sendai virus nucleoprotein (Bernetti et al., 2017). In that study, the PES of the IDP was first obtained by metadynamics simulations. The PES was then used to guide the state discretization to achieve a tractable number of states (Bernetti et al., 2017). The transition rate between two metastable states a to b was then given by:

(53) $$k_{ij}=k_{ij}^{0}\text{exp}\left(\frac{G_{i}-G_{j}}{2k_{B}T}\right)$$

where $G_{i}-G_{j}$ is the free energy difference between two minima and $k_{ij}^{0}$ is the transition rate on a flat PES. Here, $k_{0}$ is a function of a diffusion coefficient D and the barrier height (Eq. (37)). Multiple MD replica simulations were then performed to sample transition events between discretized states to estimate the transition probability matrix (see Eq. (48)). Lastly, kinetic MC simulations based on the transition probability matrix and trial values of D as inputs were performed. The kinetic MC model parameterization that recovered the observed state from the MD replicas was used for determining the appropriate D (Bernetti et al., 2017). Combinations of the computational techniques discussed in this section that have been applied to MAPIDs are summarized in Table 1.

### Computational methods for predicting the MAPID co-assembly

#### Problem and application

Building on the previous sections where the structure or dynamics of isolated MAPIDs are considered, in this section we seek to simulate how intrinsic disorder determines the structures of associated proteins and their rates of binding. This is important for probing how protein structures switch between unfolded and folded conformations that are spontaneous (conformational selection) or induced during binding (induced-fit) (Sugase et al., 2007). Similarly, we can learn how proteins that host many SLIMs within a dynamic sequence can utilize fast on/off binding rates to facilitate high-affinity interactions (Hough et al., 2015). These introduce two main challenges: (1) How to computationally calculate the transition kinetics between biologically relevant states of an IDP and (2) How to relate these kinetics to IDP functions.

IDPs structures and their association kinetics are particularly relevant to myofilament contraction and its dynamic regulation. The binding of TnI’s C-terminal domain with Ca^2+^-saturated TnC is a classical example, whereby the disordered TnI switch peptide undergoes an unfolded to folded transition when bound. Simulating these interactions still needs theoretical developments that can more accurately capture the structures and dynamics of these interactions (Schuler et al., 2020).

#### Experimental techniques

##### Binding assays.

Binding assays are frequently used to assess the association of IDRs with other protein targets. Electrophoresis (see section ‘Experimental techniques’) is routinely used to determine the extent, to which two proteins form a complex, based on differences in the migration of the complex *versus* the isolated components. This assay for instance was used to assess the binding of tropomyosin to tropomodulin (Greenfield et al., 2005; Kostyukova et al., 2007).

F-actin co-sedimentation is another established method to investigate the direct interaction of proteins with the actin filament (Srivastava and Barber, 2008). Co-sedimentation consists of two steps: (1) incubation of purified proteins with actin, (2) centrifugation to pellet actin and analysis of the proteins that co-sediment with actin (Srivastava and Barber, 2008). Co-sedimentation was also used to reveal that PEVK motifs in titin bind to actin in a Ca^2+^-dependent manner (Linke et al., 2002).

Isothermal titration calorimetry (ITC) is a very accurate and commonly used technique capable of measuring the energetics of biomolecules binding over a wide range of affinities (10^−3^ to 10^−12^ M^−1^) (Velázquez-Campoy et al., 2004). This method works by incrementally titrating in a reagent in excess of its binding partner. The heat released from the association event is exchanged with a bath and measured. Analyzing the ITC curves allows the dissociation constant, stoichiometry, enthalpy, and entropy to be determined simultaneously (Velázquez-Campoy et al., 2004). ITC was used to determine the binding affinity of ankyrin’s auto-inhibitory IDR (Chen et al., 2017). These techniques unfortunately do not provide structural and kinetic information about the IDP binding process.

##### Fluorescence spectroscopy.

The fluorescence techniques introduced in section ‘Experimental techniques’ lend themselves to probing the binding between IDRs and their targets (Lee et al., 2015). As one example, time-resolved FRET has been used in tandem with MD simulations to identify functionally important conformations of TnT’s IDR linker when bound to the thin filament (Deranek et al., 2022). Another example monitored differences in intrinsic fluorescence as a probe for conformational changes during TnT/TnC binding; this study also revealed that a DCM-associated mutation in TnC enhanced their binding (Johnston et al., 2019). Intrinsic fluorescence has also been used to probe the interaction between titin’s PEVK motifs and actin in the presence of S100A1 and Ca^2+^ (Yamasaki et al., 2001). The kinetics of assembly are additionally amenable to stopped-flow studies that monitor changes in intrinsic fluorescence following the rapid mixing of two species (Zheng et al., 2015). The time-dependent changes in intrinsic fluorescence can then be fit to rate laws to determine association kinetics (Zheng et al., 2015). Lastly, in recent years, ‘BRET’ techniques that utilize bioluminescence, such as from a luciferase protein donor, to facilitate FRET between a donor/acceptor pair have gained momentum toward characterizing PPIs *in vivo* (De et al., 2013).

##### NMR.

NMR techniques are also heavily utilized for monitoring the kinetics and structures of IDR/protein association. Among these, chemical shifts are the most frequently used to monitor the assembly of MAPID structures. As an example, the binding of tropomodulin’s N-terminal IDR to tropomyosin was studied by collecting ^15^N-^1^H HSQC spectra (Greenfield et al., 2005). The IDR residues exhibit altered chemical shifts upon addition of tropomyosin, therefore allowing the determination of binding sites within the protein’s IDR (Greenfield et al., 2005). Similarly, Hwang *et al*. utilized multidimensional solution NMR spectroscopy to measure complex formation between a TnI fragment (residues M1–G73) and intact TnC. This study indicates that the TnI fragment gains helical content after binding to the C-domain of TnC (Hwang et al., 2014).

#### Other techniques

Other techniques can provide data for probing putative interaction sites between binding species. Mass spectrometry (MS) as an example is an increasingly used technique to determine the binding of IDR-containing species and, in some cases, the amino acids forming the protein/protein interface. Cross-linking mass spectrometry is a popular approach for the latter by identifying adjacent amino acids bridging PPI interfaces (Merkley et al., 2013). Specifically, characterizing chemically cross-linked peptide fragments by mass spectrometry provides residue–residue interaction information. This method has been applied to TnT’s C-terminal IDR binding to TnC, where the direct binding of residues N281–K286 from TnT to TnC residues M1–K6 was determined (Johnston et al., 2019).

Another MS-based technique, HDXMS, is frequently used to probe protein complexes. With HDXMS, protons on buried amino acids undergo less frequent deuterium exchange compared to solvent-exposed sites. Differences in deuterium exchange in isolated proteins relative to the complex provide a topological map of amino acids forming the PPI interface as they are isolated from the deuterated solvent. This approach has been used to determine the binding sites of titin’s N2A isoform to the ankyrin repeat protein (Zhou et al., 2021a). In that study, the authors showed that the N2A segment, together with its C-terminal IDR linker and the Ig-like domain connected by this linker, constitute the binding site for the ankyrin repeat.

CD is another frequently used technique to monitor the association of two species if the isolated and bound states for the proteins exhibit significant changes in secondary structure content. Such applications have been used to probe structural changes following the binding of tropomyosin and tropomodulin (Greenfield et al., 2005; Kostyukova et al., 2007; Uversky et al., 2011).

#### Computational approaches

##### Bioinformatics approaches.

Beyond IDRs ensemble properties, their functions, and especially the binding propensities, are encoded in their sequences (Meng et al., 2017). Empirical observations indicate that regions within IDRs that are predicted to be more ordered can often fold when bound to targets (Oldfield et al., 2005). Correspondingly, IDPs are reported to be enriched in molecule recognition elements (MoREs) that serve as target recognition elements and undergo disorder-to-order transitions upon binding (Yang et al., 2019). SLIMs are equally important in mediating PPIs and are often disordered as well (Davey et al., 2012). The availability of large, annotated IDP datasets and the rapid development of machine learning techniques have resulted in tens of bioinformatic tools for predicting IDPs’ binding sites and propensities for other proteins and nucleic acids (reviewed in Meng et al., 2017). A recent study demonstrated that MoREs and SLIMs in IDPs are amenable to language-processing techniques and are therefore expected to be more readily detected from IDP amino acid sequences (Lindorff-Larsen and Kragelund, 2021). In addition to reasonably accurate predictive models (Meng et al., 2017), the rapid and new developments in this area will provide powerful tools to investigate MAPIDs’ functions.

##### Implicit and semi-analytic representations.

**Fig. 9.**
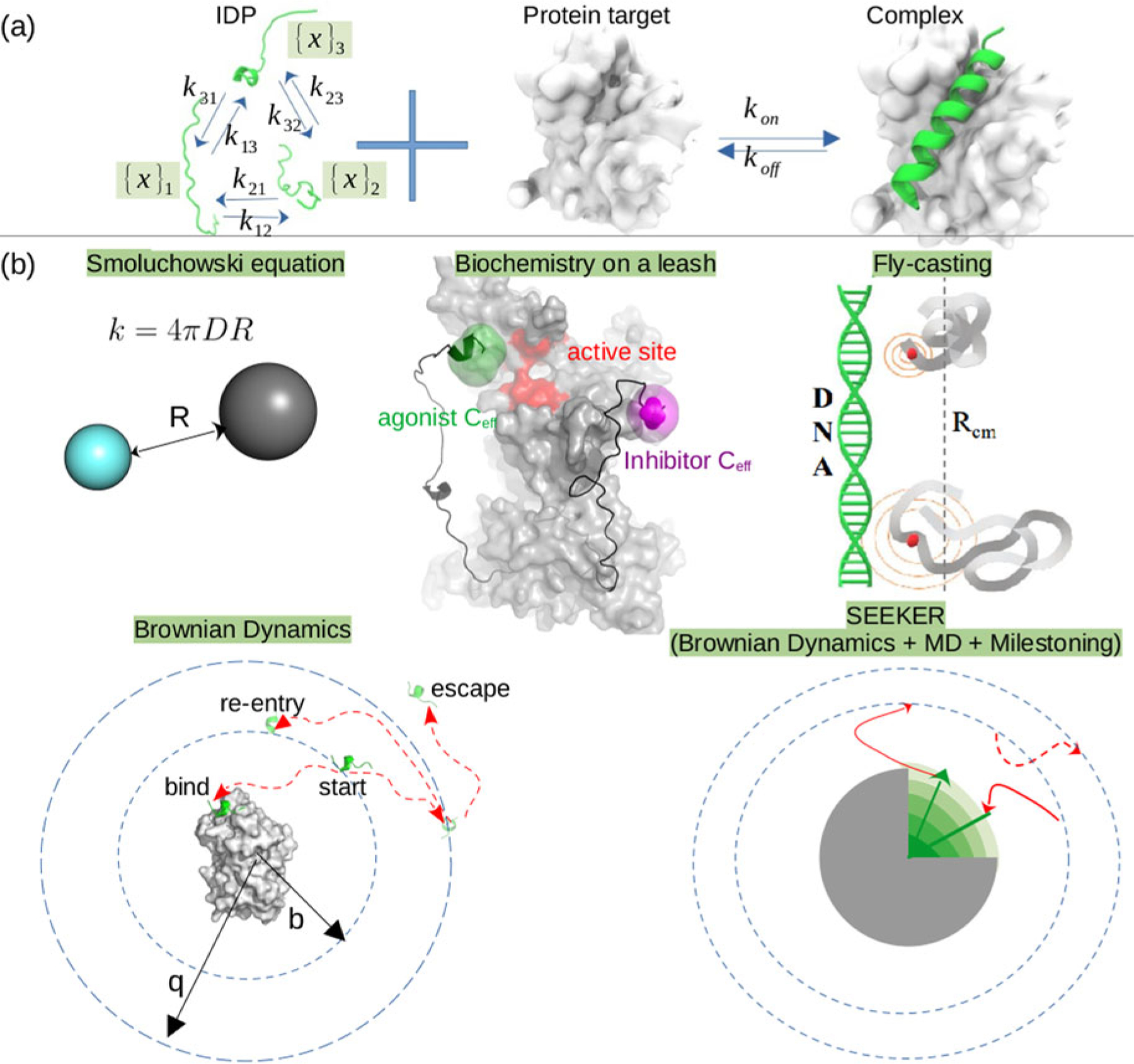
(*a*) Binding of IDP to its partner often goes through a coupled-folding-and-binding process (Sugase *et al*., 2007), in which both the intramolecular conversion kinetics (section ‘Computational methods for predicting intramolecular dynamics of MAPIDs’) of the IDP and its intermolecular association kinetics (section ‘Computational methods for predicting the MAPID co-assembly’) are important. (*b*) Theoretical frameworks for describing IDP intermolecular kinetics. The Smoluchowski equation is an approximation for a diffusion-limited association rate (Kim *et al*., 2018). The Van Valen *et al*. model (Van Valen *et al*., 2009) (biochemistry on a leash) combines IDP-enhanced effective concentrations and competitive binding to describe IDP/target binding (Eqs. (10) and (56)). The fly-casting model (Shoemaker*et al*., 2000) explains the kinetic advantage of IDP/target assembly through its fast searching for binding partners. The Brownian dynamics (BROWNDYE (Huber and McCammon, 2010)) and the SEEKR (Votapka *et al*., 2017) programs both use the Northrup Allison McCammon algorithm (Northrup *et al.*, 1984) provide simulation-based estimates of association kinetics and thermodynamics quantities.

A number of implicit models have been developed for predicting binding affinities and rates for IDRs. Fundamentally, binding affinities can be defined by the relationship:

(54) $$K_{D}=\frac{p_{D}}{p_{A}}$$

(55) $$=\frac{|\Omega {|}^{-1}\text{exp}\left(-U_{D}/k_{B}T\right)}{|\Omega {|}^{-1}\text{exp}\left(-U_{A}/k_{B}T\right)}$$

(56) $$\Rightarrow K_{D}=\text{exp}\left[-\left(U_{D}-U_{A}\right)/k_{\text{B}}T\right]$$

where $U_{D}$ and $U_{A}$ are the free energy of bound and isolated states, respectively. A more rigorous example of note intuitively describes binding affinity in terms of an effective concentration, similar to that introduced in Eq. (10) (Van Valen et al., 2009). This model assumes a protein contains two domains linked by an IDR, for which each domain binds to a distinct site on a target (see Fig. 9). If the affinities are $K_{D1}$ and $K_{D2}$, the combined affinity can be estimated as:

(57) $$K_{D}=\frac{K_{D1}K_{D2}}{c_{\text{eff}}}$$

where $c_{\text{eff}}$ is an effective concentration (Zhou, 2001). For this example, the effective concentration can be determined by an end-to-end probability density, $p\left(d\right)$, such that $c_{\text{eff}}=p\left(d\right)$. We used this concept of effective concentration to characterize TnI/TnC interactions (Siddiqui et al., 2016). Although we did not investigate PTMs in that study, in principle one could use this model to investigate the effects of S23/S24 phosphorylation of TnI on TnC’s Ca^2+$K_{D}$^, which are known to reduce troponin’s apparent Ca^2+^ affinity (Rao et al., 2014).

Avidity is another term often used to describe the fuzzy binding interactions between IDPs and their protein partners. IDPs are well-known to bind to proteins through multi-valent interactions through their many linear sequence motifs (MoRE/SLIM) (Davey et al., 2012; Harmon et al., 2017; Yang et al., 2019). In this regard, there likely exist many binding intermediates with variable stoichiometries. The concept of avidity is proposed to describe the effective binding constant for multivalent interactions between two molecules, and can be quantified by Erlendsson and Teilum (2021):

(58) $$K_{av}=\frac{\sum_{i}\left[RL_{i}\right]}{\left[R_{free}\right][L]}$$

where $\left[R_{free}\right]$ and [L] are the concentrations of free state receptor (folded proteins) and ligand (IDPs), respectively. $\left[RL_{i}\right]$ is the concentration of an intermediate complex with i ligands bound. This equation illustrates that the binding constant increases with the number of bound ligands or motifs.

For the kinetics of binding we introduce the mass action relationship:

(59) $$\text{A}+\text{B}\underset{{\text{k}}_{-}}{\overset{{\text{k}}_{+}}{⇌}}\text{A}\cdot \text{B}$$

where $k_{+}$ represents the association rate constant of species B with target A, while the complex dissociation rate constant is given by $k_{-}$. They are related to the equilibrium constant $K_{D}$:

(60) $$K_{D}=\frac{k_{-}}{k_{+}}$$

In the event that A and B are freely diffusing, spherical, and uniformly reactive, the association rate can be described by the Smoluchowski equation:

(61) $$k_{+}=4\pi DR$$

where D is the diffusion constant and R is the distance between the substrates’ centers of mass. This relationship arises from the diffusion equation:

(62) $$\frac{d\rho (t)}{dt}=-\nabla \cdot j(t)$$

(63) j=−D∇ρ

where the latter equation describes the substrate flux across an arbitrary boundary, such as near the PPI interface. An area integral of the substrate flux over the reaction boundary, $\Gamma_{a}$, yields the association rate

(64) $$k_{+}=\frac{1}{\rho_{0}}\int_{\Gamma_{a}}j\cdot \hat{n}d\Gamma$$

where $\rho_{0}$ is the substrate concentration far from the molecular complex and $\hat{n}$ is a surface normal along $\Gamma_{a}$ (Kekenes-Huskey et al., 2012). For a spherical binding partner, the form:

(65) $$k_{+}=4\pi D{\left[\int_{R_{0}}^{\infty }R^{-2}dR\right]}^{-1}$$

is commonly used, where $R_{0}$ is the radius of a uniformly reactive, spherical protein target (Shoemaker et al., 2000). Evaluation of the integral yields Eq. (61).

To account for a PMF between the binding proteins, u, the Smoluchowsi equation (Eq. (37)) yields a flux

(66) j=D(∇ρ+βρ∇u)

that could be used with Eq. (64). If the reactive center and the PMF are centrosymmetric, Eq. (65) becomes

(67) $$k_{+}=4\pi D{\left[\int_{R_{0}}^{\infty }R^{-2}\text{exp}[\beta u(R)]dR\right]}^{-1|}$$

(Shoemaker et al., 2000). These Smoluchowski relationships have been used for several studies of IDP association (Dogan et al., 2015; Kim et al., 2018; Ozmaian and Makarov, 2019).

To reflect a tethered substrate such as the N-terminal IDR from TnI, arguments similar to those for Eq. (57) may be assumed. Hence, the effective binding rate, $k_{+,\text{eff}}$, of a species A to B to form the complex C can be described via the scheme

(68) $$\text{A}+\text{B}\underset{\text{k}-}{\overset{{\text{k}}_{+}}{⇌}}\text{A}\cdot \text{B}\underset{{\text{k}}_{d}}{\overset{{\text{k}}_{a}}{⇌}}\text{C}$$

for which the first equilibrium represents the diffusional encounter of the proteins from large distances, while the second equilibrium describes the intrinsic rates for forming C from the ‘encounter’ complex A⋅B. The effective binding rate constant can then be evaluated as (Van Valen et al., 2009)

(69) $$k_{+,\text{eff}}=\frac{k_{+,1}k_{a}^{*}}{k_{-,1}+k_{d}^{*}}$$

(70) $$k_{a}^{*}=k_{a}p(R)$$

The latter equation represents the impact of the linker region on the basal association rate of the ‘untethered’ binding domain. Here, p(R), can be interpreted as an effective concentration, such as the expression introduced earlier in Eq. (10) (Van Valen et al., 2009). $k_{+,1}$ and $k_{-,1}$ represent the diffusion-influenced association rate constants from the first equilibrium in Eq. (68), while $k_{a}$ and $k_{d}$ reflect the intrinsic association and dissociation rate constants.

An additional approach characterizes IDR association by a fly-casting mechanism, via which an unfolded domain can accelerate binding to a target (Shoemaker et al., 2000). It relies on computing the flux, $j_{a}$, for a molecule in unfolded, u, and folded, f, states to yield a combined association rate:

(71) $$k_{+}=j_{u}M_{u}+j_{f}M_{f}$$

where $M_{a}$ represents the fraction of species in state a and $j_{a}$ follows from Eq. (66). With this model, the authors demonstrated that binding kinetics can be accelerated by casting unfolded segments toward the target that bind with weak affinity, after which the protein can reel itself toward the target by the simultaneous binding and folding of the segments.

##### Explicit representations.

The kinetics of binding an intrinsically disordered species to a target presents a difficult modeling challenge. This is because of the range of spatio-temporal scales necessary to describe the event. In principle, one can simulate the assembly of two binding species explicitly using unbiased molecular dynamic simulations. Recently, this was demonstrated for the binding of the measles virus nucleoprotein IDP to the X domain of the measles virus phosphoprotein using specialized computing resource Anton for hundreds of μs-length simulations (Robustelli et al., 2020). In this capacity, the association kinetics, given sufficient binding events are observed from the unbiased simulations, can be simply estimated by collecting the number of events per unit time in a fixed volume, such as via $k_{+}=1\text{/}t_{fp}$ where $t_{fp}$ is the first passage time of reaching the bound state (Sun and Kekenes-Huskey, 2021).

The survival probability provides another means for computing the association rate. This can be done by simulating an ensemble of substrates within a closed volume that can collide with a target (Kim and Yethiraj, 2010). This is given by the reaction:

(72) $$\frac{dS_{R}}{dt}(t)=-k(t)C_{L}S_{R}(t)$$

where S(t) is the survival probability, e.g. the likelihood that the target remains unbound from 0 to time t. The probability of the target remaining unbound is enumerated from many simulation trajectories as a function of time. The $k_{+}$ at t→∞ is determined by fitting the expression and extrapolating to large t:

(73) $$\frac{d\ln\left(S_{R}\right)}{dt}=-k(t)C_{L}$$

Hence, the rate can be determined from the survival probability for an element of the disordered domain, such as a SLIM, to remain affixed to a binding site. To our knowledge, such approaches have not been applied to IDRs, or at least not for MAPIDs. This approach generally requires a large number of simulations to obtain convergent results.

Alternatives to explicit, brute force simulations can provide more efficient means for extracting binding kinetics. Biased all-atom MD simulations are a more scalable method to estimating binding kinetics, by favoring trajectories most likely to lead to reaction. As an example, weighted ensemble all-atom simulations were used to obtain a sufficient number of association events between the p53 IDP to its partner, for which the association rates were directly calculated based on a two-state model, e.g. Eq. (59) (Zwier et al., 2016).

A related approach places substrates on a reaction boundary (such as the *b* sphere in BD simulations shown in Fig. 9) and evaluates the flux of substrate toward the reactive center based on explicitly simulating binding trajectories. This allows $k_{+}$ to be determined by (Vijaykumar et al., 2016):

(74) $$k_{+}=\frac{k_{a,0}p_{eq}(R)k_{D}}{k_{a,0}p_{eq}(R)+k_{D}}$$

(75) $$=\left[1-S_{rad}(t\to \infty |R)\right]k_{D}$$

Here $k_{D}$ is used to represent the diffusion-limited association rate between binding partners, $k_{a,0}$ is an intrinsic association rate, and $p_{eq}$ is the equilibrium distribution of the substrate. They also show that this association rate can be determined from $S_{rad}$, which is the survival probability that reflects the likelihood that two partners in contact (radially) diffuse toward the bulk instead of binding. Hence, Eq. (75) describes the process of two partners encountering one another at the diffusion-limited association rate, then binding, before dissociation can occur.

The authors also show that ${\text{k}}_{-}$ can be determined by the flux of substrate away from the binding center (Vijaykumar et al., 2016)

(76) $$k_{-}=k_{d,0}S_{rad}(t\to \infty |R)$$

(77) $$=\frac{\phi_{B,0}}{{𝓗}_{B}}P\left(r_{n}|r_{0}\right)$$

Here, $k_{d,0}$ is an intrinsic dissociation rate, $\phi_{B,0}$ quantifies the flux of substrate reaching a binding interface, ${𝓗}_{B}$ is an indicator if the substrate were recently bound (Vijaykumar et al., 2016), and $P\left(r_{n}|r_{0}\right)$ is the probability that the substrate escapes before being rebound. Formulations that use a biasing force to drive reactions are also proposed, which could additionally enable one to predict dissociation rates from MD simulations (Maximova et al., 2021).

##### Markov state modeling.

MSMs also provide a convenient framework to model the kinetics of assembly, when the RC can be partitioned into a series of states. To relate these kinetics to an association or dissociation rate, the transition probability matrix can be used to compute quantities such as the MFPT or survival probability. When $s_{i}$ and $s_{j}$ represent the bound and dissociated states, the MFPT (〈t〉j) for releasing a bound ligand can be determined, if $s_{j}$ is enforced to be an absorbing state. This residence time is inversely proportional to the off-rate, e.g. $k_{-}=1\text{/}\langle t\rangle j$. SEEKR is one such approach (Votapka et al., 2017, 2022) that has used this formalism to determine an association rate of 9 × 10^8^ M^−1^s^−1^ for Ca^2+^ binding to TnC (Votapka and Amaro, 2015).

##### Brownian dynamics.

BD offers a helpful compromise in modeling IDP/protein association by representing solvent effects through a friction coefficient that acts on the solute (see Eq. (23)). Estimating the association rate from this formalism entails enumerating the number of successful collisions between two species *versus* those that result in the two species diffusing apart. While many approaches are available for estimating this rate, we refer to the frequently used Northrup Allison McCammon algorithm (Northrup et al., 1984) (see Fig. 9).

(78) $$k_{+}=k_{+,1}p$$

where b is the sphere radius at which the interactions between the reactants become centrosymmetric, $k_{+,1}$ is the rate constant of arriving at b, and p is the probability that the reactants bind, instead of dissociating. To calculate p, the term $\beta_{\infty }$ is defined to represent the probability of two reactants reaching b and having a single collision. This leads to two consequences: (1) bind/react with probability α, or (2) escape. For the escaped reactant, there is a probability $\Delta_{\infty }$ to re-collide (encounter the b sphere once again). This process is repeated to obtain p by (Northrup et al., 1984):

(79) $$p=\beta_{\infty }\alpha +\beta_{\infty }(1-\alpha )\Delta_{\infty }\alpha +\beta_{\infty }{\left(1-\alpha \right)}^{2}\Delta_{\infty }^{2}\alpha +\ldots \;\;\;\;=\frac{\beta_{\infty }\alpha }{1-(1-\alpha )\Delta_{\infty }}$$

where α is the probability for the reactants to bind/react after one collision. Details of this derivation are provided in section S2. This approximation is used in the BD code browndye (Huber and McCammon, 2010). We leveraged this relationship to perform association calculations between an IDR of CN with CaM to demonstrate that increased electrostatic screening due to ionic species (see Eq. (9)) slows the diffusion rate (Sun et al., 2018).

BD has also been used to show how an IDR accelerates the binding of EtsΔ 138 protein to ERK2 protein (Misiura and Kolomeisky, 2020). Specifically, the EtsΔ138 has two binding sites for ERK2, one in its folded domain, and another in an IDR domain tethered to the folded domain. BD simulation showed that binding the IDR site accelerates the overall association rate. Increasing the IDR length amplifies the acceleration effect up to ∼4-fold (Misiura and Kolomeisky, 2020).

#### Multi-scale approaches

AAMD and CG techniques have also been widely applied to study IDP binding processes (Liu et al., 2017; Chu et al., 2020; Sun and Kekenes-Huskey, 2021). For instance, in Wang et al. (2013), AAMD was used to reveal the free energy landscape of a 20-residue IDR from measles virus nucleoprotein and its binding mechanism with a protein partner. To accelerate the simulation of the binding process, a structure-based potential was used to augment the conventional all-atom AMBER FF99SB-ILDN potentials to bias the sampling toward the complex state (Wang et al., 2013). These atomic simulations show that binding occurs through a coupled-folding-and-binding process that consists of an initial complex formation via a conformational selection mechanism and a subsequent downhill induced fitting step (Wang et al., 2013). Using a biasing principle, the structure-based model (SBM) is a CG technique that drives binding to restore interprotein contacts present in the native complex. In Chu et al. (2020), SBM CG simulations were used to reveal the role of non-native electrostatics in an IDP/receptor encounter complex. Liu et al. (2017) used a double-bell-potential SBM to reveal that skeletal muscle myosin light chain kinase and CaM bind through a process utilizing both induced fit and conformational selection mechanisms.

When two binding species are well-separated, details of solute–solvent interactions are less important. Electrostatic interactions, however, remain important and influence different stages of an IDR’s binding process. Sun et al. (2018) utilized BD for well-separated species and AAMD when proteins were loosely bound. This approach revealed that electrostatics both drive pCaN toward CaM and determine the conformation exchange kinetics of the isolated IDR ensemble. Another example is from Chu et al. (2012) in which SBM coupled with Debye–Huckel theory was used to evaluate the role of electrostatics in IDP binding. Their simulations demonstrated that electrostatic interactions initially accelerate binding, after which they hinder transitions to the native complex. In these simulations, as the diffusing ensemble of IDR conformations approaches the target, the binding process entails significant refolding to form the final complex. Estimating the kinetics of refolding likely requires atomistic representations.

In the event that two species fold via a conformation selection mechanism, it is possible to estimate the association rate by combining the ‘loose encounter’ association rate with the kinetics of exposing the (IDR) active site, such as a SLIM. Here it is assumed that the IDR has two states, the active state that is capable of binding the partner, and the inactive state in which the binding site is concealed. The rate constants $k_{f}$ and $k_{b}$ reflect the gating kinetics between these two states. The effective association rate for this situation can be estimated via a gating model developed by Szabo et al. (1982):

(80) $$k_{\text{eff}}^{+}=\frac{k_{+}k_{eq}k_{b}Z\left[k_{f}+k_{b}\right]}{k_{f}\left(k_{eq}+k_{+}Z\left[k_{f}+k_{b}\right]\right)+k_{b}Z\left[k_{f}+k_{b}\right]\left(k_{+}+k_{eq}\right)}$$

with

(81) $$Z\left[k_{f}+k_{b}\right]=1+{\left(\left(k_{f}+k_{b}\right)R^{2}/D\right)}^{1/2}$$

where $k_{+}$ is the association rate when the ligand (IDR) is locked into the active state. $k_{eq}$ is a characteristic constant indicating the extent to which the association is diffusion-controlled (see Szabo et al. (1982) for more details). In Sun et al. (2018), we showed using MSMs that the gating of a phosphatase IDR was rapid, which minimized the effect of conformational gating on the association rate. In this way, dynamic ensembles can achieve association kinetics that rival folded proteins.

### Current limitations and future outlook

#### Limitations

##### Force field accuracy.

The numerous IDR modeling approaches described in our review invite an extensive list of limitations and directions for improvement that would benefit MAPID characterization. However, we focus our discussions on atomistic-resolution simulations, which we believe are primed to capture both global structural properties of IDRs and their local attributes. In this regard, improving the force field accuracy for IDP simulations remains one of the foremost challenges in IDR modeling. The flat PES renders IDPs sensitive to force field inaccuracies, as minor errors can drive sampling far from their native ensembles (Pietrek et al., 2020). Here, one key avenue to improve all-atom force field accuracy for IDP simulations rests with protein–water interactions (solvation) and backbone dihedral terms, as tuning these interactions leads to improved agreement between simulations and experiments (Best et al., 2014; Song et al., 2017). Recent efforts aiming to improve models for continuum solvation (i.e. ABSINTH (Vitalis and Pappu, 2009)) or capturing main chain interactions via pseudo-improper-dihedral terms (Mioduszewski et al., 2020) for IDPs are also promising. Developing accurate IDP force fields is an ongoing challenge, and we refer readers to excellent reviews covering the use of experimental data in IDP force field development (Chan-Yao-Chong et al., 2019) and strategies for improving IDP force field accuracy (Mu et al., 2021).

Since the IDRs discussed in this review are adjacent in sequence to well-folded proteins, a secondary goal in IDP force field development is to preserve force field accuracy for the folded constituents. As it has been increasingly realized that many proteins contain both folded and disordered components, developing force fields that are accurate for both IDPs and folded proteins is necessary. Mainstream all-atom force fields such as those from Amber (Weiner et al., 1984) or CHARMM (MacKerell et al., 1998) initially had difficulties in simultaneously characterizing IDP and folded proteins, but refinement of the parameters was shown to be helpful (Robustelli et al., 2018). Similar developments are in progress for CG force fields like maximum entropy optimized force field (MOFF) (Latham and Zhang, 2021), which was parameterized against experimentally measured radius of gyration for IDPs and the folded structures of proteins. This was found to work well for both IDP and folded proteins (Latham and Zhang, 2021).

##### Simulation approaches.

Simulation techniques must evolve in parallel with force field developments. It is increasingly clear that ensemble properties of isolated IDPs can be characterized reasonably well by MD simulations coupled with advanced sampling techniques such as REST, HREMD, or with the help of experimentally guided constraints. However, IDP systems with higher complexity and degrees of freedom, such as IDP-mediated LLPS (Garaizar et al., 2020; Shea et al., 2021), IDP-based extracellular matrix (Clarke and Pappu, 2017) formation, and IDP-aggregation (Strodel, 2021) still would benefit from more advanced computational resources. This can take the form of advanced hardware and chip architectures, much like the recent GPU revolution in MD simulation. Algorithmic changes, such as the recent adoption of hydrogen mass reweighting to increase simulation step size (Hopkins et al., 2015), should be developed in tandem with hardware advances.

##### Structural data.

In addition to modeling developments that could improve IDR modeling as a whole, there are new frontiers specific to the characterization and prediction of MAPID properties. At the time of this writing, there are few reports describing the application of computational techniques to the IDRs of the MAPIDs in Table 1, which renders difficult the probing of the molecular mechanisms driving myofilament function. A key barrier to these applications has been the lack of structural data for the well-folded portions of many of these proteins in isolation, much less in macromolecular complexes like troponin. The configurations of the globular proteins provide important boundary conditions for disordered domains that are tethered to or between domains. Furthermore, knowledge of the higher-order structure of macromolecular complexes is necessary to define the environment, within which an IDR ensemble samples. Advances in cryo-EM spectroscopy and SAXS studies of filament and Z-disk constructions (Sponga et al., 2021; Wang et al., 2021) are quickly closing this gap. Furthermore, recent advances in *ab initio* protein structure programs such as alphafold and rosettafold are providing reliable structures for well-folded domains (Baek et al., 2021; Jumper et al., 2021). In time, these *ab initio* programs are likely to achieve similar gains with IDRs of at least isolated proteins. Strategic combinations of experimental and computational advances may begin to realize three-dimensional atlases of myofilament complexes. Efforts underway in the Tardiff and Schwarz labs to assemble thin filament proteins based on cryo-EM structural data (Mason et al., 2021; Deranek et al., 2022) are already bringing this goal into fruition.

In a similar regard, consideration of auxiliary proteins with IDRs that regulate myofilament function will be essential to understand how the myofilaments adapt to physiological (exercise, pregnancy) and pathological influences (elevated blood pressure, myocardial infarction) (Cornwell et al., 2001; Hamdani et al., 2008). As an example, CaMKII and CN are both implicated in pathological cardiac remodeling, predominantly due to impacting gene transcription (Maier and Bers, 2002). However, CaMKII is additionally reported to phosphorylate titin (Hidalgo et al., 2013), MyBPC3, and TnI (Tong et al., 2004), which likely impacts cardiac contractility on a much more rapid basis (Huke and Bers, 2007). Meanwhile, Calcineurin is tethered to the Z-disk via calsarcin; and given the fundamental importance of the Z-disk to mechanosignal transduction (Russell and Solís, 2021), an intriguing possibility is that the phosphatase may directly impact myofilament function through local PTMs. Both proteins also contain IDRs (Rumi-Masante et al., 2012; Bhattacharyya et al., 2020) that regulate their enzymatic functions, which invites the application of the IDR modeling techniques we discuss in this review. Additional proteins include protein kinase A, protein kinase C, and S100 proteins that are already well-established to impact myofilament function (de Tombe, 2003; Duarte-Costa et al., 2014).

Lastly, the computational methods we discuss in this review almost exclusively deal with dilute environments that are an idealized representation of the cell cytoplasm. Determining how these proteins behave in cellular structures like the sarcomere will also be important for understanding myofilament function and kinetics, as well as the availability of substrates such as ATP for force generation.

#### Open questions

Aside from the challenges we outlined in the previous section, there are a number of open questions with the potential to be resolved via computer simulation. These include what fraction of IDRs in the myofilament serves important functional or regulatory roles? Is there an evolutionary basis for why IDRs are broadly distributed throughout myofilament proteins? The high propensity of IDRs estimated via PONDR would suggest that there are many molecular mechanisms modulating myofilament function, so are they all necessary or merely redundant? In addition, is it possible to determine if only a subset of available conformations in an ensemble is important to function? To what extent do the conformational ensembles of isolated proteins or complexes behave similarly to their counterparts in higher-order complexes such as the troponin complex, the Z-disk, or the myofibril? How do we identify which experiments would yield the most helpful information for characterizing conformation ensembles and constraining conformations for modeling? Among the unique challenges of myofilaments introduced in the section ‘General challenges in characterizing native IDR structures’, is the state-of-the-art in experimental characterization sufficient to determine the important mechanisms of myofilament contraction or are advances still needed? For diseases that have causative origins in single nucleotide polymorphisms located in IDRs, can the severity of an IDR-localized mutation or its potential to be therapeutically corrected be estimated?

#### Future opportunities

##### Artificial intelligence-based predictions of MAPID IDRs.

Atomistic simulations in particular have made considerable advances in enabling the prediction of IDP ensembles. Nonetheless, given the complexity of these proteins and their rapid dynamics, routine, brute force simulations of large disordered regions will remain out of reach for some time. Here machine learning approaches that have demonstrated success in predicting the structures of globular proteins, such as ALPHAFOLD (Jumper et al., 2021) and ROSETTAFOLD (Baek et al., 2021), will likely follow suit with IDPs. As a recent example (Gupta et al., 2022), ‘autoencoders’ were informed from short MD simulations to predict NMR chemical shift and SAXS data. As SAXS and shift data become increasingly available for MAPIDs, such autoencoders could be retrained or refined to improve predictions for myofilament proteins. It also has been proposed that an IDR’s sequence encodes more information than just their ensemble properties (Lindorff-Larsen and Kragelund, 2021). Given that more than 2000 SLIMs have been identified in IDRs, their interactions to form complex, condensates, and disease-related variants are also likely to be predicted from the sequences with advanced machine learning techniques (Lindorff-Larsen and Kragelund, 2021).

##### Multi-modal structure determination and modeling.

In the last decade, advances in cryo-EM have afforded high-resolution structures of macromolecular structures (Bai et al., 2015). A prime example is the near complete model of the thin filament (Yamada et al., 2020), as well as the major constituents of the thick filament (Daneshparvar et al., 2020). These represent remarkable advancements toward the ambitious goal of a complete reconstruction of the entire sarcomere. Budding efforts to image the Z-disk to similar resolutions are ongoing (Wang et al., 2021) and thus it represents the last significant, and likely most challenging, macromolecular myofilament complex to resolve. From a modeling standpoint, the availability of comprehensive MAPID assemblies will present new challenges. The immense computational expense of MD simulations already renders difficult all-atom simulations of isolated proteins at biologically relevant timescales.

Simulations of the full cardiac thin filament of ∼880 kD comprising actin monomers, the troponin complex, and tropomyosin are limited to tens of ns (Mason et al., 2021; Deranek et al., 2022). Hence, these detailed simulations represent a billionth of the duration of a typical 1 s heart beat. For this reason, precise questions and judicious choice of modeling approaches are important considerations when seeking to extrapolate molecular predictions to myofilament function. Especially as more structural data of the myofilament become available, further development of multiscale modeling approaches will be necessary to accommodate predictions from disparate simulation approaches suitable for different system sizes and timescales. Here, efforts to reconstruct dynamic models of whole-heart function from cell-based descriptors (Trayanova and Rice, 2011; Niederer et al., 2019; Timmermann et al., 2019), and myofilament contraction from filament-level molecular interactions (Fenwick et al., 2017; Powers et al., 2019; de Winter et al., 2020; Creso and Campbell, 2021; Sharifi et al., 2021; Kosta et al., 2022), could provide essential guidance toward the incorporation of molecular-resolution data from a variety of MAPIDs. Importantly, these approaches represent the first steps toward combining different data sets that can be conflicting and overlapping at different resolutions. Generalized approaches such as the stochastic multiscale model comprising the coarse-graining of atomic structures followed by BD and Langevin dynamics (Aboelkassem et al., 2019), could lessen the user bias in how structures, models, and modeling results are fused. Similarly, Bayesian approaches could be used to help determine model uncertainties for multi-resolution data and where to prioritize data collection (Liu et al., 2008).

##### Liquid–liquid phase transition (LLPS) mediated MAPID functions.

Biomolecules can condense into liquid phases, forming membrane-less organelles or droplets, through the LLPS process (Shea et al., 2021). LLPS domains are increasingly of interest as they provide unique microenvironments for biological processes, such as forming the replication machinery for viruses (Savastano et al., 2020), providing platforms for protein interactions (Nott et al., 2016), and enriching substrate concentrations (O’Flynn and Mittag, 2021). IDPs are major players in LLPS, because IDPs tend to form multi-valent PPIs with themselves or with other proteins, thus driving LLPS (Harmon et al., 2017). In the sarcomere, LLPS may also serve an important role in mediating protein interactions. For instance, Sponga *et al*. showed that in the sarcomere, the IDP protein FATZ-1 condenses into a liquid phase that may provide a mechanism for its interaction with α-actinin (Sponga et al., 2021). Understanding LLPS in the context of the myofilament is important but is in its infancy, in part because characterizations of MAPIDs are incomplete, and the simulation techniques needed to describe this condensed matter phenomenon are non-trivial. This will improve with more experimental characterizations of MAPIDs, and computational tool development.

##### Genetics.

Ultimately, a compelling motivation for understanding the detailed molecular mechanisms of myofilament function is to contextualize missense variants of myofilament genes and tailor therapeutic intervention. Prominent gene databases such as ClinVar (Landrum et al., 2013) and GnomeAD (Karczewski et al., 2020) have an ever-increasing number of VUSs. The impacts of variants on myofilament function are diverse in phenotype and severity, including the Ca^2+^ sensitivity of force generation or relaxation (Chung et al., 2016; Shafaattalab et al., 2019) that in many cases contribute to cardiac dysfunction (Yar et al., 2014). Efforts to extrapolate these variants to dysfunctional phenotypes via molecular simulations are gaining traction (Shafaattalab et al., 2019; Mason et al., 2021; Sewanan et al., 2021). However, detailed simulations of myofilament proteins and especially their IDRs are computationally intensive. Here, machine learning approaches again may help extrapolate experimental and simulation data from known variants to *de novo* variants (reviewed in Kekenes-Huskey et al., 2022). Combining simulations and experimental characterization of known variants could be used, together with bioinformatics approaches, to identify potential druggable protein/protein or protein/SLIM interactions.

With such developments, we may begin to realize the therapeutic potential for controlling or restoring the intrinsic properties of disordered regions containing pathogenic variants. IDRs can have both pathological and helpful properties, therefore, efforts need to target the conformational ensemble members that primarily promote dysfunction (Uversky, 2020). Progress has been made in IDR-targeted drug design, as was shown for P53 to Mdm2 (Uversky, 2020), a cell cycle regulator, and p27 (Iconaru et al., 2015), a small molecule that binds to a transient IDR site, and the protein tyrosine phosphatase PTP1B, a small molecule that cooperatively binds an IDR (Krishnan et al., 2014). Many other IDP-targeting small molecules have been reported, such as for the c-MyC transcription factor (reviewed in Ruan et al., 2019). These small molecules can achieve binding specificity to an IDP through transient interactions, modulating an IDP’s ensemble properties, and altering protein function (Chen et al., 2020). Thus far, drug design for IDPs has largely targeted specific sites, such as binding pockets or PPIs (Uversky, 2020)

##### Regulatory control.

As remarked in section ‘Properties of MAPIDs and their characterization’, phosphorylation represents one of the most frequently studied PTMs in the myofilament proteins. The PKA, PKC, and CaMKII kinases are among the most common kinase targets activated in the myofilament, in response to β-adrenergic, muscarinic, and calcium signaling (Bers, 2001). In the myofilament, PKA and PKC primarily target troponin (van der Velden and Stienen, 2019). Interestingly, these kinases also contain IDPs (Akimoto et al., 2013; Yang and Igumenova, 2013). Opposing the activity of kinases are phosphatases. Phosphatases including protein phosphatase 1, protein phosphatase 2A (van der Velden and Stienen, 2019), and CN (Rumi-Masante et al., 2012) frequently regulate myofilament proteins. Although phosphatases commonly assume a folded catalytic domain, their regulatory domains as well as regulator proteins such as spinophilin contain IDRs (Marsh et al., 2010; Rumi-Masante et al., 2012).

Understanding regulatory mechanisms controlling the myofilament proteins may therefore benefit from analogous studies of IDRs in the presence of these phosphatases and kinases. Conversely, there may be value in recognizing the role of myofilament proteins in modulating regulatory mechanisms. As an example, CN is modulated by the myofilaments through both α-actinin and calsarcin, which compete for binding CN (Frey et al., 2000; Seto et al., 2013).

## Concluding remarks

The recent decade has unveiled exciting developments in computational and experimental techniques toward resolving the structure and molecular mechanisms of myofilament-associated proteins and their functions. Despite this, only a small fraction of proteins from the myofilament have been resolved at atomistic resolutions. The remaining myofilament proteins have limited structural information. The reviewed topics of IDR structure prediction, ensemble kinetics, and protein co-assembly will undoubtedly provide a basis for characterizing the remaining proteins. However, continued progress toward advancing techniques to overcome many limitations will be essential to mapping gene sequence to function. These advances could help tackle prominent open questions relating to intrinsically disordered proteins that influence myofilament function and dysfunction.

## Supplementary Material

supp

## Figures and Tables

**Table 1.** Brief summary of reported experimental and computational studies on MAPIDs

Gene	IDR region	Experimental characterization	Computational modeling
Thin filament			
ACTC1	Probably whole protein	Native gel analysis (Neirynck et al., 2006)	Bioinformatic analysis (Povarova et al., 2014)
TNNC1	Linker connecting N-/C- domains		Bioinformatic analysis (Na et al., 2016)
TNNI3	N-terminus	X-ray, NMR (Takeda et al., 2003; Hwang et al., 2014)	Conventional AAMD (Cheng et al., 2014; Cheng et al., 2015)
	The switch peptide		Conventional AAMD (Lindert et al., 2015), effective concentration (Siddiqui et al., 2016), accelerated AAMD + effective concentration (Cool and Lindert, 2021)
TNNT2	Residues R158–Q203	FRET (Deranek et al., 2022)	Conventional AAMD (Deranek et al., 2022)
	C-terminus	Cross-link mass spectroscopy (Johnston et al., 2019)	Bioinformatic analysis (Na et al., 2016)
TPM1/TPM3	N-terminus	NMR, CD (Kostyukova et al., 2007)	
LMOD2		X-ray, immunofluorescence imaging, cosedimentation (Tsukada et al., 2010; Colpan et al., 2017; Tolkatchev et al., 2021)	Docking + MD refinement (Tolkatchev et al., 2021)
TMOD1	N-terminus	CD (Kostyukova et al., 2007)	Docking + MD refinement (Tolkatchev et al., 2021)
CTNNA1	Helix bundle E (residues Q260–R360)	X-ray, CD, native gel analysis (Hirano et al., 2018)	
TGM2	IDRs spanning the sequence	X-ray (Pinkas et al., 2007; Kanchan et al., 2015)	Bioinformatic analysis (Thangaraju et al., 2017)
SYNPO2	Most part of the protein	CD, electrophoresis (Khaymina et al., 2007)	Bioinformatic analysis (Khaymina et al., 2007)
ABLIM1	Linkers between LIM domains		Bioinformatic analysis (Ma and Miao, 2020)
Thick filament			
Myosin	MYH7’s S2 motif	CD (Singh et al., 2021)	Homology modeling (Nag et al., 2017)
	LMM domain	Native gel analysis, CD (Parker et al., 2018)	Conventional AAMD (Parker et al., 2018)
	MYH7’s helix motif (residues L693–K707)	X-ray (Houdusse et al., 1999)	
	MYH7’s loop 4 (residues G354–E380)	cryo-EM (Risi et al., 2017)	Cryo-EM constrained modeling (Risi et al., 2017), docking + cryo-EM fitting + MD refinement (Doran et al., 2020)
	MYO6’s two dynamics loops	cryo-EM (Gurel et al., 2017)	
	MYO5a’s coiled-coil structure	CD, ultracentrifugation (Wagner et al., 2006)	
	RLC’s N-terminus (Myosin II)		Conventional AAMD (Espinoza-Fonseca et al., 2007)
	MYL3’s N-terminus	SAXS (Alamo et al., 2017)	Docking + homology modeling (Alamo et al., 2017)
MYBPC3	N-terminal part (majorly concerning the M-domain)	CD, NMR, AFM, SAXS, FRET (Howarth et al., 2012; Colson et al., 2016; Michie et al., 2016; Previs et al., 2016)	Bioinformatic analysis (Lau et al., 2019), conventional AAMD (Colson et al., 2016; Doh et al., 2022), Bayesian inference guided structural modeling based on SAXS data (Potrzebowski et al., 2018)
TTN	PEVK repeats	CD, gel permeation chromatography, gel electrophoresis (Ma and Wang, 2003; Duan et al., 2006)	Conventional AAMD + umbrella sampling + state modeling (Sun and Kekenes-Huskey, 2020), AAMD (Lu et al., 1998; Lee et al., 2007), bioinformatic analysis (Tarnovskaya et al., 2017)
	Linkers between modular units	NMR, HDXMS (Zhou et al., 2021a)	Steered MD (Hsin et al., 2011)
Z-disk			
CRYAB	N- and C-terminus	NMR (Jehle et al., 2010; Baldwin et al., 2012)	Conventional AAMD (Chiappori et al., 2016)
ENH2	LIM domain	NMR (PDB ID 2DAR)	
OBSCN	PDZ domain and linkers	Solution NMR (PDB ID 2EDH)	Conventional AAMD (Whitley et al., 2019)
MYOT	N-domain and C-terminus		Bioinformatic analysis (Puž et al., 2017)
MYOM1	IDRs spanning the sequence	AFM, electron microscopy, CD (Schoenauer et al., 2005)	Bioinformatic analysis (Mészáros et al., 2018; Lau et al., 2019)
DES	N- and C-terminus		Bioinformatic analysis (Anbo et al., 2019)
FHL2	LIM domain	NMR (PDB 2D8Z)	
NEB	IDRs spanning the sequence		Bioinformatic analysis (Wu et al., 2016)
Miscellaneous			
ANK2	C-terminus	CD, binding assays, X-ray (Abdi et al., 2006; Chen et al., 2017)	
FLNC	Filamin domain	NMR (PDB 2D7O)	
MYOZ2	Whole MYOZ1	Binding assays, CD, NMR, X-ray, SAXS (Sponga et al., 2021)	SAXS-based structure modeling (Sponga et al., 2021)
SPTB	N-terminus and residues Q1898–E2083	CD, NMR (Park et al., 2003; Long et al., 2007)	

Experimental methods and their observables supporting intrinsic disorder presence are briefly explained. EM: missing density. X-ray: unresolved structure or high B-factor. CD: little to no secondary structure. NMR: secondary structure and IDP-related observables (Brutscher et al., 2015). FRET: ensemble dimensions/dynamics, population heterogeneity. SAXS: compaction $\left(R_{g}\right)$ (Gibbs and Showalter, 2015). AFM: mechanical properties. Gel analysis: larger Stokes radius, slower migration (Schramm et al., 2019).

## Footnotes

1 These are ‘protein changing’ variants, downloaded from ClinVar on 6/2/2022. The ‘disease-associated’ variants are those categorized as ‘pathogenic’ with selection criteria provided; variant of unknown significance (VUS) refer to variants that have not yet been characterized

2 Isoforms with spectra count (SC) >10 were selected. The spectra count is defined as the total number of spectra identified for a protein via mass spectrometry.
