# Supplementary material for: Myofilament-associated proteins with intrinsic disorder (MAPIDs) and their resolution by computational modeling
Source: Q Rev Biophys. Author manuscript; Available in PMC 2024 May 5. (PMC11070111; doi:10.1017/S003358352300001X)
Supplement: supp [file NIHMS1985396-supplement-supp.pdf]

Supplementary Information (SI)

Myofilament-associated proteins with intrinsic disorder (MAPIDs) and their resolution by computational modeling

Bin Sun[1] and Peter M. Kekenes-Huskey[2]

[1] Research Center for Pharmacoinformatics (The State-Province Key Laboratories of Biomedicine-Pharmaceutics of China), Department of Medicinal Chemistry and Natural Medicine Chemistry, College of Pharmacy, Harbin Medical University, Harbin 150081, China

[2] Department of Cell and Molecular Physiology, Loyola University Chicago, IL 60153, United States

* Corresponding author: pkekeneshuskey@luc.edu

S1 Methods

S1.1 Code

Code in support of this project is publicly available at https://github.com/bsu233/bslab/tree/main/2022-MAPID.

S1.2 Protein weight calculations

The protein weight was calculated via the online tool https://www.bioinformatics.org/sms/prot_mw.html with the canonical fasta (homo sapiens) downloaded from Uniprot as input.

S1.3 IDP prediction

The FASTA sequence of each protein was obtained from the UniProt database using the gene name as a search entry. The canonical sequence from homo sapiens (Human) was selected. The fasta sequence served as input for the online PONDR predictor (http://www.pondr.com/) with the VLXT algorithm selected. The raw data of the ponder predicted was downloaded and analyzed by a home-written python script. Note: Different predictors have varying results on the same protein. See Fig. S1 for 9 predictor results on tnnc1 gene as an example.

S1.4 post-translational modification (PTM) site identification

Phosphorylation sites and sites subject to other PTMwere retrieved from the PhosphoSitePlus database.[15]

S1.5 Pathogenic/Likely pathogenic mutants identification

The Pathogenic/Likely pathogenic mutants of each protein were retrieved from the ClinVar database.[28] In the ClinVar search bar, a keyword like 'actc1[gene]' with the filter "Pathogenic/Likely pathogenic" selected to extract all potential disease-related mutations in the actc1 gene. The searching result was downloaded as a txt file. A home-written python script automatically processed this txt file and assigned these mutants to the IDP regions predicted by the PONDER program.

S1.6 Locating IDPs in the state diagram proposed by Pappu's lab

The localCIDER python library was downloaded from https://pappulab.github.io/localCIDER/. This library provides functions to calculate the fraction of charged residues (FCR), net charge per residue (NCPR) and fraction of positively/negatively charged residues (f_+/f_-) etc. The sequences of Ponder predicted IDP regions were extracted by home-written python script. For IDP regions that contain PTM sites, the site(s) was mutated to glutamic acid (E) to mimic the negative charge introduced by phosphorylations. The sequences were then passed to the functions of localCIDER to obtain the (f_+/f_-). These charge fraction values are used to locate the IDPs in the phase-diagram.

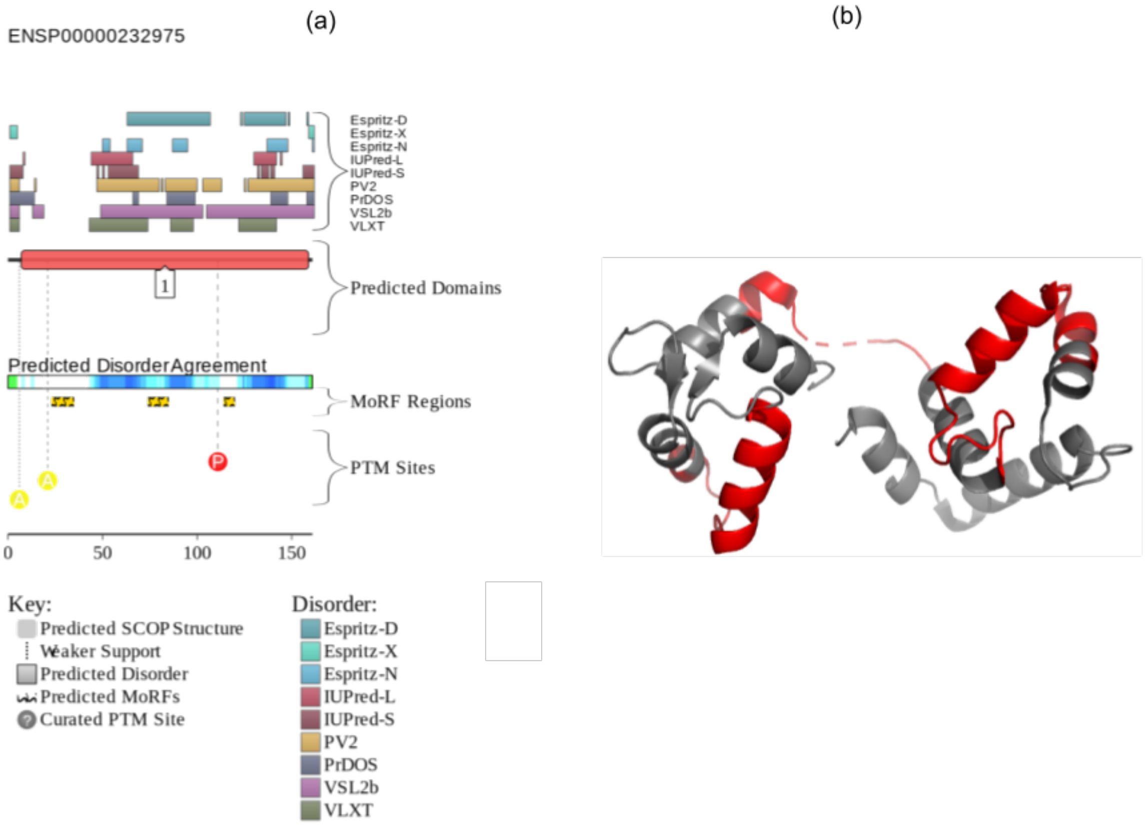

Figure S1: a) Different IDP predictors on TNNC1 gene. Figure is downloaded from the D2P2 database.[475] b) PONDR predicted IDR regions (red) of TNNC1 shown in PDB 1J1E. The predicted IDR regions all contain mobile loops or belong to the N/C terminal linker region

S2 Appendix

S2.1 NAM derivation

Determining the values of β_∞ and Δ_∞ for Eq. 79 in the infinite reactants separation space is impractical but the estimates of these two in finite space β and Δ can be obtained from BD simulations. Specifically, a q sphere is defined, with $q > b$. In a BD simulations, a trajectory is terminated either when the two reactants bind or they reach the q sphere and escape. Thus the q sphere represents a truncated 'infinite' separation space. In real case, the probability reactants escape from b sphere and are freely to diffuse into infinite space is $1 - \beta_\infty$. This probability, in the BD simulations with the q sphere defined, is then:

$$1 - \beta_\infty = (1-\beta)(1-\Omega)[1 + (1-\beta)\Omega + (1-\beta)^2\Omega^2 + ...] = \frac{(1-\beta)(1-\Omega)}{1-(1-\beta)\Omega} \tag{S1}$$

$$\beta_\infty = \frac{\beta}{1-(1-\beta)\Omega} \tag{S2}$$

where Ω is the probability, in the BD simulations, that the reactants reach to q sphere but eventually diffuse back to the b sphere instead of escaping. The Δ_∞ is related to Δ in a similar vein, which gives $\Delta_\infty = \Delta + \beta_\infty(1 - \Delta)$. Therefore, the association rate is expressed as:[414]

$$k = \frac{k_D(b)[\frac{\beta}{1-(1-\beta)\Omega}]\alpha}{1-(1-\alpha)[\Delta + [\frac{\beta}{1-(1-\beta)\Omega}](1-\Delta)]} \tag{S3}$$

β and Δ are determined from BD simulations (in the finite domain), $k_D(b)$ and Ω are calculated by:

$$k_D(b) = \frac{1}{\int_b^\infty dr[\frac{\exp(U(r)/k_BT)}{4\pi r^2 D(r)}]} \tag{S4}$$

$$\Omega = k_D(b)\int_q^\infty dr[\frac{\exp(U(r)/k_BT)}{4\pi r^2 D(r)}] \tag{S5}$$
